# Progression of atypical parkinsonian syndromes: PROSPECT-M-UK study implications for clinical trials

**DOI:** 10.1093/brain/awad105

**Published:** 2023-03-28

**Authors:** Duncan Street, Edwin Jabbari, Alyssa Costantini, P Simon Jones, Negin Holland, Timothy Rittman, Marte T Jensen, Viorica Chelban, Yen Y Goh, Tong Guo, Amanda J Heslegrave, Federico Roncaroli, Johannes C Klein, Olaf Ansorge, Kieren S J Allinson, Zane Jaunmuktane, Tamas Revesz, Thomas T Warner, Andrew J Lees, Henrik Zetterberg, Lucy L Russell, Martina Bocchetta, Jonathan D Rohrer, David J Burn, Nicola Pavese, Alexander Gerhard, Christopher Kobylecki, P Nigel Leigh, Alistair Church, Michele T M Hu, Henry Houlden, Huw Morris, James B Rowe

**Affiliations:** University of Cambridge Department of Clinical Neurosciences and Cambridge University Hospitals NHS Trust, Cambridge, CB2 OQQ, UK; Department of Clinical and Movement Neurosciences, University College London, Queen Square Institute of Neurology, London, WC1N 3BG, UK; Movement Disorders Centre, University College London, Queen Square Institute of Neurology, London, WC1N 3BG, UK; Department of Clinical and Movement Neurosciences, University College London, Queen Square Institute of Neurology, London, WC1N 3BG, UK; Movement Disorders Centre, University College London, Queen Square Institute of Neurology, London, WC1N 3BG, UK; University of Cambridge Department of Clinical Neurosciences and Cambridge University Hospitals NHS Trust, Cambridge, CB2 OQQ, UK; University of Cambridge Department of Clinical Neurosciences and Cambridge University Hospitals NHS Trust, Cambridge, CB2 OQQ, UK; University of Cambridge Department of Clinical Neurosciences and Cambridge University Hospitals NHS Trust, Cambridge, CB2 OQQ, UK; Department of Clinical and Movement Neurosciences, University College London, Queen Square Institute of Neurology, London, WC1N 3BG, UK; Movement Disorders Centre, University College London, Queen Square Institute of Neurology, London, WC1N 3BG, UK; Department of Neuromuscular Diseases, University College London, Queen Square Institute of Neurology, London, WC1N 3BG, UK; Neurobiology and Medical Genetics Laboratory, ‘Nicolae Testemitanu’ State University of Medicine and Pharmacy, Chisinau 2004, Republic of Moldova; Department of Neuromuscular Diseases, University College London, Queen Square Institute of Neurology, London, WC1N 3BG, UK; Department of Clinical and Movement Neurosciences, University College London, Queen Square Institute of Neurology, London, WC1N 3BG, UK; Department of Neurodegenerative Disease, University College London, Queen Square Institute of Neurology, London, WC1N 3BG, UK; UK Dementia Research Institute, University College London, London, W1T 7NF, UK; Geoffrey Jefferson Brain Research Centre, Division of Neuroscience, Faculty of Biology, Medicine and Health, University of Manchester, Manchester, M6 8HD, UK; Wellcome Centre for Integrative Neuroimaging, Oxford Centre for Functional MRI of the Brain, Nuffield Department of Clinical Neurosciences, University of Oxford, Oxford, OX3 9DU, UK; Nuffield Department of Clinical Neurosciences, University of Oxford, Oxford, OX3 9DU, UK; Nuffield Department of Clinical Neurosciences, University of Oxford, Oxford, OX3 9DU, UK; University of Cambridge Department of Clinical Neurosciences and Cambridge University Hospitals NHS Trust, Cambridge, CB2 OQQ, UK; Department of Clinical and Movement Neurosciences, University College London, Queen Square Institute of Neurology, London, WC1N 3BG, UK; Queen Square Brain Bank for Neurological Disorders, University College London, Queen Square Institute of Neurology, London, WC1N 3BG, UK; Reta Lila Weston Institute, University College London, Queen Square Institute of Neurology, London, WC1N 3BG, UK; Queen Square Brain Bank for Neurological Disorders, University College London, Queen Square Institute of Neurology, London, WC1N 3BG, UK; Reta Lila Weston Institute, University College London, Queen Square Institute of Neurology, London, WC1N 3BG, UK; Queen Square Brain Bank for Neurological Disorders, University College London, Queen Square Institute of Neurology, London, WC1N 3BG, UK; Reta Lila Weston Institute, University College London, Queen Square Institute of Neurology, London, WC1N 3BG, UK; Queen Square Brain Bank for Neurological Disorders, University College London, Queen Square Institute of Neurology, London, WC1N 3BG, UK; Reta Lila Weston Institute, University College London, Queen Square Institute of Neurology, London, WC1N 3BG, UK; Department of Neurodegenerative Disease, University College London, Queen Square Institute of Neurology, London, WC1N 3BG, UK; UK Dementia Research Institute, University College London, London, W1T 7NF, UK; Clinical Neurochemistry Laboratory, Sahlgrenska University Hospital, 431 30 Mölndal, Sweden; Department of Psychiatry and Neurochemistry, Institute of Neuroscience and Physiology, The Salhgrenska Academy at the University of Gothenburg, 413 45 Goteborg, Sweden; Hong Kong Center for Neurodegenerative Diseases, Hong Kong Science Park, Shatin, N.T., Hong Kong, China; Department of Neurodegenerative Disease, University College London, Queen Square Institute of Neurology, London, WC1N 3BG, UK; Centre for Cognitive and Clinical Neuroscience, Division of Psychology, Department of Life Sciences, College of Health, Medicine and Life Sciences, Brunel University London, London, UB8 3PH, UK; Dementia Research Centre, Department of Neurodegenerative Disease, UCL Queen Square Institute of Neurology, University College London, London, WC1N 3BG, UK; Department of Neurodegenerative Disease, University College London, Queen Square Institute of Neurology, London, WC1N 3BG, UK; Faculty of Medical Sciences, Newcastle University, Newcastle, NE2 4HH, UK; Clinical Ageing Research Unit, Newcastle University, Newcastle, NE4 5PL, UK; Division of Neuroscience, Wolfson Molecular Imaging Centre, University of Manchester, Manchester, N20 3LJ, UK; Departments of Geriatric Medicine and Nuclear Medicine, Center for Translational Neuro- and Behavioral Sciences, University Medicine Essen, 45356 Essen, Germany; Division of Neuroscience, Wolfson Molecular Imaging Centre, University of Manchester, Manchester, N20 3LJ, UK; Department of Neurology, Manchester Academic Health Science Centre, Northern Care Alliance NHS Foundation Trust, Salford, M13 9NQ, UK; Department of Neuroscience, Brighton and Sussex Medical School, Brighton, BN1 9PX, UK; Department of Neurology, Royal Gwent Hospital, Newport, NP20 2UB, UK; Nuffield Department of Clinical Neurosciences, University of Oxford, Oxford, OX3 9DU, UK; Department of Physiology, Anatomy and Genetics, Oxford Parkinson’s Disease Centre, University of Oxford, Oxford, OX1 3QU, UK; Department of Clinical and Movement Neurosciences, University College London, Queen Square Institute of Neurology, London, WC1N 3BG, UK; Movement Disorders Centre, University College London, Queen Square Institute of Neurology, London, WC1N 3BG, UK; Department of Neuromuscular Diseases, University College London, Queen Square Institute of Neurology, London, WC1N 3BG, UK; Department of Clinical and Movement Neurosciences, University College London, Queen Square Institute of Neurology, London, WC1N 3BG, UK; Movement Disorders Centre, University College London, Queen Square Institute of Neurology, London, WC1N 3BG, UK; University of Cambridge Department of Clinical Neurosciences and Cambridge University Hospitals NHS Trust, Cambridge, CB2 OQQ, UK; Medical Research Council Cognition and Brain Sciences Unit, University of Cambridge, Cambridge, CB2 7EF, UK

**Keywords:** progressive supranuclear palsy, corticobasal syndrome, multiple system atrophy, clinical trials, sample size

## Abstract

The advent of clinical trials of disease-modifying agents for neurodegenerative disease highlights the need for evidence-based end point selection. Here we report the longitudinal PROSPECT-M-UK study of progressive supranuclear palsy (PSP), corticobasal syndrome (CBS), multiple system atrophy (MSA) and related disorders, to compare candidate clinical trial end points.

In this multicentre UK study, participants were assessed with serial questionnaires, motor examination, neuropsychiatric and MRI assessments at baseline, 6 and 12 months. Participants were classified by diagnosis at baseline and study end, into Richardson syndrome, PSP-subcortical (PSP-parkinsonism and progressive gait freezing subtypes), PSP-cortical (PSP-frontal, PSP-speech and language and PSP-CBS subtypes), MSA-parkinsonism, MSA-cerebellar, CBS with and without evidence of Alzheimer’s disease pathology and indeterminate syndromes. We calculated annual rate of change, with linear mixed modelling and sample sizes for clinical trials of disease-modifying agents, according to group and assessment type.

Two hundred forty-three people were recruited [117 PSP, 68 CBS, 42 MSA and 16 indeterminate; 138 (56.8%) male; age at recruitment 68.7 ± 8.61 years]. One hundred and fifty-nine completed the 6-month assessment (82 PSP, 27 CBS, 40 MSA and 10 indeterminate) and 153 completed the 12-month assessment (80 PSP, 29 CBS, 35 MSA and nine indeterminate). Questionnaire, motor examination, neuropsychiatric and neuroimaging measures declined in all groups, with differences in longitudinal change between groups. Neuroimaging metrics would enable lower sample sizes to achieve equivalent power for clinical trials than cognitive and functional measures, often achieving N < 100 required for 1-year two-arm trials (with 80% power to detect 50% slowing). However, optimal outcome measures were disease-specific.

In conclusion, phenotypic variance within PSP, CBS and MSA is a major challenge to clinical trial design. Our findings provide an evidence base for selection of clinical trial end points, from potential functional, cognitive, clinical or neuroimaging measures of disease progression.

## Teaser

Street *et al.* compare candidate clinical trial end points in progressive supranuclear palsy, multiple system atrophy, corticobasal syndrome and related disorders. Neuroimaging metrics generally enable lower sample sizes than cognitive and functional measures, although optimal outcome measures vary by disease and subtype.

## Introduction

Progress in the development of disease-modifying treatments for neurodegenerative disease is hindered by clinical heterogeneity and lack of evidence for the relative merits of alternative endpoints. This problem is exacerbated for rare disorders, even where progress in understanding the mechanisms of disease is leading to new therapeutic strategies. The ‘Progressive Supranuclear Palsy-Corticobasal Syndrome-Multiple System Atrophy’ study (PROSPECT-M-UK) was established to examine the feasibility and sensitivity of alternative end points in progressive supranuclear palsy (PSP), corticobasal syndrome (CBS) and multiple system atrophy (MSA). These are distinct neurodegenerative parkinsonian conditions, with combined prevalence of 10–18 per 100 000 population.^1–3^ Each disease has a characteristic set of clinical presentations and diagnostic criteria.^4–6^ Despite their differences, they are sometimes collectively referred to as ‘atypical parkinsonian syndromes’ (APS), in contrast to Parkinson’s disease (PD).^7^ Truly ‘atypical’ parkinsonian disorders also exist as part of a clinical spectrum, fulfilling some but not all diagnostic criteria for any specific disease.^8,9^ Survival after diagnosis is poor, at an average of 2.9 years in PSP,^3,10–13^ 4.6 years in CBS^3,14,15^ and 4 years in MSA,^13,16^ despite improvements in early recognition and diagnosis.^17–19^

Disease-modifying therapies are urgently required, but success in clinical trials has been elusive.^20–24^ The challenges for clinical trials are increased by limited sensitivity of chosen outcome measures, coupled with short duration and restrictive inclusion/exclusion criteria. An improved evidence base for clinical and biomarker progression measures across the spectrum of PSP, CBS and MSA would facilitate clinical trial design. An ideal clinical trial end point would not only be sensitive to change, but also informative about disease mechanism, applicable across multiple disorders and their phenotypic variants, minimally invasive and scalable in consideration of time, cost and availability. Many outcome measures have been proposed for PSP, CBS and MSA. Disease-specific clinical rating scales, like the Movement Disorders Society Unified PD Rating Scale (MDS-UPDRS),^25^ Unified MSA rating scale (UMSARS),^26^ and the PSP Rating Scale (PSPRS),^27^ have measured progression in observational and interventional cohorts.^28,29^ Alternatives that are, in principle, applicable across multiple disorders include brain imaging (e.g. volumetric MRI measures), neuropsychological performance [e.g. Montreal Cognitive Assessment (MoCA)], quality of life (QoL) [e.g. Schwab and England Activities of Daily Living Scale (SEADL)] and biochemical assays of serum or CSF [e.g. neurofilament light chain (NfL)]. The ‘head-to-head’ comparison of candidate end points would inform the selection of optimum markers according to disease group and phenotype, and the stage of a clinical trial. Such comparisons have been performed in frontotemporal dementia^30,31^ and Huntington’s disease^32^ using the effect size of change for each assay to estimate sample sizes required for a clinical trial. We adopt this approach for the longitudinal PROSPECT-M-UK study.

PROSPECT-M-UK is a multicentre, observational cohort study that aims to develop markers that assist diagnosis, monitor disease progression, and elucidate pathogenesis. The study includes the breadth of defined clinical subtypes of PSP, MSA and CBS, as well as participants with initially indeterminate phenotypes. At baseline,^8^ diagnostic groups showed distinct patterns of functional loss, cognitive decline, regional brain atrophy and fluid biomarker levels. Here we report the performance of clinical, cognitive and imaging end points during longitudinal follow-up. We use observational data over 6- and 12-month time points to estimate the samples size that would be required for clinical trials of disease-modifying treatments using alternative measures, for each disease group and subtype. Subgroup analyses apply principal selection criteria from recent phase 2 clinical trials in PSP (NCT03068468) and MSA (NCT03952806), and test for non-linear progression of the biomarkers.

## Materials and methods

### Study design and participants

The PROSPECT-M-UK study natural history cohort comprises seven UK study sites [University College London Hospital (UCLH), University of Cambridge and Cambridge University Hospitals NHS Trust, University of Oxford, University of Manchester, Newcastle University, University of Sussex, and Royal Gwent Hospital, Wales]. Ethical approval was granted by the University College London research ethics committee. Written informed consent was given by all participants in accordance with the Declaration of Helsinki. Baseline assessment was between 1 July 2015 and 30 September 2019. Participants were invited to register for post-mortem brain bank donation at one of four sites [Queen Square (London), Cambridge, Oxford and Manchester]. Longitudinal study visits were performed at 6 and 12 months. The survival census date was set at 19 February 2021.

### Diagnosis and phenotyping

We present demographics, baseline characteristics and progression data in two complementary formats. First, according to the diagnosis at baseline (as used by Jabbari *et al*.^8^ with additional baseline cases added). Second, according to the final ‘best’ diagnosis that also draws on clinical features and investigations arising during disease progression. The former approximates the approach anticipated in an ‘Intention to Treat’ design, using the working diagnosis at baseline, while the latter diagnoses are more likely to be neuropathologically accurate, and reflect the underlying disease pathology. Pathology-specific biomarkers are anticipated that would improve the accuracy of diagnosis at entry to future trials.^33^ Diagnosis of PSP was initially made according to 1996 NINDS-SPSP criteria, and later revised according to the 2017 Movement Disorders Society criteria^4^; CBS according to the Armstrong criteria^5^; and MSA according to the revised Gilman criteria.^6^ Individuals with an indeterminate phenotype (IDT), who were suspected to have an atypical parkinsonian disorder but not meeting diagnostic criteria, were also recruited and assessed with reference to most recent diagnostic criteria.

At baseline, PSP participants were stratified into PSP-Richardson, PSP-subcortical and PSP-cortical groups. ‘PSP-subcortical’ included PSP-parkinsonism, PSP-progressive gait freezing (PSP-PGF) and PSP-oculomotor; ‘PSP-cortical’ included PSP-CBS, PSP-speech/language and PSP-frontal. These clinically identifiable subgroups have differential prognosis^8,10,12,34^ and reflect differences in the distribution of neuroglial tau pathology.^35^ CBS participants with CSF or amyloid-PET (Pittsburgh compound-B) evidence of underlying Alzheimer’s disease (AD) pathology were defined as CBS-AD (Supplementary material). Those with normal CSF AD biomarker analysis or negative amyloid-PET imaging were defined as CBS-4RT due to the high likelihood of corticobasal degeneration or PSP pathology.^36^ CBS participants without CSF, post-mortem or amyloid-PET examination were defined as CBS-indeterminate (CBS-IDT). MSA participants were classed according to revised Gilman criteria^6^ into MSA-parkinsonism (MSA-P) and MSA-cerebellar (MSA-C) groups. We repeated the principal analyses after additional application of the general inclusion/exclusion criteria from recent phase II clinical trials in PSP (NCT03068468) and MSA (NCT03952806). These general criteria are listed in the Supplementary material.

### Procedures

Data collection and storage are described in the PROSPECT-M-UK baseline report.^8^ Demographic and clinical information included the neurological history and structured examination performed by a physician at each study visit. This assessment allowed for change in diagnosis according to clinical evolution of participants’ phenotype. Questionnaires were completed by participant and/or carer at each visit while neuropsychiatric assessments were administered by research staff. Fluid biomarkers including serum NfL levels, CSF total tau (T-tau) and CSF β-amyloid 1–42 (Aβ1-42) were measured at baseline. DNA was extracted from blood samples and analysis performed for a subset of patients for genotyping and single-nucleotide polymorphism imputation to obtain *MAPT* (OMIM 157140) H1/H1, *APOE* (OMIM 107741) ε4 allele, *TRIM11* rs 564 309 (OMIM 607868) and *LRRK2* rs2242367 (OMIM 609007) minor allele group frequencies. Volumetric T_1_-weighted MRI was obtained at one of three scanning centres (UCL, Cambridge, Oxford) on 3 T Siemens scanners (PRISMA or TRIO systems) and imaging markers were extracted (Supplementary material).

### Statistical analysis

R studio (version 4.0.3, R Core Team, 2020) was used for analysis of demographic and clinical data. Missing observations in individual subscores were imputed using the Multiple Imputation via Chained Equations package (mice)^37^ when >80% of the assessment was otherwise complete. Longitudinal annualized progression for each measure was estimated by construction of a generalized linear mixed effects model in R studio using the lme4 package.^38^ For each measure or score of interest, the main dependent variable was the measure/score, with fixed effects of first measure/score and follow-up interval (without interaction terms). Random effects were identity, under the assumption that intercepts and slope may differ between subjects. Neither normality nor homoscedasticity of residual plots were significantly violated. The annual change of each measure/score (Δy) represents the estimated slope of the linear progression of total score. A standardized effect size was calculated using Δy and its standard deviation (SD), and this was used for sample size calculation per group for a two-sample *t*-test, similar to previous reports.^39^ Sample size calculations were estimated for a two-sided test significance level of 5% and a power of 80%.

Data normality was tested by the Shapiro-Wilk test. Demographic means were compared using independent samples *t*-tests when measures were continuous and normally distributed and Kruskal-Wallis tests when continuous and not normally distributed. When comparing more than two groups, pairwise comparisons were adjusted for multiple testing using the Tukey method when normally distributed, and Benjamini-Hochberg method when not normally distributed. Mean values are expressed with their associated standard deviations. Categorical data were compared using Chi Square tests. Analysis of covariance (ANCOVA) and Tukey *post hoc* tests compared non-neuroimaging variable means between groups adjusting for age at baseline, and neuroimaging variables adjusting for age and total intracranial volume. The log-rank test compared survival curves. For all analyses, a *P* < 0.05 was considered significant. The *survival*^40^ and *survminer*^41^ packages in R studio were used for Cox Survival regression models.

### Data availability

Clinical data from this study including scales, biomarker measurement and genotypes used are available via application to the PROSPECT-M-UK Data Access committee (prospect@ucl.ac.uk). All applications will be reviewed by the data access committee, including PSP Association representatives, the independent chair and study principal investigators.

## Results

### Demographics and phenotyping

Baseline demographic and survival data according to final diagnosis and phenotype are presented in Table 1. Baseline clinical and biomarker characteristics according to final best diagnosis group and phenotype are presented in Supplementary Table 1. Two hundred and forty-three individuals were recruited [117 with PSP, 68 with CBS, 42 with MSA and 16 indeterminate; 138 (56.8%) male; mean ± SD age 68·7 ± 8.61 years]. One hundred and fifty-nine completed 6-months’ assessment (159/243, 65.4%) (82 with PSP, 27 with CBS, 40 with MSA and 10 indeterminate), and one hundred and fifty-three completed 12-months’ assessment (153/243, 63.0%) (80 with PSP, 29 with CBS, 35 with MSA and nine indeterminate).

**Table 1:** Demographics by final diagnosis

	PSP				CBS				MSA			IDT
	All (*n* = 117)	RS (*n* = 57)	Cortical (*n* = 28)	Subcortical (*n* = 32)	All (*n* = 42)	4RT (*n* = 10)	AD (*n* = 10)	Unknow*n* (*n* = 22)	All (n = 68)	Parkinsonism (*n* = 41)	Cerebellar (*n* = 26)	(*n* = 16)
Sex, male:female (%)	62:38	65:35	50:50	69:31	33:67	40:60	40:60	27:73	62:38	52:48	77:23	56:44
Age at enrolment, years ± SD	71.3 (7.0)	69.7 (7.6)	73.0 (6.2)	72.7 (6.1)	67.5 (7.6)	65.8 (9.3)	69.7 (7.8)	67.3 (6.8)	64.5 (9.3)	65.2 (9.2)	73.0 (6.2)	70.1 (11.3)
Age at motor symptom onset, years ± SD	66.8 (7.2)^a^	66.3 (7.2)	73.0 (6.2)	66.0 (7.4)	62.2 (7.7)	59.7 (9.5)	64.5 (7.4)	62.4 (7.0)	59.5 (10.1)	60.5 (10.5)	57.8 (9.3)	66.4 (11.7)
Disease duration at enrolment, years ± SD	4.5 (2.9)	3.5 (2.2)^b^	4.5 (2.0)^c^	6.3 (3.8)	5.3 (3.0)	6.1 (3.1)	5.3 (2.8)	4.9 (3.1)	5.2 (2.7)	4.8 (2.6)	5.9 (2.8)	3.5 (1.4)
Number deceased (% of total)	62 (53%)	32 (56%)	18 (64%)	12 (38%)	16 (38%)	5 (50%)	6 (60%)	5 (23%)	28 (41%)	18 (43%)	10 (38%)	2 (13%)
Survival from onset to death	6.3 (2.7)	5.9 (2.5)	5.6 (1.5)	8.2 (3.7)^d^	8.1 (3.5)	9.1 (3.8)	8.7 (3.9)	6.3 (2.7)	7.8 (3.1)	7.2 (3.0)	8.9 (3.2)	4.6 (1.2)
Number with post-mortem diagnosis	PSP in 12, CBD in 1	PSP in 6, CBD in 1	PSP in 2	PSP in 4	AD in 1, CBD in 1	AD in 1	–	CBD in 1	MSA in 3, AD in 1, PD in 2	MSA in 2, AD in 1, PD in 1	MSA in 1, PD in 1	–

4RT = 4-repeat tau; AD = Alzheimer disease; CBS = corticobasal syndrome; IDT = indeterminate; MSA = multiple system atrophy; PSP = progressive supranuclear palsy; SD = standard deviation.

False discovery rate (FDR) adjusted *P* < 0.01 versus CBS-all and MSA-all.

FDR adjusted *P* < 0.001 versus PSP-subcortical and *P* < 0.05 versus PSP-cortical.

FDR adjusted *P* < 0.05 versus PSP-subcortical.

FDR adjusted *P* < 0.05 versus PSP-cortical and PSP-Richardson’s syndrome.

**Figure 1:**
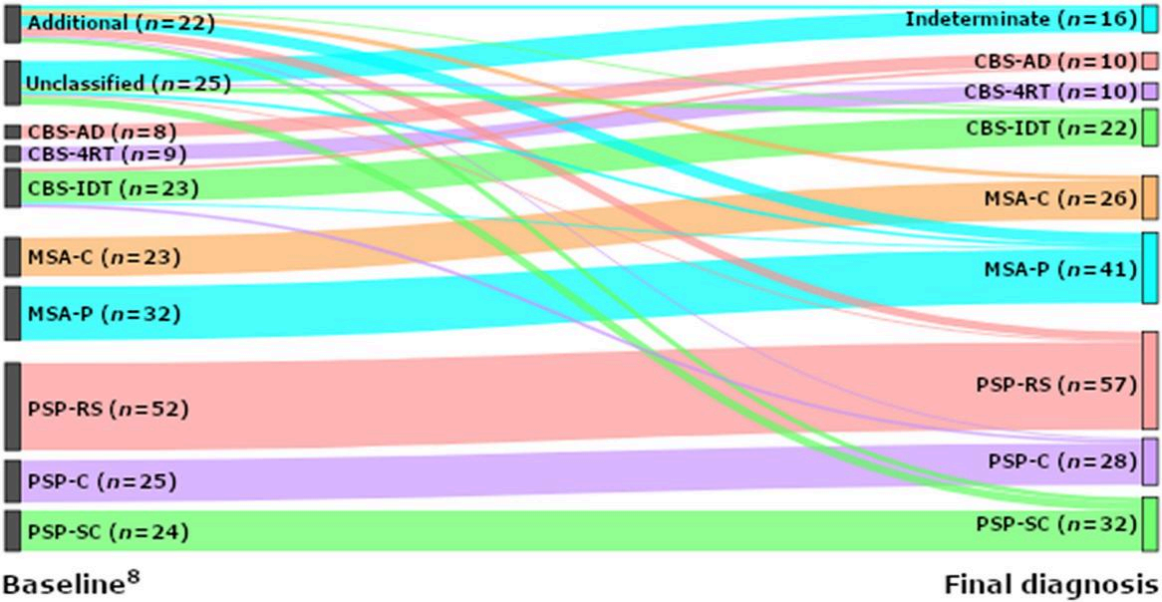
**Sankey plot of change in diagnosis between baseline** ^8^ **and study end point.** 4RT = four-repeat tau; AD = Alzheimer’s disease; CBS = corticobasal syndrome; IDT = indeterminate; MSA-C = multiple system atrophy cerebellar variant; MSA-P = multiple system atrophy parkinsonian variant; PSP = progressive supranuclear palsy; PSP-C = PSP-cortical; PSP-RS = PSP-Richardson’s syndrome; PSP-SC = PSP-subcortical.

The distribution of phenotypes is presented in Fig. 1. A change in clinical diagnosis between baseline and 1 year occurred in 15 cases (15/243, 6.2%): 11 were reclassified from IDT to a recognized diagnosis group (*n* = 4 CBS, *n* = 2 MSA and *n* = 5 PSP), one case changed from CBS to MSA and two from CBS to PSP. One person recovered following an indeterminate classification at baseline and was excluded. In 8/25 (32%) IDT cases, the diagnosis remained indeterminate after 1 year.

On neuropsychiatric assessment, the MSA group had higher baseline UPDRS Part I, MoCA and Addenbrooke’s Cognitive Examination III (ACE III) scores than the CBS and PSP groups, while the PSP group had higher mean baseline MoCA and ACE III total scores than CBS. On motor examination assessment, the PSP-cortical group had higher disability than the PSP-Richardson group; and the PSP-cortical group had higher disability than the PSP-subcortical group. On activities of daily living and quality of life markers, the PSP-cortical group had a higher burden of disease than the PSP-subcortical group. On neuropsychiatric assessment, the PSP-cortical group had a higher burden of disease than the PSP-subcortical group as measured by the CBI-R and ACE III. No differences were observed in serum NfL between groups. CSF T-tau was lower and CSF Aβ1-42 higher in PSP than CBS. The presence of an A-allele in the *LRRK2* genotype (rs2242367) was associated with a shorter disease duration at enrolment in the PSP group (3.5 ± 2.0 years versus 5.1 ± 3.4 years, *P* = 0.02). In the CBS group, the presence of an ε4 allele in the *APOE* genotype was associated with lower Aβ1-42 (377 ± 56 pg/ml versus 657 ± 314 pg/ml, *P* = 0.02) and higher T-tau (832 ± 120 pg/ml versus 404 ± 208 pg/ml, *P* < 0.001) values.

Symptom duration and survival data are displayed in Table 1. The PSP group was older at symptom onset (mean ± SD, 66.8 ± 7.15 years) than MSA (59.5 ± 10.08 years) and CBS (62.2 ± 7.70 years) although disease duration prior to baseline was similar (4.5 ± 2.92 years in PSP, 5.3 ± 3.01 years in CBS, 5.2 ± 2.71 years in MSA). The PSP-subcortical group had a longer diagnostic delay (6.3 ± 3.84 years) than the PSP-Richardson group (3.5 ± 2.16 years).

**Figure 2:**
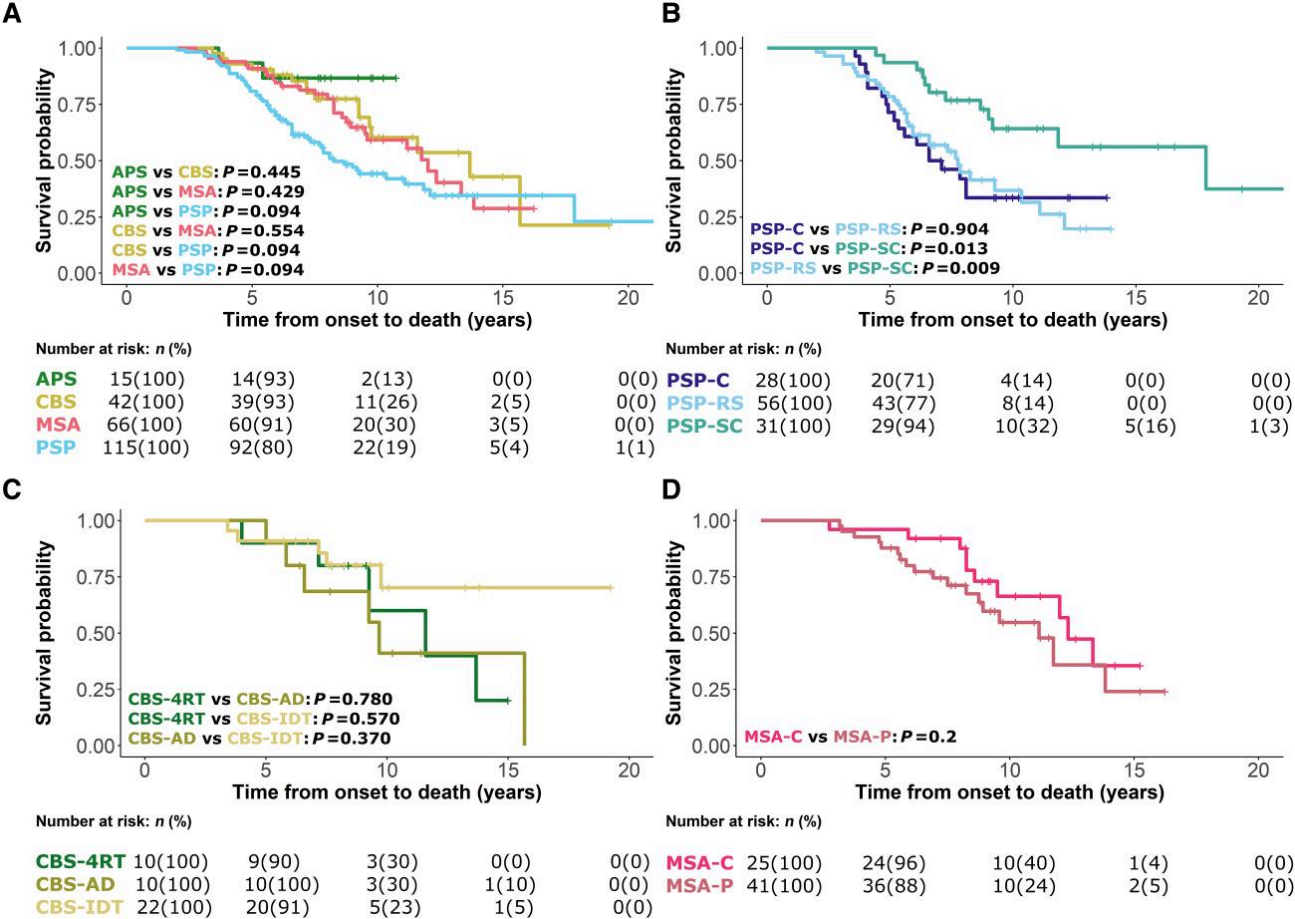
**Survival analysis from symptom onset to death**. Analysis was performed using a Cox regression model split according to diagnostic groups with associated number at risk tables below each plot. (**A**) Indeterminate (APS), corticobasal syndrome (CBS), multiple system atrophy (MSA) and progressive supranuclear palsy (PSP) groups. (**B**) PSP-cortical (PSP-C), PSP-subcortical (PSP-SC), and PSP-Richardson’s syndrome (PSP-RS) groups. (**C**) CBS-four-repeat tau (CBS-4RT), CBS-Alzheimer’s disease (CBS-AD) and CBS-indeterminate (CBS-IDT) groups. (**D**) MSA-cerebellar (MSA-C) and MSA-parkinsonism (MSA-P) groups.

One hundred and eight (108/243, 44%) participants had died by 48 months average follow-up, with a median (range) survival after baseline visit 2.2 (1.4–3.2) years and median disease duration 6.2 (4.8–8.3) years. Kaplan-Meier curves and log-rank comparisons for all patients from symptom onset to death according to disease group are presented in Fig. 2. PSP-subcortical phenotypes survived longer than the PSP-Richardson group (*P* = 0.009) and the PSP-cortical group (*P* = 0.013). No effects of age or sex on survival were observed across all analyses. Twenty-one (21/108, 19.4%) had pathological confirmation of diagnosis with high accuracy for those presenting with PSP and CBS, less so with MSA (Table 1).

### Missing data

Supplementary Table 2 provides the completion rate of each variable of interest at each assessment. Of those not completing 12 months follow-up, 15 (15/90, 17%) had died within 12 months of their baseline assessment and a further 10 (10/90, 11%) within 18 months. No significant differences in age, sex, disease phenotype or disease duration were observed between those completing 12 months follow-up versus those not completing 12 months follow-up. Disease severity was associated with follow-up completion; participants not completing 12 months follow-up were more severely affected across markers of functioning at baseline than those who completed 12 months follow-up.

### Rates of progression

Annualized progression data are given according to the final diagnosis group and phenotype, for clinical measures in Supplementary Table 3 and imaging measures in Supplementary Table 4. Faster rate of change of the CBI-R was observed in the PSP-cortical group versus the PSP-subcortical group. Imaging data, displayed in Supplementary Fig. 1, demonstrated faster volume loss in the pons of MSA versus IDT, CBS and PSP. Faster pons-midbrain ratio volume loss was observed in MSA versus PSP and CBS.

Annualized progression data are given according to the initial group and phenotype, for clinical measures in Supplementary Table 5 and imaging measures in Supplementary Table 6. No differences were observed in the progression of questionnaire scores or clinical data. Imaging data demonstrated faster volume loss in the pons of MSA versus IDT, CBS and PSP. Faster pons-midbrain ratio volume loss was observed in MSA versus PSP and CBS. No genotype/haplotype trends were observed.

### Sample size calculation for clinical trials

**Figure 3:**
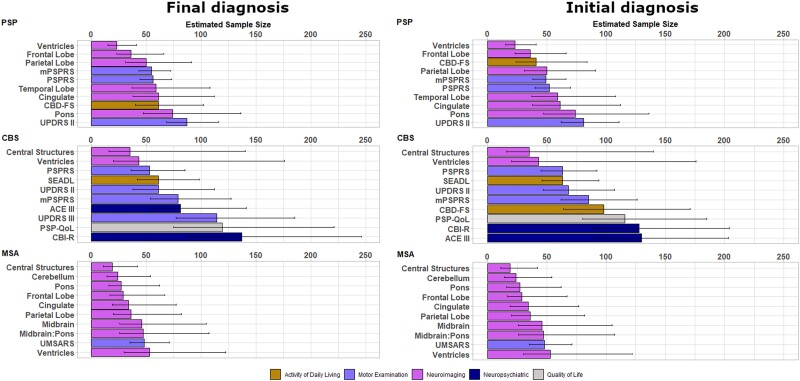
**Estimated sample size and bootstrapped confidence interval plots by final diagnosis at group level (*left*) and by intention to treat at group level (*right*)**. ACE III = Addenbrookes Cognitive Examination III; CBD-FS = Corticobasal Degeneration Functional Scale; CBI-R = Cambridge Behavioural Inventory Revised; CBS = corticobasal syndrome; mPSPRS = modified Progressive Supranuclear Palsy Rating Scale; MSA = multiple system atrophy; PSP = progressive supranuclear palsy; PSP QoL = PSP Quality of Life Scale; PSPRS = PSP rating scale; SEADL = Schwab and England Activities of Daily Living Scale; UMSARS = Unified MSA Rating Scale; UPDRS = Unified Parkinson’s Disease Rating Scale.

**Figure 4:**
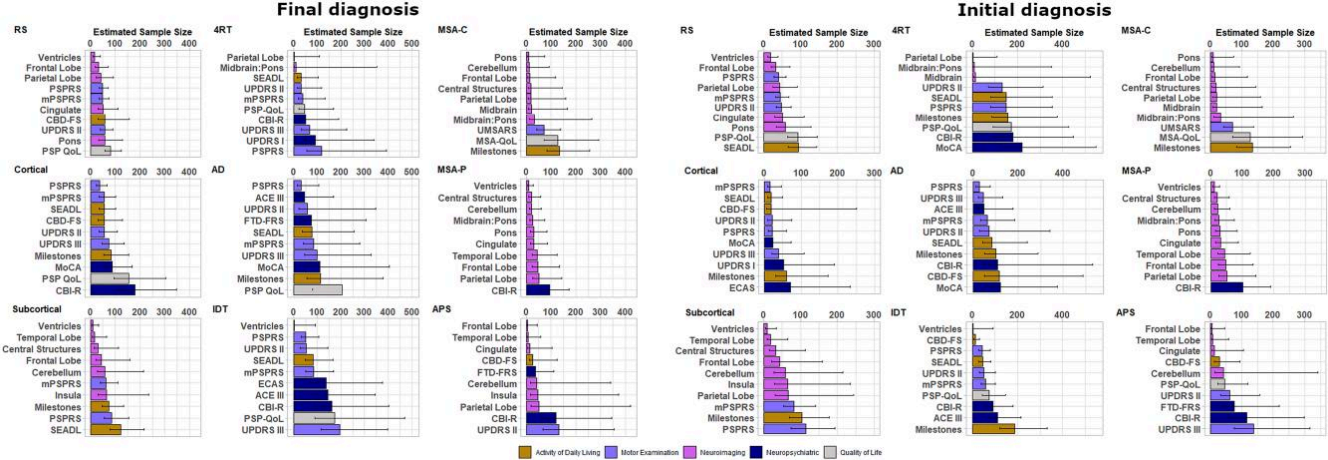
**Estimated sample size and bootstrapped confidence interval plots by final diagnosis and phenotype (*left*); and by intention to treat and phenotype (*right*)**. 4RT = 4-repeat tau; AD = Alzheimer disease; ACE III = Addenbrookes Cognitive Examination III; CBD-FS = Corticobasal Degeneration Functional Scale; CBI-R = Cambridge Behavioural Inventory Revised; CBS = corticobasal syndrome; ECAS = Edinburgh Cognitive and Behavioural Screen; FTD-FRS = Frontotemporal Dementia Rating Scale; IDT = indeterminate; MoCA = Montreal Cognitive Assessment; mPSPRS = modified Progressive Supranuclear Palsy Rating Scale; MSA = multiple system atrophy; MSA QoL = MSA Quality of Life Scale; PSP = progressive supranuclear palsy; PSP QoL = PSP Quality of Life Scale; PSPRS = PSP Rating Scale; SD = standard deviation; SEADL = Schwab and England Activities of Daily Living Scale; UPDRS = Unified Parkinson’s Disease Rating Scale; UMSARS = Unified MSA Rating Scale.

Supplementary Tables 7–10 show sample size calculations for candidate measures of activities of daily living, quality of life, motor examination, neuropsychiatric and neuroimaging measures according to both final diagnosis and initial diagnosis. We present these sample sizes for each main disease group, for a two-arm (drug versus placebo), 1-year trial, with 25% dropout in both arms, to achieve 80% power. We present sample sizes required to detect both a reduction in the rate of decline of 25% and 50%. Comprehensive sample size estimations are displayed in Supplementary Tables 11–18 for phenotypic subtypes and Supplementary Tables 21–24 for trial eligible subtypes. For each group, the ‘top 10’ most sensitive measures are presented with bootstrapped 95% confidence intervals (CI) in Figs 3 and 4, for the detection of a 50% reduction in rate of decline.

In PSP, defined by final diagnosis, three imaging measures had sample sizes ≤50 and a further four had sample sizes of ≤75. In the MSA group, eight imaging measures had sample sizes of ≤50. In the CBS group, four clinical measures had sample sizes ≤100, including the PSPRS, SEADL, UPDRS Part II and modified PSPRS (mPSPRS). The leading clinical measures in PSP were the mPSPRS and PSPRS, in CBS were the PSPRS and UPDRS Part II, and in MSA was the UMSARS. Activity of daily living, quality of life and neuropsychiatric measures were overall less sensitive than neuroimaging and motor examination measures.

In PSP, defined by baseline diagnosis, three imaging measures had sample sizes ≤50 and a further four had sample sizes of ≤75. In the MSA group, six imaging measures had sample sizes of ≤50. In the CBS group, five clinical measures had sample sizes ≤100, including the Corticobasal Degeneration Functional Scale (CBD-FS), SEADL, UPDRS Part II, PSPRS and mPSPRS. The leading clinical measures in PSP were the mPSPRS and PSPRS, in CBS were the PSPRs and UPDRS Part II, and in MSA was the UMSARS. Activity of daily living, quality of life and neuropsychiatric measures were again less sensitive than neuroimaging and motor examination measures.

Thirty-five of the PSP group (defined by baseline diagnosis, 35/52 = 67%) were deemed to have met trial eligibility criteria. In this subgroup, seven imaging measures and three clinical measures had sample sizes ≤50, with leading measures including central structures, midbrain and pons. The only markers with sample sizes ≤100 when utilizing 6-month data alone were the UPDRS Part II and PSPRS (Supplementary Fig. 3 and Supplementary Table 21).

Twenty-nine of the MSA group (defined by baseline diagnosis, 29/64 = 45%) were deemed to have met trial eligibility criteria. In this group, seven imaging measures had sample sizes ≤50, with leading measures including ventricles, cerebellum and central structures, and two other measures had sample sizes ≤100 (UMSARS and SEADL). The only marker with sample sizes ≤100 when utilizing 6-month data alone was the SEADL (Supplementary Fig. 3 and Supplementary Table 22).

## Discussion

The principal outcome of this study is the evidence base to inform selection of outcomes for disease-modifying clinical trials of PSP, CBS and MSA. The sensitivity to annual progression varies markedly between widely used scales of quality of life, activity of daily living, motor examination, neuropsychiatric and neuroimaging features, whether participants are defined by their final diagnosis or the initial diagnosis. There is marked heterogeneity of progression between disease groups and between phenotypes within each disease, indicating the need to consider a customized approach to clinical trials design. The common motor examination and activity of daily living measures performed well in PSP and CBS (samples sizes <100 per trial arm to detect a 50% slowing in progression), but neuroimaging measures were generally more sensitive (achieving required samples sizes <50 per trial arm to detect a 50% slowing in progression). We did not find evidence of significant non-linear (accelerating) progression, but this possibility is not excluded by our study given the limited sample size and 1-year observation window.

The PROSPECT-M-UK cohort of PSP-Richardson had similar demographics, baseline variable scores, rates of progression and consequent clinical trial sample size calculations as recent large observational studies.^10,20,21,34,39^ The PSP-subcortical group had the longest survival, with diagnostic delay from symptom onset and milder disease at recruitment across clinical, cognitive and functional variables. The PSP-cortical group had the worst survival, highest baseline variable scores and fastest rates of progression in the PSP spectrum. Our analysis supports the use of motor examination markers such as the full and mPSPRS and activity of daily living markers such as the CBD-FS in clinical trials, enabling sample sizes of <100 across all phenotypes. Other quality of life and neuropsychiatric markers performed less well over follow-up in PSP. Neuroimaging markers of progression showed superior sensitivity to progression of PSP and its subtypes, with similar rates of progression to previous reports.^15,42^

For CBS, CSF biomarkers or amyloid-PET imaging suggested the presence of AD pathology in many patients (*cf*. Jabbari *et al*.^8^ and Alexander *et al*.^36^). Neuropsychiatric markers of progression (e.g. ACE III) were more sensitive in CBS-AD, while activities of daily living (e.g. SEADL) and quality of life (e.g. PSP-QoL) markers were more sensitive in CBS-4RT. Although selective imaging markers appeared to offer small sample size needs, their wide confidence intervals may limit their use as outcome variables for clinical trials of undifferentiated CBS.

Sensitivity to change of progression markers in MSA varied by phenotype. The UMSARS progression was similar to previous studies with longer follow-up^16,43–45^ and produced sample sizes of <100 per arm to detect a 50% change in both MSA-C and MSA-P. The MSA-QoL performed better in MSA-C than MSA-P. Imaging markers performed strongly in terms of sample size calculations and could offer alternative end points for early phase clinical trials, particularly measures of subcortical structures.

The PROSPECT-M-UK also recruited people with indeterminate disorders that lay within the spectrum of parkinsonism other than idiopathic PD. They had features that in the investigators’ opinions were in keeping with potential variants of PSP, CBS or MSA but which fell outside consensus diagnostic criteria or had an intermediate phenotype of uncertain classification. Two-thirds of this indeterminate group progressed such that they later met diagnostic criteria for PSP, MSA or CBS. The indeterminate group had distinct characteristics: longer survival and more benign markers of disease severity at baseline. However, they had similar rates of progression on most clinical and imaging measures, perhaps in keeping with the conversion to standard phenotypes. Their heterogeneous progression undermined the sensitivity of clinical measures (requiring higher sample sizes) although cortical neuroimaging markers remained sensitive. Further follow-up and neuropathological examination may lead to a better understanding of the aetiology of the indeterminate group.

Clinical trials are typically modelled using an intention to treat approach where a working diagnosis and supportive investigations are available. The alternative approach, using a final best diagnosis, has the benefits of time, observation and sometimes neuropathological confirmation. In our cohort, 6% of participants had changed diagnosis after 1 year. Future disease-specific biomarkers may help improve baseline accuracy and reduce the rate of diagnostic revision mid-trial. We modelled both approaches, noting that sample size markers differ only slightly between the two approaches. Similar patterns were observed with neuroimaging markers performing strongly in PSP and MSA, motor examination markers performing strongly in PSP, and non-neuroimaging markers performing better in CBS. Current *in vivo* biomarkers do not match the gold standard of neuropathological confirmation, nor does any marker perform consistently well across all disorders. However, our results are reassuring that evolution of symptoms and diagnostic categorization over time is unlikely to require changes in clinical trial recruitment strategies.

The advantages of this study include the multicentre design and head-to-head comparison of candidate end points over the range of ‘atypical parkinsonian syndromes’ including PSP, MSA, CBS and IDT groups. However, there are limitations. Clinical criteria were used for diagnosis, and few had neuropathological confirmation. CBS-AD and CBS-4RT case distinction was performed using CSF biomarker criteria and/or amyloid-PET imaging status and we acknowledge that these surrogate markers do not prove that AD pathological features were the primary drivers of clinical symptoms since AD biomarker status may be coincidental and comorbid with CBD. Indeed, in the probable CBS-4RT group, the one patient who has so far come to post-mortem had AD pathology, demonstrating the limitations of these markers. In addition, longitudinal fluidic biomarker data were not available, and follow-up was incomplete. The rate of attrition by death or advanced disease, remains challenging to clinical trials but is in keeping with previous longitudinal studies of MSA, PSP and CBS. To mitigate these effects, we imputed data where participants did not complete up to 20% of the full assessment. Our analyses included symptom duration, which is necessarily only approximate, given the insidious nature of some early clinical features like personality and cognitive change. The disease process is likely to have begun many years earlier.^46–49^ The interpretation of symptom duration is also conditional on survival, which is not knowable at the time of trial entry. Despite these limitations, symptom duration is widely used in clinical settings and in several recent clinical trials criteria. We recognize that clinical trial methodology and stringent enrolment criteria may lead to exclusion of many people with PSP, CBS and MSA, which may influence progression rates and thereby size calculations.^10^ New trials may benefit from greater inclusivity of the full spectrum of disease phenotypes, with consideration of adjusted risk-benefit analyses and alternative outcome measures. The wide confidence intervals in some sample size estimates call for caution when comparing across measures and disease groups. New trials may also wish to focus on earlier stages of PSP, CBS and MSA, in anticipation of greater long-term gains from earlier intervention. Such an approach implies either the exclusion of a high proportion of patients (with an impact on generalizability of results), or a radical improvement in the delay from symptom onset to diagnosis. In the interim, we suggest that the types of patient included in this study are typical of those likely to be available for disease-modifying trials in the next few years.

In conclusion, we present the relative value of clinical, cognitive, functional and imaging markers of disease progression across the spectrum of atypical parkinsonian disorders (PSP, CBS, MSA, IDT). Future triallists can incorporate phenotypic variance and adjust the selection of optimal end points and sample sizes accordingly. These results of PROSPECT-M-UK will assist in modelling and planning future trials to ensure maximum benefit to the people affected by this devastating group of diseases.

## Supplementary Material

awad105_Supplementary_Data
