# Supplementary material for: Progression of atypical parkinsonian syndromes: PROSPECT-M-UK study implications for clinical trials
Source: Brain. Author manuscript; Available in PMC 2023 Aug 2. (PMC10393398; doi:10.1093/brain/awad105)
Supplement: Supplementary material [file EMS173240-supplement-Supplementary_material.docx]

**Supplementary Material Contents**

**Supplementary Figure 1 Page 3**

**Supplementary Figure 2 Page 4**

**Supplementary Figure 3 Page 5**

**Supplementary Table 1 Page 6**

**Supplementary Table 2 Page 7**

**Supplementary Table 3 Page 8**

**Supplementary Table 4 Page 9**

**Supplementary Table 5 Page 10**

**Supplementary Table 6 Page 11**

**Supplementary Table 7 Page 12**

**Supplementary Table 8 Page 13**

**Supplementary Table 9 Page 14**

**Supplementary Table 10 Page 15**

**Supplementary Table 11 Page 16**

**Supplementary Table 12 Page 17**

**Supplementary Table 13 Page 18**

**Supplementary Table 14 Page 19**

**Supplementary Table 15 Page 20**

**Supplementary Table 16 Page 21**

**Supplementary Table 17 Page 22**

**Supplementary Table 18 Page 23**

**Supplementary Table 19 Page 24-25**

**Supplementary Table 20 Page 26-28**

**Supplementary Table 21 Page 29**

**Supplementary Table 22 Page 30**

**Supplementary Table 23 Page 31**

**Supplementary Table 24 Page 32**

**Supplementary Methods Page 33**

**Supplementary References Page 34**


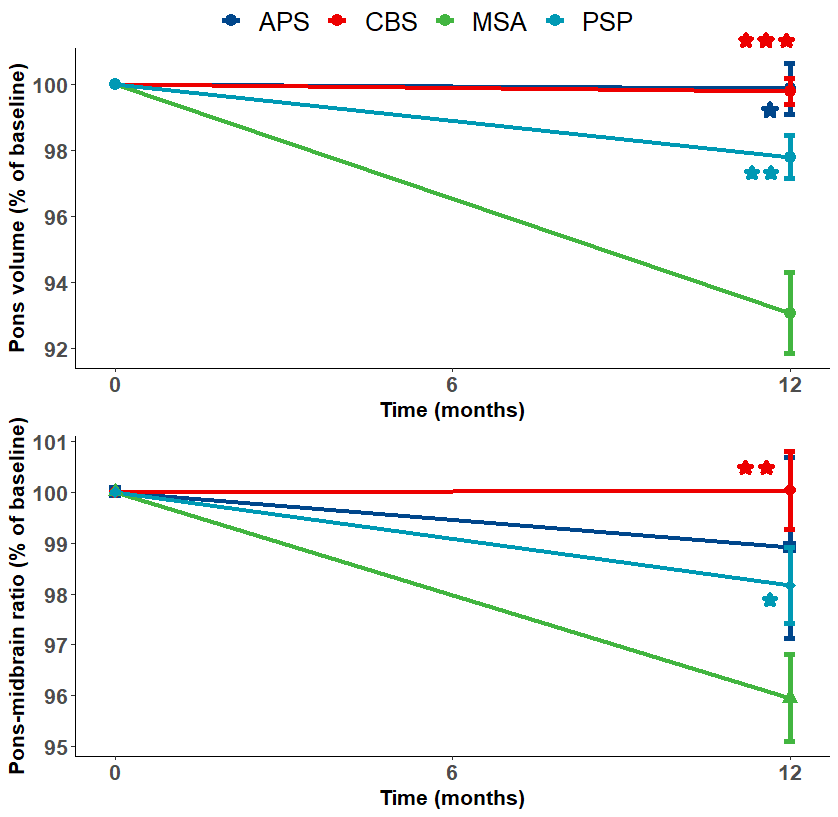


**Supplementary Figure 1: Longitudinal changes in brain volume.** Mean values at baseline and 12 months for indeterminate (APS), corticobasal syndrome (CBS), multiple system atrophy (MSA) and progressive supranuclear palsy (PSP) groups. Significance change difference is presented relative to MSA over 0-12 months by *p<0.05, **p<0.01 and ***p<0.001.


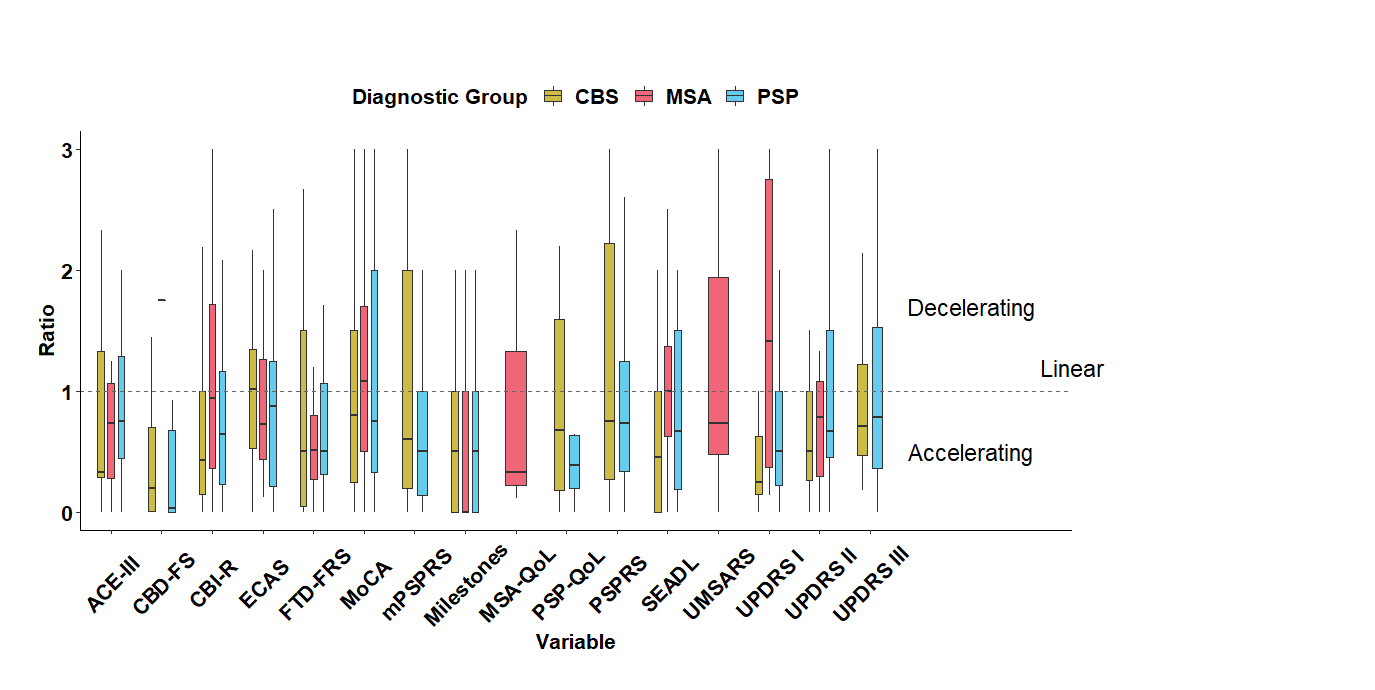


**Supplementary Figure 2: Ratios of rate of biomarker change between 0-6 months and 6-12 months split according to diagnostic group.** Abbreviations: ACE III = Addenbrookes Cognitive Examination III, CBD-FS = Corticobasal Degeneration Functional Scale, CBI-R = Cambridge Behavioural Inventory Revised, CBS = corticobasal syndrome , ECAS = Edinburgh Cognitive and Behavioural Screen, FTD-FRS = Frontotemporal Dementia Rating Scale, MoCA = Montreal Cognitive Assessment, mPSPRS = modified Progressive Supranuclear Palsy Rating Scale, MSA = multiple system atrophy, MSA-QoL = MSA Quality of life scale, PSP = progressive supranuclear palsy, PSP-QoL = PSP Quality of life scale, PSPRS = PSP rating scale, SEADL = Schwab and England Activities of Daily Living Scale, UPDRS = Unified Parkinson’s Disease Rating Scale, UMSARS = Unified MSA Rating Scale.


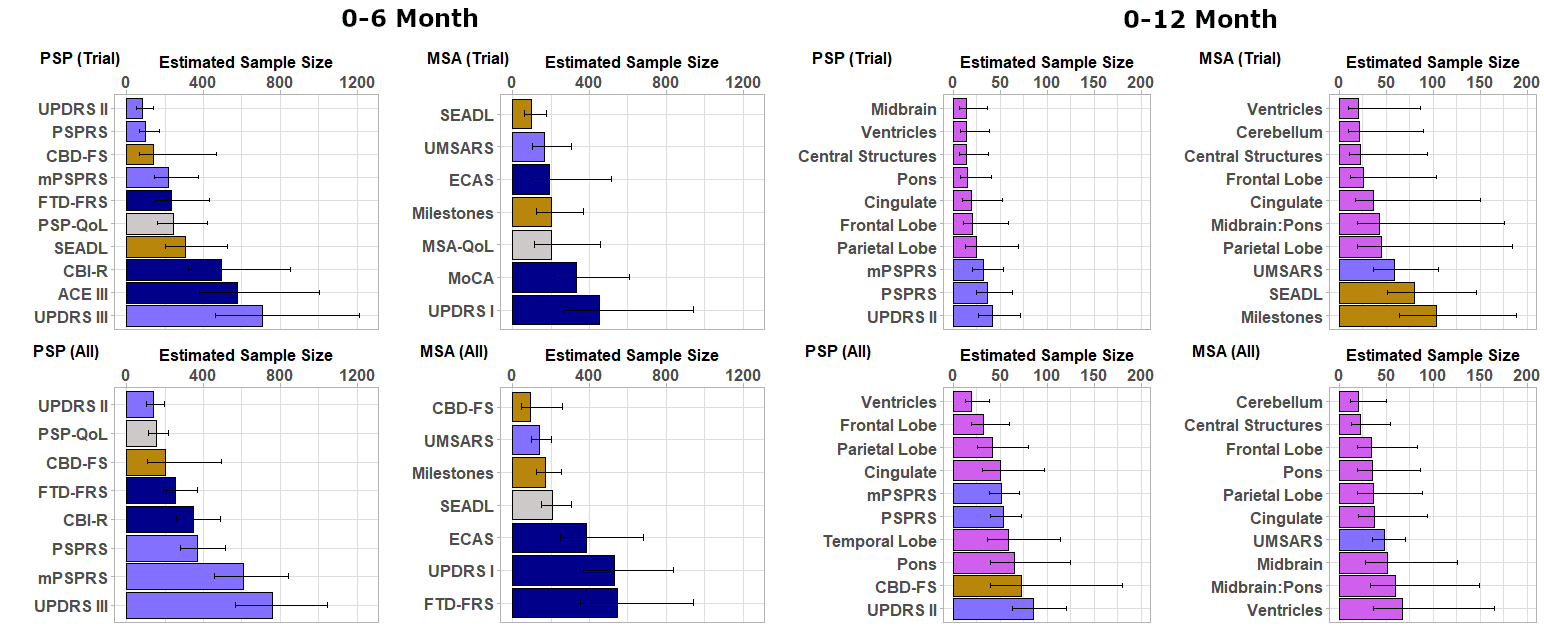


**Supplementary Figure 3: Estimated sample size and bootstrapped confidence interval plots by six- (left); and twelve-month (right) progression in trial eligible groups** (N.B. Excluded results with estimated sample sizes >1000). Abbreviations: ACE III = Addenbrookes Cognitive Examination III, CBD-FS = Corticobasal Degeneration Functional Scale, CBI-R = Cambridge Behavioural Inventory Revised, ECAS = Edinburgh Cognitive and Behavioural Screen, FTD-FRS = Frontotemporal Dementia Rating Scale, MoCA = Montreal Cognitive Assessment, mPSPRS = modified Progressive Supranuclear Palsy Rating Scale, MSA = multiple system atrophy, MSA-QoL = MSA Quality of life scale, PSP = progressive supranuclear palsy, PSP-QoL = PSP Quality of life scale, PSPRS = PSP rating scale, SEADL = Schwab and England Activities of Daily Living Scale, UPDRS = Unified Parkinson’s Disease Rating Scale, UMSARS = Unified MSA Rating Scale.

**Supplementary Table 1: Baseline values of Activity of Daily Living, Quality of Life, Motor Examination, Neuropsychiatric and fluid markers by Final diagnosis**

|  | **PSP** | | | | **CBS** | | | | **MSA** | | | **IDT** |  |
| --- | --- | --- | --- | --- | --- | --- | --- | --- | --- | --- | --- | --- | --- |
|  | **All**  **(n=117)** | **RS**  **(n=57)** | **Cortical**  **(n=28)** | **Subcortical**  **(n=32)** | **All**  **(n=42)** | **4RT**  **(n=10)** | **AD**  **(n=10)** | **Unknown**  **(n=22)** | **All**  **(n=68)** | **Parkinsonism**  **(n=41)** | **Cerebellar**  **(n=26)** | **(n=16)** |  |
| **CBD-FS** | 41·3 (19·1) | 33·2 (18·7) | 49·8 (19·4) | 36·0 (10·6) | 36·7 (22·2) | 45·2 (19·1) | 40·0 (27·8) | 21·8 (9·2) | ·· | ·· | ·· | 32·3 (11·6) |  |
| **SEADL** | 54·3 (21·9) | 57·0 (22·6) | 41·8 (20·6)^r^ | 60·6 (17·7) | 53·3 (21·7)^b^ | 48·0 (21·5) | 55·0 (21·2) | 55·0 (22·6) | 57·2 (24·2)^b^ | 56·6 (25·7) | 58·1 (22·1) | 73·3 (18·0)^d^ |  |
| **Milestones** | 3·5 (1·6)^i^ | 3·7 (1·5) | 4·0 (1·6) | 2·8 (1·6) | 1·9 (2·0) | 2·5 (2·8) | 1·2 (1·6) | 1·9 (1·7) | 2·0 (1·4) | 1·8 (1·5) | 2·2 (1·3) | 1·8 (1·3)^c,k^ |  |
| **MSA QoL** | ·· | ·· | ·· | ·· | ·· | ·· | ·· | ·· | 43·5 (16·6) | 44·5 (18·1) | 42·3 (15·0) | ·· |  |
| **PSP QoL** | 63·0 (32·8) | 58·7 (31·7)^o^ | 84·5 (37·9)^q^ | 53·6 (22·3) | 52·7 (28·6) | 65·3 (32·1) | 42·8 (15·8) | 48·5 (29·3) | ·· | ·· | ·· | 48·8 (25·6) |  |
| **UPDRS II** | 23·5 (9·5) | 20·2 (8·4)^n^ | 29·2 (11·1) | 24·1 (7·4) | 20·2 (10·2) | 25·8 (11·5) | 16·2 (6·4) | 18·3 (9·6) | 23·6 (9·6)^b^ | 23·6 (9·4) | 23·6 (10·2) | 15·9 (7·3)^d^ |  |
| **UPDRS III** | 44·0 (17·3) | 38·9 (15·1)^n^ | 56·0 (20·8) | 42·4 (12·2) | 49·3 (22·7)^a,h^ | 53·4 (20·4) | 48·8 (21·7) | 47·6 (24·7) | ·· | ·· | ·· | 30·1 (16·0)^d^ |  |
| **PSPRS** | 33·0 (13·0) | 31·3 (11·6)^o^ | 42·4 (13·7)^p^ | 27·6 (10·5) | 30·2 (14·6)^b^ | 37·9 (15·4) | 29·3 (16·7) | 27·0 (12·4) | ·· | ·· | ·· | 19·5 (12·1)^c,m^ |  |
| **mPSPRS** | 13·3 (4·2) | 12·9 (4·1) | 15·3 (4·3) | 12·3 (3·8) | 10·3 (5·4) | 12·6 (4·7) | 10·1 (5·6) | 9·3 (5·5) | ·· | ·· | ·· | 7·9 (4·6) |  |
| **UMSARS** | ·· | ·· | ·· | ·· | ·· | ·· | ·· | ·· | 45·0 (15·2) | 43·4 (15·8) | 47·2 (14·2) | ·· |  |
| **UPDRS I** | 9·6 (4·5) | 9·2 (4·0) | 11·5 (5·6) | 8·6 (3·8) | 8·3 (5·2)^f^ | 11·6 (5·5)^s^ | 3·3 (1·2) | 8·2 (4·6) | 13·1 (5·0) | 13·9 (4·6) | 12·0 (5·5) | 11·5 (3·9)^l^ |  |
| **CBI-R** | 46·3 (30·9) | 46·5 (30·1) | 60·4 (37·4)^q^ | 34·1 (20·3) | 40·5 (27·4) | 53·0 (29·7) | 54·7 (32·2) | 27·4 (17·5) | 34·7 (20·7) | 32·9 (18·6) | 37·3 (23·6) | 24·8 (11·3) |  |
| **MoCA** | 22·2 (4·8)^j^ | 22·3 (4·0) | 20·5 (5·7) | 23·5 (5·1) | 19·7 (7·9)^e^ | 21·5 (8·4) | 14·2 (8·5)^t^ | 21·3 (6·3) | 25·3 (3·2) | 25·4 (3·4) | 25·0 (3·1) | 22·5 (5·1)^m^ |  |
| **ACE III** | 78·8 (13·3)^j^ | 79·2 (10·7) | 71·0 (18·1)^r^ | 84·2 (10·9) | 71·4 (22·8)^e^ | 80·9 (17·5) | 60·4 (29·1) | 72·8 (19·9) | 87·2 (7·7) | 87·8 (7·5) | 86·3 (8·0) | 81·7 (11·8)^m^ |  |
| **ECAS** | 87·2 (20·9) | 87·2 (16·5) | 77·6 (26·4) | 94·7 (20·8) | 80·0 (31·7)^g^ | 88·0 (28·7) | 57·0 (36·2)^t^ | 89·2 (24·6) | 99·4 (12·7) | 99·5 (13·8) | 99·4 (11·2) | 91·1 (20·3) |  |
| **FTD-FRS** | 19·0 (10·4) | 18·6 (10·8) | 21·2 (10·2) | 18·1 (9·9) | 17·3 (12·1) | 19·6 (12·7) | 21·4 (11·5) | 14·3 (12·0) | 15·9 (10·0) | 15·6 (9·6) | 16·1 (10·6) | 12·9 (10·0) |  |
| **Serum NF-L level** | 47·1 (13·1) | 45.3 (27.4) | 60.1 (41.4) | 40.1 (21.0) | 51·1 (29·6) | 52.5 (26.5) | 36.5 (20.5) | 55.9 (33.1) | 32·6 (12·4) | 32.6 (12.4) | ·· | 29·5 (19·7) |  |
| **CSF Aβ1-42 level** | 824 (316) | 1124 (235) | 768 (320) | 631 (17.8) | 572 (263) | 748 (223) | 351 (69.4) | ·· | ·· | ·· | ·· | ·· |  |
| **CSF T-tau level** | 284 (79·1) | 300 (111) | 274 (79.3) | 297 (44.9) | 499 (272) | 277 (76.0) | 777 (119) | ·· | ·· | ·· | ·· | ·· |  |
| **CSF T-tau: Aβ1-42 ratio** | 0·4 (0·1) | 0·3 (0·1) | 0·4 (0·2) | 0·5 (0·1) | 1·2 (1·0) | 0·4 (0·1) | 2·3 (0·5) | ·· | ·· | ·· | ·· | ·· |  |
| a. Tukey post hoc test (TPH) adjusted *p* < 0·001 vs APS-all; b. TPH adjusted *p* < 0·05 vs APS-all; c. TPH adjusted *p* < 0·01 vs PSP-all; d. TPH adjusted *p* < 0·05 vs PSP-all; e. TPH adjusted *p* < 0·0001 vs MSA-all; f. TPH adjusted *p* < 0·001 vs MSA-all; g. TPH adjusted *p* < 0·01 vs MSA-all; h. TPH adjusted *p* < 0·05 vs MSA-all; i. TPH adjusted *p* < 0·0001 vs CBS-all; j. TPH adjusted *p* < 0·05 vs CBS-all; k. TPH adjusted *p* < 0·0001 vs PSP-all; l. TPH adjusted *p* < 0·001 vs PSP-all; m. TPH adjusted *p* < 0·05 vs PSP-all; n. TPH adjusted *p* < 0·01 vs PSP-cortical; o. TPH adjusted *p* < 0·05 vs PSP-cortical; p. TPH adjusted *p* < 0·001 vs PSP-subcortical; q. TPH adjusted *p* < 0·01 vs PSP-subcortical; r. TPH adjusted *p* < 0·05 vs PSP-subcortical; s. TPH adjusted *p* < 0·05 vs CBS-AD; t. TPH adjusted *p* < 0·05 vs CBS-IDT. Abbreviations: 4RT = 4-repeat tau, Aβ1-42 **=** β-amyloid 1-42, AD = Alzheimer disease, ACE III = Addenbrookes Cognitive Examination III, CBD-FS = Corticobasal Degeneration Functional Scale, CBI-R = Cambridge Behavioural Inventory Revised, CBS = corticobasal syndrome , ECAS = Edinburgh Cognitive and Behavioural Screen, FTD-FRS = Frontotemporal Dementia Rating Scale, IDT = Indeterminate, MoCA = Montreal Cognitive Assessment, mPSPRS = modified Progressive Supranuclear Palsy Rating Scale, MSA = multiple system atrophy, MSA QoL = MSA Quality of life scale, NF-L = neurofilament light chain, PSP = progressive supranuclear palsy, PSP QoL = PSP Quality of life scale, PSPRS = PSP rating scale, SEADL = Schwab and England Activities of Daily Living Scale, UPDRS = Unified Parkinson’s Disease Rating Scale, UMSARS = Unified MSA Rating Scale | | | | | | | | | | | | | |

**Supplementary Table 2: Completion rate of each variable at each assessment**

|  | **Baseline (raw)** | **Baseline**  **(% of whole)** | **Six months (raw)** | **Six months**  **(% of whole)** | **Twelve months (raw)** | **Twelve months**  **(% of whole)** | **Twelve months as % of baseline** | **Less than twelve months follow-up mean (SD) baseline score** | **Twelve months follow-up mean (SD) baseline score** | **p-value** |
| --- | --- | --- | --- | --- | --- | --- | --- | --- | --- | --- |
|  |  |  |  |  |  |  |  |  |  |  |
| **CBD-FS** | 76 | 31 | 57 | 23 | 76 | 31 | 100 | 42·8 (19·6) | 35·8 (18·2) | 0·14 |
| **SEADL** | 239 | 98 | 141 | 58 | 130 | 53 | 54 | 49·4 (23·4) | 60·0 (21·4) | 0·00060 |
| **Milestones** | 235 | 97 | 137 | 56 | 131 | 54 | 56 | 2·8 (1·8) | 2·7 (1·8) | 0·77 |
|  |  |  |  |  |  |  |  |  |  |  |
| **MSA-QoL** | 38 | 56 | 22 | 32 | 13 | 19 | 34 | 42·6 (21·3) | 44·2 (12·7) | 0·79 |
| **PSP-QoL** | 154 | 63 | 86 | 35 | 79 | 33 | 51 | 72·0 (32·1) | 55·8 (30·9) | 0·0067 |
|  |  |  |  |  |  |  |  |  |  |  |
| **UPDRS – Part 2** | 198 | 81 | 116 | 48 | 103 | 42 | 52 | 25·2 (10·2) | 21·4 (9·2) | 0·028 |
| **UPDRS – Part 3** | 179 | 74 | 111 | 46 | 97 | 40 | 54 | 50·2 (19·6) | 40·4 (18·3) | 0·0012 |
| **PSPRS** | 179 | 74 | 111 | 46 | 98 | 40 | 55 | 35·3 (14·3) | 28·4 (13·1) | 0·0028 |
| **UMSARS** | 60 | 88 | 33 | 49 | 30 | 44 | 50 | 44·5 (15·9) | 45·4 (14·6) | 0·81 |
|  |  |  |  |  |  |  |  |  |  |  |
| **UPDRS – Part 1** | 198 | 81 | 116 | 48 | 103 | 42 | 52 | 11·7 (5·0) | 9·8 (4·9) | 0·024 |
| **CBI-R** | 199 | 82 | 119 | 49 | 100 | 41 | 50 | 45·6 (28·8) | 39·1 (27·2) | 0·11 |
| **MoCA** | 224 | 92 | 131 | 54 | 128 | 53 | 57 | 21·1 (6·2) | 23·4 (4·8) | 0·0036 |
| **ACE III** | 207 | 85 | 123 | 51 | 119 | 49 | 57 | 75·0 (17·6) | 82·6 (12·7) | 0·00043 |
| **ECAS** | 174 | 72 | 115 | 47 | 89 | 37 | 51 | 82·9 (22·6) | 91·4 (21·9) | 0·0096 |
| **FTD-FRS** | 175 | 72 | 103 | 42 | 88 | 36 | 50 | 18·9 (10·9) | 17·0 (10·5) | 0·22 |
|  |  |  |  |  |  |  |  |  |  |  |
| **Blood** | 145 | 60 | ·· | ·· | ·· | ·· | ·· | ·· | ·· | ·· |
| **CSF** | 36 | 15 | ·· | ·· | ·· | ·· | ·· | ·· | ·· | ·· |
| **Genetics** | 148 | 61 | ·· | ·· | ·· | ·· | ·· | ·· | ·· | ·· |
| **MRI** | 104 | 43 | ·· | ·· | 59 | 24 | 48 | ·· | ·· | ·· |
| Abbreviations: 4RT = 4-repeat tau, AD = Alzheimer disease, ACE III = Addenbrookes Cognitive Examination III, CBD-FS = Corticobasal Degeneration Functional Scale, CBI-R = Cambridge Behavioural Inventory Revised, CBS = corticobasal syndrome , ECAS = Edinburgh Cognitive and Behavioural Screen, FTD-FRS = Frontotemporal Dementia Rating Scale, IDT = Indeterminate, MoCA = Montreal Cognitive Assessment, mPSPRS = modified Progressive Supranuclear Palsy Rating Scale, MSA = multiple system atrophy, MSA QoL = MSA Quality of life scale, PSP = progressive supranuclear palsy, PSP QoL = PSP Quality of life scale, PSPRS = PSP rating scale, SD = standard deviation, SEADL = Schwab and England Activities of Daily Living Scale, UPDRS = Unified Parkinson’s Disease Rating Scale, UMSARS = Unified MSA Rating Scale | | | | | | | | | | |

**Supplementary Table 3: Annualised rates of progression in Activity of Daily Living, Quality of Life, Motor Examination, and Neuropsychiatric markers by Final diagnosis**

|  | **PSP** | | | | **CBS** | | | | **MSA** | | | **IDT** |
| --- | --- | --- | --- | --- | --- | --- | --- | --- | --- | --- | --- | --- |
|  | **All**  **(n=117)** | **RS**  **(n=57)** | **Cortical**  **(n=28)** | **Subcortical**  **(n=32)** | **All**  **(n=42)** | **4RT**  **(n=10)** | **AD**  **(n=10)** | **Unknown**  **(n=22)** | **All**  **(n=68)** | **Parkinsonism**  **(n=41)** | **Cerebellar**  **(n=26)** | **(n=16)** |
| **CBD-FS** | 15·8 (10·0) | 14·2 (8·3) | 19·9 (13·8) | 14·1 (6·7) | 16·4 (7·6) | 16·3 (1·9) | 15·2 (14·8) | 17·2 (3·1) | ·· | ·· | ·· | 12·9 (8·8) |
| **SEADL** | -11·0 (15·5) | -11·1 (19·0) | -10·5 (8·7) | -11·2 (13·7) | -14·3 (10·7) | -16·5 (10·7) | -14·9 (13·9) | -13·0 (9·4) | -12·1 (11·3) | -10·5 (7·8) | -14·7 (15·2) | -9·1 (13·1) |
| **Milestones** | 1·1 (1·5) | 0·9 (1·6) | 1·2 (1·9) | 1·3 (1·1) | 0·7 (1·0) | 0·6 (1·5) | 0·7 (0·6) | 0·7 (0·9) | 0·9 (1·0) | 0·9 (1·1) | 0·9 (0·9) | 0·6 (1·1) |
| **MSA QoL** | ·· | ·· | ·· | ·· | ·· | ·· | ·· | ·· | 1·5 (11·3) | -0·02 (11·6) | 3·3 (10·9) | ·· |
| **PSP QoL** | 23·0 (20·7) | 23·3 (18·8) | 28·5 (24·3) | 18·0 (19·9) | 23·9 (22·6) | 37·0 (27·3) | 27·8 (18·6) | 14·5 (17·3) | ·· | ·· | ·· | 15·6 (16·8) |
| **UPDRS II** | 6·9 (6·2) | 7·4 (6·5) | 8·6 (5·5) | 4·6 (5·8) | 7·9 (6·4) | 9·6 (6·9) | 9·6 (7·7) | 6·2 (5·4) | 5·3 (6·1) | 5·6 (4·5) | 4·9 (7·9) | 3·7 (4·4) |
| **UPDRS III** | 9·3 (12·0) | 9·0 (12·9) | 10·9 (10·1) | 8·4 (12·0) | 9·0 (7·1) | 10·8 (7·8) | 10·4 (8·4) | 7·5 (6·1) | ·· | ·· | ·· | 4·9 (11·6) |
| **PSPRS** | 9·0 (10·1) | 9·7 (11·3) | 10·9 (9·3) | 6·3 (8·0) | 9·9 (7·3) | 10·3 (10·8) | 10·6 (5·5) | 9·4 (6·4) | ·· | ·· | ·· | 4·2 (7·1) |
| **mPSPRS** | 3·1 (2·8) | 3·2 (3·0) | 3·2 (2·9) | 2·6 (2·2) | 3·3 (2·4) | 3·6 (2·3) | 3·4 (2·7) | 3·2 (2·4) | ·· | ·· | ·· | 1·7 (2·2) |
| **UMSARS** | ·· | ·· | ·· | ·· | ·· | ·· | ·· | ·· | 10·6 (7·5) | 12·0 (7·0) | 8·6 (8·0) | ·· |
| **UPDRS I** | 1·2 (3·8) | 0·8 (4·2) | 2·4 (4·5) | 0·9 (2·3) | 1·6 (3·0) | 2·3 (3·6) | 3·3 (3·7) | 0·5 (1·9) | 0·5 (3·3) | 0·4 (3·2) | 0·6 (3·6) | 0·4 (2·8) |
| **CBI-R** | 12·7 (19·0) | 15·2 (19·2) | 18·1 (19·4)^a^ | 3·6 (15·5) | 15·2 (17·0) | 18·6 (14·4) | 19·7 (14·8) | 11·6 (19·0) | 11·3 (13·8) | 13·0 (12·8) | 8·9 (15·0) | 8·9 (11·2) |
| **MoCA** | -0·9 (3·7) | -0·8 (3·0) | -1·6 (3·9) | -0·6 (4·6) | -1·5 (2·8) | -1·0 (1·6) | -1·8 (2·6) | -1·6 (3·3) | -0·4 (2·6) | -0·7 (2·3) | 0·2 (2·9) | 0·2 (2·5) |
| **ACE III** | -1·7 (7·1) | -1·6 (4·0) | -3·9 (9·1) | -0·3 (9·3) | -4·0 (6·3) | -4·1 (5·9) | -5·8 (8·5) | -2·9 (5·1) | -1·2 (5·6) | -0·8 (6·0) | -1·8 (4·7) | -1·9 (3·1) |
| **ECAS** | -2·7 (12·3) | -4·7 (9·5) | -3·2 (6·5) | 1·4 (18·7) | -3·4 (7·5) | -1·5 (6·0) | -1·7 (7·4) | -5·7 (8·1) | -4·3 (8·8) | -3·7 (7·3) | -5·2 (11·1) | 2·9 (10·9) |
| **FTD-FRS** | 4·0 (8·2) | 5·0 (7·6) | 4·5 (7·9) | 1·7 (9·2) | 4·9 (10·6) | 6·0 (17·3) | 3·4 (5·0) | 5·1 (9·3) | 2·5 (5·3) | 3·5 (3·6) | 1·6 (6·5) | 3·5 (1·9) |
| a. Tukey post hoc test (TPH) adjusted *p* < 0·05 vs PSP-subcortical. Variables with fewer than 5 responses per group have been removed. Abbreviations: 4RT = 4-repeat tau, AD = Alzheimer disease, ACE III = Addenbrookes Cognitive Examination III, CBD-FS = Corticobasal Degeneration Functional Scale, CBI-R = Cambridge Behavioural Inventory Revised, CBS = corticobasal syndrome , ECAS = Edinburgh Cognitive and Behavioural Screen, FTD-FRS = Frontotemporal Dementia Rating Scale, IDT = Indeterminate, MoCA = Montreal Cognitive Assessment, mPSPRS = modified Progressive Supranuclear Palsy Rating Scale, MSA = multiple system atrophy, MSA QoL = MSA Quality of life scale, PSP = progressive supranuclear palsy, PSP QoL = PSP Quality of life scale, PSPRS = PSP rating scale, SEADL = Schwab and England Activities of Daily Living Scale, UPDRS = Unified Parkinson’s Disease Rating Scale, UMSARS = Unified MSA Rating Scale. | | | | | | | | | | | | |

**Supplementary Table 4: Annualised rates of progression in neuroimaging markers (percentage change from baseline) by Final diagnosis**

|  | **PSP** | | | | **CBS** | | | | **MSA** | | | **IDT** |
| --- | --- | --- | --- | --- | --- | --- | --- | --- | --- | --- | --- | --- |
|  | **All**  **(n=28)** | **RS**  **(n=19)** | **Cortical**  **(n=0)** | **Subcortical**  **(n=9)** | **All**  **(n=8)** | **4RT**  **(n=3)** | **AD**  **(n=2)** | **Unknown**  **(n=3)** | **All**  **(n=17)** | **Parkinsonism**  **(n=12)** | **Cerebellar**  **(n=5)** | **(n=5)** |
| **Temporal lobe** | -2·8 (2·3) | -2·6 (2·7) | ·· | -3·0 (1·3) | -1·9 (4·4) | 0·02 (0·4) | -4·6 (9·3) | -1·3 (1·8) | -3·2 (2·6) | -3·4 (2·4) | -2·4 (3·3) | -3·1 (1·6) |
| **Cingulate** | -2·0 (2·1) | -2·3 (2·4) | ·· | -1·4 (1·2) | -1·9 (2·9) | -1·5 (1·1) | -2·7 (6·6) | -1·5 (1·1) | -3·0 (2·7) | -2·6 (1·8) | -4·0 (4·2) | -2·6 (2·0) |
| **Insula** | -1·2 (2·5) | -0·9 (2·6) | ·· | -2·0 (2·1) | -1·0 (3·2) | 2·3 (1·9) | -2·5 (5·0) | -2·2 (1·3) | -2·3 (3·2) | -1·9 (2·4) | -3·3 (4·8) | -1·9 (1·9) |
| **Occipital lobe** | -2·5 (2·6) | -2·3 (2·7) | ·· | -2·9 (2·5) | -1·7 (4·7) | 1·6 (0·2) | -2·5 (9·8) | -3·4 (1·5) | -2·6 (3·3) | -3·0 (3·3) | -1·7 (3·5) | -3·0 (3·8) |
| **Frontal lobe** | -3·8 (3·1) | -4·0 (3·5) | ·· | -3·3 (2·1) | -2·1 (4·4) | -0·7 (0·06) | -2·2 (11·0) | -2·8 (0·8) | -4·4 (3·9) | -3·9 (3·4) | -5·5 (5·1) | -3·7 (1·7) |
| **Parietal lobe** | -3·0 (2·9) | -2·9 (3·0) | ·· | -3·1 (2·9) | -2·3 (6·3) | -0·4 (0·04) | -0·9 (14·0) | -4·5 (2·0) | -3·9 (3·4) | -4·0 (3·1) | -3·7 (4·4) | -2·6 (2·0) |
| **Central structures** | -1·9 (3·5) | -1·9 (4·2) | ·· | -2·1 (1·5) | -2·8 (2·2) | -0·7 (1·2) | -3·3 (0·5) | -3·9 (2·6) | -3·6 (3·0) | -3·1 (2·1) | -4·8 (4·6) | -1·0 (1·2) |
| **Ventricles** | 11·0 (6·7) | 11·8 (7·6) | ·· | 9·1 (4·1) | 13·4 (10·1) | 1·1 (2·9) | 22·0 (3·0) | 15·9 (8·1) | 15·9 (12·3) | 13·6 (6·3) | 21·3 (21·2) | 9·2 (6·7) |
| **Cerebellum** | -3·4 (4·1) | -3·4 (4·7) | ·· | -3·5 (2·5) | -1·9 (3·7) | -0·6 (2·0) | -5·5 (5·1) | -0·4 (2·7) | -5·6 (3·9) | -6·1 (3·5) | -4·4 (5·1) | -4·5 (4·0) |
| **Medulla** | 0·9 (7·1) | 0·4 (5·9) | ·· | 2·0 (9·7) | -0·3 (2·5) | -1·0 (3·3) | 2·5 (1·2) | -1·7 (1·3) | -0·6 (3·0) | -0·2 (3·4) | -1·4 (2·1) | 0·8 (1·8) |
| **Pons** | -2·2 (3·1)^b^ | -2·7 (3·5) | ·· | -1·0 (1·6) | -0·2 (1·1)^a^ | 0·4 (0·8) | 0·6 (0·1) | -1·2 (0·8) | -7·1 (4·5) | -6·9 (4·9) | -7·7 (4·0) | -0·3 (1·4)^c^ |
| **Midbrain** | -0·5 (4·9) | -1·0 (5·3) | ·· | 0·7 (3·9) | -0·2 (2·1) | -2·1 (1·8) | 2·2 (1·9) | -0·6 (0·7) | -2·8 (3·6) | -1·7 (2·2) | -5·5 (4·9) | 0·5 (4·5) |
| **Midbrain:Pons ratio** | 0·9 (7·1)^c^ | 0·4 (5·9) | ·· | 2·0 (9·7) | -0·3 (2·5)^b^ | -1·0 (3·3) | 2·5 (1·2) | -1·7 (1·3) | -0·6 (3·0) | -0·2 (3·4) | -1·4 (2·1) | 0·8 (1·8) |
| a. Tukey post hoc test (TPH) adjusted *p* < 0·001 vs MSA-all; b. TPH adjusted *p* < 0·01 vs MSA-all; c. TPH adjusted *p* < 0·05 vs MSA-all. Abbreviations: 4RT = 4-repeat tau, AD = Alzheimer disease, CBS = corticobasal syndrome, IDT = Indeterminate, MSA = multiple system atrophy, PSP = progressive supranuclear palsy | | | | | | | | | | | | |

**Supplementary Table 5: Annualised rates of progression in Activity of Daily Living, Quality of Life, Motor Examination, and Neuropsychiatric markers by Intention to Treat**

|  | **PSP** | | | | **CBS** | | | | **MSA** | | | **IDT** |
| --- | --- | --- | --- | --- | --- | --- | --- | --- | --- | --- | --- | --- |
|  | **All**  **(n=99)** | **RS**  **(n=52)** | **Cortical**  **(n=12)** | **Subcortical**  **(n=35)** | **All**  **(n=60)** | **4RT**  **(n=16)** | **AD**  **(n=12)** | **Unknown**  **(n=32)** | **All**  **(n=64)** | **Parkinsonism**  **(n=38)** | **Cerebellar**  **(n=26)** | **(n=20)** |
| **CBD-FS** | 12·5 (10·0) | 11·8 (12·1) | 15·8 (8·5) | 8·4 (6·2) | 9·6 (12·0) | 11·1 (19·0) | 4·2 (5·7) | 23·3 (10·2) | ·· | ·· | ·· | 13·4 (9·0) |
| **SEADL** | -12·0 (21·1) | -15·9 (19·4) | -14·5 (7·5) | -5·2 (25·7) | -13·3 (13·3) | -10·7 (16·4) | -8·4 (9·7) | -16·5 (14·0) | -8·8 (15·8) | -7·2 (10·8) | -10·4 (19·0) | -3·4 (21·4) |
| **Milestones** | 0·9 (1·3) | 1·0 (1·5) | 0·9 (0·9) | 0·9 (1·2) | 0·8 (1·3) | 1·5 (2·4) | 0·8 (1·0) | 0·7 (1·2) | 0·7 (1·0) | 0·7 (1·1) | 0·6 (0·9) | 0·4 (1·1) |
| **MSA QoL** | ·· | ·· | ·· | ·· | ·· | ·· | ·· | ·· | 3·8 (14·0) | -1·9 (13·2) | 9·8 (13·8) | ·· |
| **PSP QoL** | 14·8 (20·1) | 18·4 (22·3) | 16·7 (19·8) | 9·2 (17·1) | 14·9 (20·2) | 15·7 (25·9) | 8·6 (12·3) | 22·0 (23·8) | ·· | ·· | ·· | 18·6 (15·8) |
| **UPDRS II** | 5·7 (6·4) | 7·1 (6·1) | 11·6 (6·6) | 1·9 (5·7) | 5·8 (5·9) | 4·9 (7·0) | 5·4 (5·7) | 7·1 (6·4) | 4·4 (7·0) | 3·4 (4·5) | 5·6 (9·1) | 3·8 (3·7) |
| **UPDRS III** | 9·3 (13·2) | 9·6 (13·7) | 13·3 (10·3) | 7·3 (13·2) | 7·5 (12·2) | 8·2 (21·2) | 7·3 (6·2) | 6·5 (12·5) | ·· | ·· | ·· | 9·6 (14·2) |
| **PSPRS** | 9·9 (8·9) | 11·6 (9·1) | 13·9 (8·0) | 5·9 (8·0) | 9·2 (9·1) | 11·0 (16·9) | 7·0 (4·5) | 10·4 (8·7) | ·· | ·· | ·· | 4·2 (6·9) |
| **mPSPRS** | 3·6 (3·2) | 4·0 (3·3) | 5·8 (2·9) | 2·3 (2·6) | 2·6 (3·0) | 2·3 (4·4) | 1·9 (1·9) | 3·4 (3·2) | ·· | ·· | ·· | 1·6 (2·8) |
| **UMSARS** | ·· | ·· | ·· | ·· | ·· | ·· | ·· | ·· | 9·5 (8·2) | 11·2 (8·6) | 7·0 (7·4) | ·· |
| **UPDRS I** | 0·6 (3·7) | 0·4 (4·0) | 3·6 (3·3) | 0·2 (2·9) | 1·6 (3·5) | 1·6 (4·5) | 1·7 (2·8) | 1·7 (3·8) | 0·04 (4·1) | -0·8 (3·8) | 1·2 (4·3) | 1·5 (3·8) |
| **CBI-R** | 8·8 (20·1) | 15·2 (21·8) | 7·3 (12·9) | -1·1 (15·3) | 12·9 (18·4) | 14·4 (24·3) | 7·7 (10·1) | 18·1 (21·7) | 12·0 (22·4) | 17·8 (22·5) | 5·6 (21·0) | 10·2 (13·8) |
| **MoCA** | -0·9 (3·5) | -0·6 (3·0) | -3·7 (2·3) | -0·6 (4·3) | -1·5 (3·5) | -2·7 (5·1) | -2·4 (3·3) | -0·7 (3·0) | 0·2 (3·1) | -0·2 (2·6) | 0·7 (3·7) | -0·9 (3·0) |
| **ACE III** | -1·7 (5·3) | -1·4 (5·3) | -5·2 (6·9) | -1·1 (4·7) | -7·1 (10·2) | -8·1 (16·6) | -13·5 (11·9) | -5·0 (6·6) | -1·3 (6·9) | -0·8 (7·1) | -2·2 (6·6) | -2·3 (4·0) |
| **ECAS** | -3·1 (12·9) | -4·5 (10·7) | -12·9 (13·6) | 1·4 (15·2) | -3·3 (10·8) | 2·7 (11·1) | -6·3 (11·9) | -4·9 (10·3) | -4·8 (11·2) | -3·6 (9·4) | -6·2 (12·7) | 2·1 (12·0) |
| **FTD-FRS** | 2·7 (8·7) | 4·3 (8·5) | 4·6 (9·1) | -1·7 (8·1) | 2·1 (10·0) | 5·4 (15·1) | 2·2 (8·7) | 1·2 (8·9) | 1·1 (8·2) | 1·8 (5·4) | 1·0 (9·2) | 3·0 (3·3) |
| Variables with fewer than 5 responses per group have been removed. Abbreviations: 4RT = 4-repeat tau, AD = Alzheimer disease, ACE III = Addenbrookes Cognitive Examination III, CBD-FS = Corticobasal Degeneration Functional Scale, CBI-R = Cambridge Behavioural Inventory Revised, CBS = corticobasal syndrome , ECAS = Edinburgh Cognitive and Behavioural Screen, FTD-FRS = Frontotemporal Dementia Rating Scale, IDT = Indeterminate, MoCA = Montreal Cognitive Assessment, mPSPRS = modified Progressive Supranuclear Palsy Rating Scale, MSA = multiple system atrophy, MSA QoL = MSA Quality of life scale, PSP = progressive supranuclear palsy, PSP QoL = PSP Quality of life scale, PSPRS = PSP rating scale, SEADL = Schwab and England Activities of Daily Living Scale, UPDRS = Unified Parkinson’s Disease Rating Scale, UMSARS = Unified MSA Rating Scale | | | | | | | | | | | | |

**Supplementary Table 6: Annualised rates of progression in neuroimaging markers (percentage change from baseline) by Intention to Treat**

|  | **PSP** | | | | **CBS** | | | | **MSA** | | | **IDT** |
| --- | --- | --- | --- | --- | --- | --- | --- | --- | --- | --- | --- | --- |
|  | **All**  **(n=29)** | **RS**  **(n=18)** | **Cortical**  **(n=1)** | **Subcortical**  **(n=11)** | **All**  **(n=9)** | **4RT**  **(n=2)** | **AD**  **(n=1)** | **Unknown**  **(n=6)** | **All**  **(n=15)** | **Parkinsonism**  **(n=10)** | **Cerebellar**  **(n=5)** | **(n=5)** |
| **Temporal lobe** | -2·8 (2·3) | -2·9 (2·4) | ·· | -2·8 (2·2) | -1·8 (4·0) | ·· | ·· | -0·4 (1·6) | -3·3 (2·8) | -3·7 (2·5) | -2·4 (3·3) | -2·8 (1·3) |
| **Cingulate** | -2·0 (2·1) | -2·4 (2·3) | ·· | -1·3 (1·5) | -2·4 (2·9) | ·· | ·· | -1·8 (2·5) | -2·8 (2·8) | -2·2 (1·8) | -4·0 (4·2) | -2·9 (1·9) |
| **Insula** | -1·3 (2·5) | -1·0 (2·8) | ·· | -1·9 (2·0) | -1·5 (3·2) | ·· | ·· | -0·7 (3·0) | -2·2 (3·4) | -1·7 (2·6) | -3·3 (4·8) | -1·3 (1·2) |
| **Occipital lobe** | -2·7 (2·8) | -2·7 (3·0) | ·· | -2·7 (2·6) | -1·1 (4·4) | ·· | ·· | -1·0 (2·4) | -3·0 (3·3) | -3·6 (3·2) | -1·7 (3·5) | -2·0 (2·1) |
| **Frontal lobe** | -3·8 (3·0) | -4·4 (3·2) | ·· | -2·9 (2·5) | -3·0 (4·4) | ·· | ·· | -1·7 (4·1) | -4·2 (4·1) | -3·6 (3·6) | -5·5 (5·1) | -3·3 (1·9) |
| **Parietal lobe** | -3·0 (2·9) | -3·2 (3·0) | ·· | -2·8 (2·8) | -2·6 (5·9) | ·· | ·· | -0·5 (5·1) | -3·9 (3·6) | -4·0 (3·4) | -3·7 (4·4) | -2·2 (1·7) |
| **Central structures** | -1·9 (3·4) | -3·0 (1·9) | ·· | -0·03 (4·6) | -3·1 (2·2) | ·· | ·· | -3·2 (2·6) | -3·3 (3·0) | -2·5 (1·7) | -4·8 (4·6) | -2·3 (2·8) |
| **Ventricles** | 10·7 (6·7) | 12·4 (7·5) | ·· | 8·0 (4·0) | 13·3 (9·2) | ·· | ·· | 12·0 (9·5) | 15·5 (13·0) | 12·6 (6·0) | 21·3 (21·2) | 13·2 (9·1) |
| **Cerebellum** | -3·7 (4·3) | -4·1 (3·9) | ·· | -3·0 (5·0) | -2·1 (3·7) | ·· | ·· | -1·6 (2·4) | -5·9 (4·0) | -6·6 (3·4) | -4·4 (5·1) | -3·0 (1·6) |
| **Medulla** | 1·0 (7·0) | 0·3 (5·9) | ·· | 2·1 (8·7) | 0·3 (2·7) | ·· | ·· | 0·4 (2·5) | -1·0 (3·0) | -0·7 (3·4) | -1·4 (2·1) | 0·4 (1·8) |
| **Pons** | -2·2 (3·0)^a^ | -3·3 (2·9) | ·· | -0·3 (2·3) | -2·4 (4·7)^a^ | ·· | ·· | -3·2 (5·3) | -6·7 (4·7) | -6·3 (5·2) | -7·7 (4·0) | -0·4 (1·5)^a^ |
| **Midbrain** | -0·5 (4·8) | -2·4 (2·6) | ·· | 2·8 (5·9) | -1·2 (2·9) | ·· | ·· | -2·2 (2·4) | -2·6 (3·7) | -1·2 (1·9) | -5·5 (4·9) | 0·3 (4·5) |
| **Midbrain:Pons ratio** | 1·0 (7·0)^a^ | 0·3 (5·9) | ·· | 2·1 (8·7) | 0·3 (2·7)^a^ | ·· | ·· | 0·4 (2·5) | -1·0 (3·0) | -0·7 (3·4) | -1·4 (2·1) | 0·4 (1·8) |
| Abbreviations: 4RT = 4-repeat tau, AD = Alzheimer disease, CBS = corticobasal syndrome, IDT = Indeterminate, MSA = multiple system atrophy, PSP = progressive supranuclear palsy  a. Tukey post hoc test (TPH) adjusted *p* < 0·05 vs MSA-all | | | | | | | | | | | | |

**Supplementary Table 7: Sample sizes required for a 2-arm, 1-year follow-up therapeutic trial to detect 25% and 50% change by Final diagnosis**

|  | **PSP** | | | | | **CBS** | | | | | **MSA** | | | | |
| --- | --- | --- | --- | --- | --- | --- | --- | --- | --- | --- | --- | --- | --- | --- | --- |
|  |  | **25% change** | | **50% change** | |  | **25% change** | | **50% change** | |  | **25% change** | | **50% change** | |
|  | **Difference mean (SD)** | **Effect**  **size** | **Sample**  **size^a^** | **Effect**  **size** | **Sample**  **size^a^** | **Difference**  **mean (SD)** | **Effect**  **size** | **Sample**  **size^a^** | **Effect**  **size** | **Sample**  **size^a^** | **Difference**  **mean (SD)** | **Effect**  **size** | **Sample**  **size^a^** | **Effect**  **size** | **Sample**  **size^a^** |
| **CBD-FS** | 12·3 (12·5) | 0·246 | 260 (347) | 0·491 | 66 (88) | 8·6 (10·9) | 0·198 | 401 (535) | 0·397 | 101 (135) | ·· | ·· | ·· | ·· | ·· |
| **SEADL** | -12·6 (19·2) | 0·163 | 592 (789) | 0·326 | 149 (199) | -12·7 (12·4) | 0·256 | 240 (320) | 0·512 | 61 (81) | -9·6 (16·8) | 0·143 | 769 (1025) | 0·286 | 193 (257) |
| **Milestones** | 1·1 (1·4) | 0·194 | 418 (557) | 0·389 | 105 (140) | 0·5 (1·0) | 0·124 | 1022 (1363) | 0·248 | 256 (341) | 0·8 (1·1) | 0·171 | 538 (717) | 0·341 | 136 (181) |
| **MSA-QoL** | ·· | ·· | ·· | ·· | ·· | ·· | ·· | ·· | ·· | ·· | 3·8 (14·2) | 0·066 | >1500 | 0·133 | 888 (1184) |
| **PSP-QoL** | 15·0 (21·2) | 0·177 | 502 (669) | 0·354 | 126 (168) | 13·4 (18·5) | 0·181 | 480 (640) | 0·362 | 121 (161) | ·· | ·· | ·· | ·· | ·· |
| **UPDRS II** | 5·4 (6·4) | 0·213 | 347 (463) | 0·425 | 88 (117) | 6·2 (6·1) | 0·254 | 244 (325) | 0·508 | 62 (83) | 5·2 (7·1) | 0·183 | 470 (627) | 0·366 | 118 (157) |
| **UPDRS III** | 9·5 (14·3) | 0·167 | 564 (752) | 0·334 | 142 (189) | 6·3 (8·5) | 0·186 | 455 (607) | 0·373 | 114 (152) | ·· | ·· | ·· | ·· | ·· |
| **PSPRS** | 10·2 (9·5) | 0·267 | 221 (295) | 0·533 | 56 (75) | 8·1 (7·4) | 0·275 | 209 (279) | 0·550 | 53 (71) | ·· | ·· | ·· | ·· | ·· |
| **mPSPRS** | 3·8 (3·5) | 0·266 | 223 (297) | 0·532 | 56 (75) | 3·3 (3·4) | 0·247 | 258 (344) | 0·495 | 65 (87) | ·· | ·· | ·· | ·· | ·· |
| **UMSARS** | ·· | ·· | ·· | ·· | ·· | ·· | ·· | ·· | ·· | ·· | 9·5 (8·4) | 0·283 | 197 (263) | 0·567 | 50 (67) |
| **UPDRS I** | 0·7 (3·9) | 0·047 | >1500 | 0·094 | >1500 | 1·6 (3·0) | 0·136 | 850 (1133) | 0·272 | 213 (284) | 0·8 (4·2) | 0·049 | >1500 | 0·097 | >1500 |
| **CBI-R** | 9·3 (21·0) | 0·111 | 1275 (1700) | 0·223 | 317 (423) | 11·9 (17·4) | 0·170 | 544 (725) | 0·340 | 137 (183) | 11·9 (20·4) | 0·145 | 748 (997) | 0·291 | 186 (248) |
| **MoCA** | -1·1 (3·6) | 0·078 | >1500 | 0·156 | 646 (861) | -1·5 (2·9) | 0·133 | 888 (1184) | 0·266 | 223 (297) | 0·2 (2·9) | 0·020 | >1500 | 0·040 | >1500 |
| **ACE III** | -2·1 (7·4) | 0·070 | >1500 | 0·140 | 802 (1069) | -7·2 (8·0) | 0·226 | 308 (411) | 0·452 | 78 (104) | -1·3 (6·6) | 0·048 | >1500 | 0·096 | >1500 |
| **ECAS** | -3·0 (13·5) | 0·056 | >1500 | 0·112 | 1252 (1669) | -4·4 (10·4) | 0·106 | 1398 (1864) | 0·212 | 350 (467) | -4·3 (11·2) | 0·095 | >1500 | 0·189 | 440 (587) |
| **FTD-FRS** | 2·4 (8·9) | 0·067 | >1500 | 0·134 | 875 (1167) | 0·8 (10·1) | 0·020 | >1500 | 0·039 | >1500 | 1·4 (7·5) | 0·047 | >1500 | 0·093 | >1500 |
| a. Sample sizes are calculated per group, based on a significance level of 5% and a power of 80%; an approximation adjusting for a drop-out rate of 25% using the formula (e.g. 233/0.75=311) is presented in parentheses. Abbreviations: 4RT = 4-repeat tau, AD = Alzheimer disease, ACE III = Addenbrookes Cognitive Examination III, CBD-FS = Corticobasal Degeneration Functional Scale, CBI-R = Cambridge Behavioural Inventory Revised, CBS = corticobasal syndrome , ECAS = Edinburgh Cognitive and Behavioural Screen, FTD-FRS = Frontotemporal Dementia Rating Scale, IDT = Indeterminate, MoCA = Montreal Cognitive Assessment, mPSPRS = modified Progressive Supranuclear Palsy Rating Scale, MSA = multiple system atrophy, MSA QoL = MSA Quality of life scale, PSP = progressive supranuclear palsy, PSP QoL = PSP Quality of life scale, PSPRS = PSP rating scale, SD = standard deviation, SEADL = Schwab and England Activities of Daily Living Scale, UPDRS = Unified Parkinson’s Disease Rating Scale, UMSARS = Unified MSA Rating Scale.  Variables with fewer than 5 responses per group have been removed. | | | | | | | | | | | | | | | |

**Supplementary Table 8: Sample sizes required for a 2-arm, 1-year follow-up therapeutic trial to detect 25% and 50% change by Final diagnosis**

|  | **PSP** | | | | | **CBS** | | | | | | **MSA** | | | | |
| --- | --- | --- | --- | --- | --- | --- | --- | --- | --- | --- | --- | --- | --- | --- | --- | --- |
|  |  | **25% change** | | **50% change** | |  | **25% change** | | **50% change** | |  | | **25% change** | | **50% change** | |
|  | **Percent difference mean (SD)** | **Effect**  **size** | **Sample**  **size^a^** | **Effect**  **size** | **Sample**  **size^a^** | **Difference**  **mean (SD)** | **Effect**  **size** | **Sample**  **size^a^** | **Effect**  **size** | **Sample**  **size^a^** | **Difference**  **mean (SD)** | | **Effect**  **size** | **Sample**  **size^a^** | **Effect**  **size** | **Sample**  **size^a^** |
| **Temporal lobe** | -2·8 (2·3) | 0·260 | 233 (311) | 0·520 | 59 (79) | -1·9 (4·4) | 0·168 | 557 (743) | 0·336 | 140 (187) | -3·2 (2·6) | | 0·252 | 248 (331) | 0·504 | 63 (84) |
| **Cingulate** | -2·0 (2·1) | 0·256 | 240 (320) | 0·513 | 61 (81) | -1·9 (2·9) | 0·205 | 374 (499) | 0·409 | 95 (127) | -3·0 (2·7) | | 0·346 | 132 (176) | 0·693 | 34 (45) |
| **Insula** | -1·2 (2·5) | 0·146 | 737 (983) | 0·292 | 185 (247) | -1·0 (3·2) | 0·107 | 1372 (1829) | 0·214 | 344 (459) | -2·3 (3·2) | | 0·226 | 308 (411) | 0·451 | 78 (104) |
| **Occipital lobe** | -2·5 (2·6) | 0·204 | 378 (504) | 0·408 | 95 (127) | -1·7 (4·7) | 0·113 | 1230 (1640) | 0·225 | 311 (415) | -2·6 (3·3) | | 0·153 | 672 (896) | 0·307 | 168 (224) |
| **Frontal lobe** | -3·8 (3·1) | 0·334 | 142 (189) | 0·668 | 36 (48) | -2·1 (4·4) | 0·161 | 607 (809) | 0·322 | 152 (203) | -4·4 (3·9) | | 0·372 | 114 (152) | 0·744 | 29 (39) |
| **Parietal lobe** | -3·0 (2·9) | 0·284 | 196 (261) | 0·568 | 50 (67) | -2·3 (6·3) | 0·143 | 769 (1025) | 0·286 | 193 (257) | -3·9 (3·4) | | 0·336 | 140 (187) | 0·671 | 36 (48) |
| **Central**  **structures** | -1·9 (3·5) | 0·123 | 1039 (1385) | 0·245 | 262 (349) | -2·8 (2·2) | 0·342 | 135 (180) | 0·684 | 35 (47) | -3·6 (3·0) | | 0·471 | 72 (96) | 0·942 | 19 (25) |
| **Ventricles** | 11·0 (6·7) | 0·426 | 87 (116) | 0·852 | 23 (31) | 13·4 (10·1) | 0·304 | 171 (228) | 0·609 | 43 (57) | 15·9 (12·3) | | 0·274 | 210 (280) | 0·548 | 53 (71) |
| **Cerebellum** | -3·4 (4·1) | 0·231 | 295 (393) | 0·461 | 75 (100) | -1·9 (3·7) | 0·159 | 622 (829) | 0·319 | 155 (207) | -5·6 (3·9) | | 0·415 | 92 (123) | 0·829 | 24 (32) |
| **Medulla** | 0·9 (7·1) | 0·025 | >1500 | 0·050 | >1500 | -0·3 (2·5) | 0·004 | >1500 | 0·007 | >1500 | -0·6 (3·0) | | 0·034 | >1500 | 0·067 | >1500 |
| **Pons** | -2·2 (3·1) | 0·232 | 293 (391) | 0·464 | 74 (99) | -0·2 (1·1) | 0·029 | >1500 | 0·059 | >1500 | -7·1 (4·5) | | 0·387 | 106 (141) | 0·774 | 27 (36) |
| **Midbrain** | -0·5 (4·9) | 0·029 | >1500 | 0·059 | >1500 | -0·2 (2·1) | 0·007 | >1500 | 0·014 | >1500 | -2·8 (3·6) | | 0·296 | 180 (240) | 0·591 | 46 (61) |
| **Midbrain:Pons**  **ratio** | 0·9 (7·1) | 0·110 | 1298 (1731) | 0·220 | 325 (433) | -0·3 (2·5) | 0·015 | >1500 | 0·030 | >1500 | -0·6 (3·0) | | 0·293 | 184 (245) | 0·586 | 47 (63) |
| a. Sample sizes are calculated per group, based on a significance level of 5% and a power of 80%; an approximation adjusting for a drop-out rate of 25% using the formula (e.g. 233/0.75=311) is presented in parentheses. Abbreviations: 4RT = 4-repeat tau, AD = Alzheimer disease, CBS = corticobasal syndrome, IDT = Indeterminate, MSA = multiple system atrophy, PSP = progressive supranuclear palsy | | | | | | | | | | | | | | | | |

**Supplementary Table 9: Sample sizes required for a 2-arm, 1-year follow-up therapeutic trial to detect 25% and 50% change by Intention to Treat**

|  | **PSP** | | | | | **CBS** | | | | | | **MSA** | | | | |
| --- | --- | --- | --- | --- | --- | --- | --- | --- | --- | --- | --- | --- | --- | --- | --- | --- |
|  |  | **25% change** | | **50% change** | |  | **25% change** | | **50% change** | |  | | **25% change** | | **50% change** | |
|  | **Difference mean (SD)** | **Effect**  **size** | **Sample**  **size^a^** | **Effect**  **size** | **Sample**  **size^a^** | **Difference**  **mean (SD)** | **Effect**  **size** | **Sample**  **size^a^** | **Effect**  **size** | **Sample**  **size^a^** | **Difference**  **mean (SD)** | | **Effect**  **size** | **Sample**  **size^a^** | **Effect**  **size** | **Sample**  **size^a^** |
| **CBD-FS** | 12·5 (10·0) | 0·313 | 161 (215) | 0·627 | 41 (55) | 9·6 (12·0) | 0·201 | 390 (520) | 0·402 | 98 (131) | ·· | | ·· | ·· | ·· | ·· |
| **SEADL** | -12·0 (21·1) | 0·141 | 791 (1055) | 0·283 | 197 (263) | -13·3 (13·3) | 0·251 | 250 (333) | 0·502 | 63 (84) | -8·8 (15·8) | | 0·139 | 813 (1084) | 0·278 | 204 (272) |
| **Milestones** | 0·9 (1·3) | 0·180 | 485 (647) | 0·359 | 123 (164) | 0·8 (1·3) | 0·148 | 718 (957) | 0·297 | 179 (239) | 0·7 (1·0) | | 0·168 | 557 (743) | 0·336 | 140 (187) |
| **MSA-QoL** | ·· | ·· | ·· | ·· | ·· | ·· | ·· | ·· | ·· | ·· | 3·8 (14·0) | | 0·067 | >1500 | 0·134 | 875 (1167) |
| **PSP-QoL** | 14·8 (20·1) | 0·185 | 460 (613) | 0·369 | 116 (155) | 14·9 (20·2) | 0·184 | 465 (620) | 0·369 | 116 (155) | ·· | | ·· | ·· | ·· | ·· |
| **UPDRS II** | 5·7 (6·4) | 0·221 | 322 (429) | 0·443 | 81 (108) | 5·8 (5·9) | 0·243 | 267 (356) | 0·485 | 68 (91) | 4·4 (7·0) | | 0·155 | 654 (872) | 0·310 | 164 (219) |
| **UPDRS III** | 9·3 (13·2) | 0·176 | 508 (677) | 0·352 | 128 (171) | 7·5 (12·2) | 0·154 | 663 (884) | 0·308 | 166 (221) | ·· | | ·· | ·· | ·· | ·· |
| **PSPRS** | 9·9 (8·9) | 0·278 | 204 (272) | 0·556 | 52 (69) | 9·2 (9·1) | 0·253 | 246 (328) | 0·505 | 63 (84) | ·· | | ·· | ·· | ·· | ·· |
| **mPSPRS** | 3·6 (3·2) | 0·285 | 194 (259) | 0·571 | 49 (65) | 2·6 (3·0) | 0·216 | 337 (449) | 0·432 | 85 (113) | ·· | | ·· | ·· | ·· | ·· |
| **UMSARS** | ·· | ·· | ·· | ·· | ·· | ·· | ·· | ·· | ·· | ·· | 9·5 (8·2) | | 0·288 | 190 (253) | 0·576 | 48 (64) |
| **UPDRS I** | 0·6 (3·7) | 0·044 | >1500 | 0·088 | >1500 | 1·6 (3·5) | 0·116 | 1168 (1557) | 0·233 | 290 (387) | 0·04 (4·1) | | 0·002 | >1500 | 0·004 | >1500 |
| **CBI-R** | 8·8 (20·1) | 0·109 | 1322 (1763) | 0·218 | 331 (441) | 12·9 (18·4) | 0·175 | 514 (685) | 0·351 | 128 (171) | 12·0 (22·4) | | 0·134 | 875 (1167) | 0·268 | 220 (293) |
| **MoCA** | -0·9 (3·5) | 0·066 | >1500 | 0·132 | 902 (1203) | -1·5 (3·5) | 0·106 | 1398 (1864) | 0·212 | 350 (467) | 0·2 (3·1) | | 0·018 | >1500 | 0·035 | >1500 |
| **ACE III** | -1·7 (5·3) | 0·079 | >1500 | 0·158 | 630 (840) | -7·1 (10·2) | 0·175 | 514 (685) | 0·349 | 130 (173) | -1·3 (6·9) | | 0·047 | >1500 | 0·095 | >1500 |
| **ECAS** | -3·1 (12·9) | 0·061 | >1500 | 0·121 | 1073 (1431) | -3·3 (10·8) | 0·077 | >1500 | 0·154 | 663 (884) | -4·8 (11·2) | | 0·107 | >1500 | 0·215 | 341 (455) |
| **FTD-FRS** | 2·7 (8·7) | 0·076 | >1500 | 0·152 | 680 (907) | 2·1 (10·0) | 0·052 | >1500 | 0·105 | 1425 (1900) | 1·1 (8·2) | | 0·034 | >1500 | 0·069 | >1500 |
| a. Sample sizes are calculated per group, based on a significance level of 5% and a power of 80%; an approximation adjusting for a drop-out rate of 25% using the formula (e.g., 233/0.75=311) is presented in parentheses. Abbreviations: 4RT = 4-repeat tau, AD = Alzheimer disease, ACE III = Addenbrookes Cognitive Examination III, CBD-FS = Corticobasal Degeneration Functional Scale, CBI-R = Cambridge Behavioural Inventory Revised, CBS = corticobasal syndrome , ECAS = Edinburgh Cognitive and Behavioural Screen, FTD-FRS = Frontotemporal Dementia Rating Scale, IDT = Indeterminate, MoCA = Montreal Cognitive Assessment, mPSPRS = modified Progressive Supranuclear Palsy Rating Scale, MSA = multiple system atrophy, MSA QoL = MSA Quality of life scale, PSP = progressive supranuclear palsy, PSP QoL = PSP Quality of life scale, PSPRS = PSP rating scale, SD = standard deviation, SEADL = Schwab and England Activities of Daily Living Scale, UPDRS = Unified Parkinson’s Disease Rating Scale, UMSARS = Unified MSA Rating Scale | | | | | | | | | | | | | | | | |

**Supplementary Table 10: Sample sizes required for a 2-arm, 1-year follow-up therapeutic trial to detect 25% and 50% change by Intention to Treat**

|  | **PSP** | | | | | **CBS** | | | | | | **MSA** | | | | |
| --- | --- | --- | --- | --- | --- | --- | --- | --- | --- | --- | --- | --- | --- | --- | --- | --- |
|  |  | **25% change** | | **50% change** | |  | **25% change** | | **50% change** | |  | | **25% change** | | **50% change** | |
|  | **Percent difference mean (SD)** | **Effect**  **size** | **Sample**  **size^a^** | **Effect**  **size** | **Sample**  **size^a^** | **Difference**  **mean (SD)** | **Effect**  **size** | **Sample**  **size^a^** | **Effect**  **size** | **Sample**  **size^a^** | **Difference**  **mean (SD)** | | **Effect**  **size** | **Sample**  **size^a^** | **Effect**  **size** | **Sample**  **size^a^** |
| **Temporal lobe** | -2·8 (2·3) | 0·269 | 218 (291) | 0·538 | 55 (73) | -1·8 (4·0) | 0·167 | 564 (752) | 0·334 | 142 (189) | -3·3 (2·8) | | 0·256 | 240 (320) | 0·512 | 61 (81) |
| **Cingulate** | -2·0 (2·1) | 0·259 | 235 (313) | 0·518 | 59 (79) | -2·4 (2·9) | 0·265 | 224 (299) | 0·530 | 57 (76) | -2·8 (2·8) | | 0·324 | 151 (201) | 0·647 | 38 (51) |
| **Insula** | -1·3 (2·5) | 0·156 | 646 (861) | 0·311 | 163 (217) | -1·5 (3·2) | 0·164 | 585 (780) | 0·328 | 147 (196) | -2·2 (3·4) | | 0·214 | 344 (459) | 0·427 | 87 (116) |
| **Occipital lobe** | -2·7 (2·8) | 0·208 | 364 (485) | 0·417 | 91 (121) | -1·1 (4·4) | 0·079 | >1500 | 0·159 | 622 (829) | -3·0 (3·3) | | 0·177 | 502 (669) | 0·354 | 126 (168) |
| **Frontal lobe** | -3·8 (3·0) | 0·341 | 136 (181) | 0·681 | 35 (47) | -3·0 (4·4) | 0·238 | 278 (371) | 0·477 | 70 (93) | -4·2 (4·1) | | 0·345 | 133 (177) | 0·690 | 34 (45) |
| **Parietal lobe** | -3·0 (2·9) | 0·291 | 186 (248) | 0·583 | 47 (63) | -2·6 (5·9) | 0·171 | 538 (717) | 0·343 | 134 (179) | -3·9 (3·6) | | 0·334 | 142 (189) | 0·667 | 36 (48) |
| **Central**  **structures** | -1·9 (3·4) | 0·121 | 1073 (1431) | 0·243 | 267 (356) | -3·1 (2·2) | 0·393 | 103 (137) | 0·785 | 26 (35) | -3·3 (3·0) | | 0·425 | 88 (117) | 0·849 | 23 (31) |
| **Ventricles** | 10·7 (6·7) | 0·422 | 89 (119) | 0·843 | 23 (31) | 13·3 (9·2) | 0·388 | 105 (140) | 0·776 | 27 (36) | 15·5 (13·0) | | 0·244 | 265 (353) | 0·487 | 67 (89) |
| **Cerebellum** | -3·7 (4·3) | 0·236 | 283 (377) | 0·473 | 71 (95) | -2·1 (3·7) | 0·199 | 397 (529) | 0·398 | 100 (133) | -5·9 (4·0) | | 0·447 | 80 (107) | 0·893 | 21 (28) |
| **Medulla** | 1·0 (7·0) | 0·028 | >1500 | 0·055 | >1500 | 0·3 (2·7) | 0·075 | >1500 | 0·150 | 699 (932) | -1·0 (3·0) | | 0·070 | >1500 | 0·140 | 802 (1069) |
| **Pons** | -2·2 (3·0) | 0·234 | 288 (384) | 0·468 | 73 (97) | -2·4 (4·7) | 0·187 | 450 (600) | 0·374 | 113 (151) | -6·7 (4·7) | | 0·339 | 138 (184) | 0·678 | 35 (47) |
| **Midbrain** | -0·5 (4·8) | 0·029 | >1500 | 0·057 | >1500 | -1·2 (2·9) | 0·119 | 1109 (1479) | 0·238 | 278 (371) | -2·6 (3·7) | | 0·279 | 203 (271) | 0·558 | 51 (68) |
| **Midbrain:Pons**  **ratio** | 1·0 (7·0) | 0·111 | 1275 (1700) | 0·222 | 319 (425) | 0·3 (2·7) | 0·160 | 614 (819) | 0·320 | 154 (205) | -1·0 (3·0) | | 0·257 | 239 (319) | 0·514 | 60 (80) |
| a. Sample sizes are calculated per group, based on a significance level of 5% and a power of 80%; an approximation adjusting for a drop-out rate of 25% using the formula (e.g. 233/0.75=311) is presented in parentheses. Abbreviations: 4RT = 4-repeat tau, AD = Alzheimer disease, CBS = corticobasal syndrome, IDT = Indeterminate, MSA = multiple system atrophy, PSP = progressive supranuclear palsy | | | | | | | | | | | | | | | | |

**Supplementary Table 11: Sample sizes required for a 2-arm, 1-year follow-up therapeutic trial to detect 20%, 25%, 30%, 40% and 50% change by Final diagnosis in PSP phenotypes**

| **PSP** |  | **20% change** | | **25% change** | | **30% change** | | **40% change** | | **50% change** | |
| --- | --- | --- | --- | --- | --- | --- | --- | --- | --- | --- | --- |
|  | **Difference mean (SD)** | **Effect size** | **Sample size** | **Effect size** | **Sample size** | **Effect size** | **Sample size** | **Effect size** | **Sample size** | **Effect size** | **Sample size** |
| **RS** |  |  |  |  |  |  |  |  |  |  |  |
| CBD-FS | 12·6 (12·1) | 0·209 | 360 | 0·262 | 230 | 0·314 | 160 | 0·419 | 90 | 0·523 | 58 |
| SEADL | -12·7 (22·0) | 0·116 | 1168 | 0·145 | 748 | 0·174 | 519 | 0·232 | 293 | 0·290 | 188 |
| Milestones | 0·9 (1·4) | 0·131 | 916 | 0·163 | 592 | 0·196 | 410 | 0·261 | 231 | 0·326 | 149 |
| PSP-QoL | 19·0 (21·6) | 0·176 | 508 | 0·220 | 325 | 0·265 | 224 | 0·353 | 127 | 0·441 | 82 |
|  |  |  |  |  |  |  |  |  |  |  |  |
| UPDRS II | 6·7 (6·5) | 0·209 | 360 | 0·261 | 231 | 0·313 | 161 | 0·417 | 91 | 0·521 | 59 |
| UPDRS III | 8·6 (14·3) | 0·120 | 1091 | 0·150 | 699 | 0·180 | 485 | 0·240 | 273 | 0·300 | 175 |
| PSPRS | 10·7 (9·3) | 0·229 | 300 | 0·286 | 193 | 0·343 | 134 | 0·458 | 76 | 0·572 | 49 |
| mPSPRS | 3·8 (3·3) | 0·228 | 303 | 0·286 | 193 | 0·343 | 134 | 0·457 | 76 | 0·571 | 49 |
|  |  |  |  |  |  |  |  |  |  |  |  |
| UPDRS I | 0·3 (4·1) | 0·013 | 92887 | 0·016 | 61320 | 0·019 | 43485 | 0·026 | 23222 | 0·032 | 15331 |
| CBI-R | 14·9 (21·9) | 0·136 | 850 | 0·170 | 544 | 0·204 | 378 | 0·272 | 213 | 0·340 | 137 |
| MoCA | -0·8 (3·0) | 0·055 | 5190 | 0·069 | 3298 | 0·082 | 2336 | 0·110 | 1298 | 0·137 | 837 |
| ACE III | -1·8 (5·1) | 0·071 | 3115 | 0·089 | 1983 | 0·106 | 1398 | 0·142 | 779 | 0·177 | 502 |
| ECAS | -6·7 (11·3) | 0·119 | 1109 | 0·149 | 708 | 0·179 | 491 | 0·239 | 276 | 0·299 | 177 |
| FTD-FRS | 4·8 (8·7) | 0·111 | 1275 | 0·139 | 813 | 0·166 | 571 | 0·222 | 319 | 0·277 | 206 |
|  |  |  |  |  |  |  |  |  |  |  |  |
| **Cortical** |  |  |  |  |  |  |  |  |  |  |  |
| CBD-FS | 14·1 (13·2) | 0·213 | 347 | 0·266 | 223 | 0·319 | 155 | 0·426 | 87 | 0·532 | 56 |
| SEADL | -12·2 (11·4) | 0·214 | 344 | 0·268 | 220 | 0·321 | 153 | 0·428 | 87 | 0·535 | 56 |
| Milestones | 1·6 (1·8) | 0·174 | 519 | 0·218 | 331 | 0·262 | 230 | 0·349 | 130 | 0·436 | 84 |
| PSP-QoL | 15·3 (23·9) | 0·128 | 959 | 0·160 | 614 | 0·192 | 427 | 0·255 | 242 | 0·319 | 155 |
|  |  |  |  |  |  |  |  |  |  |  |  |
| UPDRS II | 6·9 (6·5) | 0·212 | 350 | 0·265 | 224 | 0·318 | 156 | 0·424 | 88 | 0·531 | 57 |
| UPDRS III | 13·9 (15·0) | 0·185 | 460 | 0·231 | 295 | 0·277 | 206 | 0·370 | 116 | 0·462 | 75 |
| PSPRS | 14·8 (11·2) | 0·264 | 226 | 0·330 | 145 | 0·396 | 101 | 0·529 | 57 | 0·661 | 37 |
| mPSPRS | 4·3 (4·0) | 0·215 | 341 | 0·269 | 218 | 0·322 | 152 | 0·430 | 86 | 0·537 | 55 |
|  |  |  |  |  |  |  |  |  |  |  |  |
| UPDRS I | 2·0 (4·3) | 0·092 | 1856 | 0·115 | 1188 | 0·138 | 825 | 0·183 | 470 | 0·229 | 300 |
| CBI-R | 12·1 (20·4) | 0·119 | 1109 | 0·148 | 718 | 0·178 | 496 | 0·237 | 280 | 0·296 | 180 |
| MoCA | -3·5 (4·2) | 0·169 | 551 | 0·211 | 354 | 0·253 | 246 | 0·338 | 138 | 0·422 | 89 |
| ACE III | -7·1 (14·6) | 0·098 | 1635 | 0·122 | 1056 | 0·146 | 737 | 0·195 | 414 | 0·244 | 265 |
| ECAS | -3·9 (10·2) | 0·077 | 2649 | 0·096 | 1704 | 0·115 | 1188 | 0·154 | 663 | 0·192 | 427 |
| FTD-FRS | 0·9 (8·7) | 0·021 | 35597 | 0·027 | 21534 | 0·032 | 15331 | 0·043 | 8491 | 0·054 | 5384 |
|  |  |  |  |  |  |  |  |  |  |  |  |
| **Subcortical** |  |  |  |  |  |  |  |  |  |  |  |
| CBD-FS | 7·3 (7·3) | 0·202 | 386 | 0·252 | 248 | 0·302 | 173 | 0·403 | 98 | 0·504 | 63 |
| SEADL | -12·5 (17·4) | 0·144 | 758 | 0·180 | 485 | 0·216 | 337 | 0·288 | 190 | 0·360 | 122 |
| Milestones | 1·1 (1·2) | 0·185 | 460 | 0·231 | 295 | 0·277 | 206 | 0·369 | 116 | 0·461 | 75 |
| PSP-QoL | 9·0 (17·7) | 0·102 | 1510 | 0·127 | 974 | 0·153 | 672 | 0·204 | 378 | 0·255 | 242 |
|  |  |  |  |  |  |  |  |  |  |  |  |
| UPDRS II | 2·4 (5·4) | 0·088 | 2028 | 0·110 | 1298 | 0·132 | 902 | 0·176 | 508 | 0·220 | 325 |
| UPDRS III | 9·0 (13·5) | 0·133 | 888 | 0·166 | 571 | 0·199 | 397 | 0·265 | 224 | 0·331 | 144 |
| PSPRS | 6·9 (8·1) | 0·171 | 538 | 0·214 | 344 | 0·257 | 239 | 0·342 | 135 | 0·428 | 87 |
| mPSPRS | 2·6 (2·6) | 0·203 | 382 | 0·253 | 246 | 0·304 | 171 | 0·405 | 97 | 0·507 | 62 |
|  |  |  |  |  |  |  |  |  |  |  |  |
| UPDRS I | 0·7 (2·6) | 0·051 | 6036 | 0·064 | 3833 | 0·077 | 2649 | 0·102 | 1510 | 0·128 | 959 |
| CBI-R | -1·0 (15·5) | 0·013 | 92887 | 0·016 | 61320 | 0·019 | 43485 | 0·026 | 23222 | 0·032 | 15331 |
| MoCA | -0·6 (4·2) | 0·029 | 18667 | 0·036 | 12113 | 0·044 | 8109 | 0·058 | 4667 | 0·073 | 2947 |
| ACE III | -1·1 (4·6) | 0·048 | 6814 | 0·061 | 4220 | 0·073 | 2947 | 0·097 | 1669 | 0·121 | 1073 |
| ECAS | 2·8 (14·3) | 0·040 | 9812 | 0·049 | 6539 | 0·059 | 4511 | 0·079 | 2516 | 0·099 | 1603 |
| FTD-FRS | -1·6 (7·8) | 0·041 | 9339 | 0·052 | 5806 | 0·062 | 4085 | 0·083 | 2280 | 0·104 | 1452 |
|  | | | | | | | | | | | |
| Variables with fewer than 5 responses per group have been removed. Abbreviations: ACE III = Addenbrookes Cognitive Examination III, CBD-FS = Corticobasal Degeneration Functional Scale, CBI-R = Cambridge Behavioural Inventory Revised, CBS = corticobasal syndrome , ECAS = Edinburgh Cognitive and Behavioural Screen, FTD-FRS = Frontotemporal Dementia Rating Scale, IDT = Indeterminate, MoCA = Montreal Cognitive Assessment, mPSPRS = modified Progressive Supranuclear Palsy Rating Scale, PSP = progressive supranuclear palsy, PSP QoL = PSP Quality of life scale, PSPRS = PSP rating scale, SD = standard deviation, SEADL = Schwab and England Activities of Daily Living Scale, UPDRS = Unified Parkinson’s Disease Rating Scale | | | | | | | | | | | |

**Supplementary Table 12: Sample sizes required for a 2-arm, 1-year follow-up therapeutic trial to detect 20%, 25%, 30%, 40% and 50% change by Final diagnosis in CBS phenotypes**

| **CBS** |  | **20% change** | | **25% change** | | **30% change** | | **40% change** | | **50% change** | |
| --- | --- | --- | --- | --- | --- | --- | --- | --- | --- | --- | --- |
|  | **Difference mean (SD)** | **Effect size** | **Sample size** | **Effect size** | **Sample size** | **Effect size** | **Sample size** | **Effect size** | **Sample size** | **Effect size** | **Sample size** |
| **4RT** |  |  |  |  |  |  |  |  |  |  |  |
| SEADL | -14·7 (10·3) | 0·284 | 196 | 0·355 | 126 | 0·426 | 87 | 0·568 | 50 | 0·709 | 32 |
| Milestones | 0·9 (1·6) | 0·114 | 1209 | 0·143 | 769 | 0·172 | 532 | 0·229 | 300 | 0·286 | 193 |
| PSP-QoL | 27·9 (23·9) | 0·233 | 290 | 0·292 | 185 | 0·350 | 129 | 0·467 | 73 | 0·584 | 47 |
|  |  |  |  |  |  |  |  |  |  |  |  |
| UPDRS II | 10·3 (7·4) | 0·279 | 203 | 0·349 | 130 | 0·418 | 91 | 0·558 | 51 | 0·697 | 33 |
| UPDRS III | 10·0 (10·3) | 0·194 | 418 | 0·242 | 269 | 0·291 | 186 | 0·388 | 105 | 0·484 | 68 |
| PSPRS | 7·1 (9·7) | 0·146 | 737 | 0·183 | 470 | 0·219 | 328 | 0·292 | 185 | 0·366 | 118 |
| mPSPRS | 3·2 (2·5) | 0·253 | 246 | 0·316 | 158 | 0·379 | 110 | 0·506 | 62 | 0·632 | 40 |
|  |  |  |  |  |  |  |  |  |  |  |  |
| UPDRS I | 2·8 (3·4) | 0·165 | 578 | 0·206 | 371 | 0·248 | 256 | 0·330 | 145 | 0·413 | 93 |
| CBI-R | 16·2 (14·7) | 0·221 | 322 | 0·276 | 207 | 0·331 | 144 | 0·442 | 81 | 0·552 | 52 |
| MoCA | -1·6 (2·7) | 0·117 | 1148 | 0·146 | 737 | 0·176 | 508 | 0·234 | 288 | 0·293 | 184 |
| ACE III | -6·8 (8·7) | 0·156 | 646 | 0·195 | 414 | 0·234 | 288 | 0·312 | 162 | 0·390 | 104 |
| ECAS | 2·3 (10·4) | 0·045 | 7753 | 0·056 | 5007 | 0·068 | 3396 | 0·090 | 1939 | 0·113 | 1230 |
| FTD-FRS | -1·5 (15·8) | 0·020 | 39245 | 0·024 | 27254 | 0·029 | 18667 | 0·039 | 10322 | 0·049 | 6539 |
|  |  |  |  |  |  |  |  |  |  |  |  |
| **AD** |  |  |  |  |  |  |  |  |  |  |  |
| SEADL | -11·7 (12·8) | 0·182 | 475 | 0·227 | 306 | 0·272 | 213 | 0·363 | 120 | 0·454 | 77 |
| Milestones | 0·4 (0·5) | 0·149 | 708 | 0·186 | 455 | 0·223 | 317 | 0·298 | 178 | 0·372 | 114 |
| PSP-QoL | 8·0 (14·4) | 0·111 | 1275 | 0·139 | 813 | 0·167 | 564 | 0·222 | 319 | 0·278 | 204 |
|  |  |  |  |  |  |  |  |  |  |  |  |
| UPDRS II | 5·1 (4·8) | 0·209 | 360 | 0·261 | 231 | 0·314 | 160 | 0·418 | 91 | 0·523 | 58 |
| UPDRS III | 6·1 (7·6) | 0·160 | 614 | 0·200 | 393 | 0·240 | 273 | 0·321 | 153 | 0·401 | 99 |
| PSPRS | 7·6 (5·4) | 0·281 | 200 | 0·351 | 128 | 0·422 | 89 | 0·562 | 51 | 0·703 | 33 |
| mPSPRS | 2·1 (2·4) | 0·173 | 525 | 0·216 | 337 | 0·260 | 233 | 0·346 | 132 | 0·433 | 85 |
|  |  |  |  |  |  |  |  |  |  |  |  |
| UPDRS I | 1·5 (3·0) | 0·098 | 1635 | 0·122 | 1056 | 0·147 | 727 | 0·196 | 410 | 0·245 | 262 |
| CBI-R | 6·6 (12·0) | 0·110 | 1298 | 0·138 | 825 | 0·165 | 578 | 0·221 | 322 | 0·276 | 207 |
| MoCA | -2·3 (3·1) | 0·151 | 689 | 0·189 | 440 | 0·227 | 306 | 0·302 | 173 | 0·378 | 111 |
| ACE III | -11·8 (10·0) | 0·235 | 285 | 0·293 | 184 | 0·352 | 128 | 0·469 | 72 | 0·586 | 47 |
| ECAS | -4·5 (10·7) | 0·084 | 2226 | 0·105 | 1425 | 0·126 | 990 | 0·167 | 564 | 0·209 | 360 |
| FTD-FRS | 5·9 (6·4) | 0·185 | 460 | 0·231 | 295 | 0·277 | 206 | 0·370 | 116 | 0·462 | 75 |
|  |  |  |  |  |  |  |  |  |  |  |  |
| **IDT** |  |  |  |  |  |  |  |  |  |  |  |
| SEADL | -12·2 (13·9) | 0·176 | 508 | 0·220 | 325 | 0·264 | 226 | 0·353 | 127 | 0·441 | 82 |
| Milestones | 0·4 (0·9) | 0·086 | 2123 | 0·108 | 1347 | 0·130 | 930 | 0·173 | 525 | 0·216 | 337 |
| PSP-QoL | 11·5 (19·0) | 0·121 | 1073 | 0·151 | 689 | 0·181 | 480 | 0·242 | 269 | 0·302 | 173 |
|  |  |  |  |  |  |  |  |  |  |  |  |
| UPDRS II | 7·1 (6·5) | 0·218 | 331 | 0·273 | 212 | 0·328 | 147 | 0·437 | 83 | 0·546 | 54 |
| UPDRS III | 5·0 (8·7) | 0·114 | 1209 | 0·142 | 779 | 0·171 | 538 | 0·227 | 306 | 0·284 | 196 |
| PSPRS | 9·4 (8·5) | 0·221 | 322 | 0·277 | 206 | 0·332 | 143 | 0·443 | 81 | 0·554 | 52 |
| mPSPRS | 2·7 (3·1) | 0·174 | 519 | 0·218 | 331 | 0·261 | 231 | 0·348 | 131 | 0·436 | 84 |
|  |  |  |  |  |  |  |  |  |  |  |  |
| UPDRS I | 0·5 (2·6) | 0·038 | 10872 | 0·047 | 7107 | 0·057 | 4833 | 0·075 | 2792 | 0·094 | 1778 |
| CBI-R | 16·4 (26·3) | 0·125 | 1006 | 0·156 | 646 | 0·187 | 450 | 0·250 | 252 | 0·312 | 162 |
| MoCA | -0·8 (3·6) | 0·047 | 7107 | 0·059 | 4511 | 0·071 | 3115 | 0·095 | 1740 | 0·119 | 1109 |
| ACE III | -4·3 (6·5) | 0·132 | 902 | 0·165 | 578 | 0·198 | 401 | 0·265 | 224 | 0·331 | 144 |
| ECAS | -7·3 (10·8) | 0·135 | 862 | 0·169 | 551 | 0·203 | 382 | 0·271 | 215 | 0·338 | 138 |
| FTD-FRS | 3·0 (9·5) | 0·064 | 3833 | 0·080 | 2454 | 0·095 | 1740 | 0·127 | 974 | 0·159 | 622 |
|  | | | | | | | | | | | |
| Variables with fewer than 5 responses per group have been removed. Abbreviations: 4RT = 4-repeat tau, AD = Alzheimer disease, ACE III = Addenbrookes Cognitive Examination III, CBD-FS = Corticobasal Degeneration Functional Scale, CBI-R = Cambridge Behavioural Inventory Revised, CBS = corticobasal syndrome , ECAS = Edinburgh Cognitive and Behavioural Screen, FTD-FRS = Frontotemporal Dementia Rating Scale, IDT = Indeterminate, MoCA = Montreal Cognitive Assessment, mPSPRS = modified Progressive Supranuclear Palsy Rating Scale, MSA = multiple system atrophy, MSA QoL = MSA Quality of life scale, PSP = progressive supranuclear palsy, PSP QoL = PSP Quality of life scale, PSPRS = PSP rating scale, SD = standard deviation, SEADL = Schwab and England Activities of Daily Living Scale, UPDRS = Unified Parkinson’s Disease Rating Scale, UMSARS = Unified MSA Rating Scale | | | | | | | | | | | |

**Supplementary Table 13: Sample sizes required for a 2-arm, 1-year follow-up therapeutic trial to detect 20%, 25%, 30%, 40% and 50% change by Final diagnosis in MSA phenotypes**

| **MSA** |  | **20% change** | | **25% change** | | **30% change** | | **40% change** | | **50% change** | |
| --- | --- | --- | --- | --- | --- | --- | --- | --- | --- | --- | --- |
|  | **Difference mean (SD)** | **Effect size** | **Sample size** | **Effect size** | **Sample size** | **Effect size** | **Sample size** | **Effect size** | **Sample size** | **Effect size** | **Sample size** |
| **MSA-C** |  |  |  |  |  |  |  |  |  |  |  |
| SEADL | -10·4 (19·0) | 0·109 | 1322 | 0·136 | 850 | 0·164 | 585 | 0·218 | 331 | 0·273 | 212 |
| Milestones | 0·6 (0·9) | 0·137 | 837 | 0·171 | 538 | 0·205 | 374 | 0·274 | 210 | 0·342 | 135 |
| MSA-QoL | 9·8 (13·8) | 0·141 | 791 | 0·177 | 502 | 0·212 | 350 | 0·282 | 198 | 0·353 | 127 |
|  |  |  |  |  |  |  |  |  |  |  |  |
| UPDRS II | 5·6 (9·1) | 0·123 | 1039 | 0·153 | 672 | 0·184 | 465 | 0·245 | 262 | 0·307 | 168 |
| UMSARS | 7·0 (7·4) | 0·190 | 436 | 0·237 | 280 | 0·284 | 196 | 0·379 | 110 | 0·474 | 71 |
|  |  |  |  |  |  |  |  |  |  |  |  |
| UPDRS I | 1·2 (4·3) | 0·056 | 5007 | 0·069 | 3298 | 0·083 | 2280 | 0·111 | 1275 | 0·139 | 813 |
| CBI-R | 5·6 (21·0) | 0·053 | 5589 | 0·066 | 3605 | 0·080 | 2454 | 0·106 | 1398 | 0·133 | 888 |
| MoCA | 0·7 (3·7) | 0·039 | 10322 | 0·049 | 6539 | 0·059 | 4511 | 0·079 | 2516 | 0·098 | 1635 |
| ACE III | -2·2 (6·6) | 0·067 | 3498 | 0·083 | 2280 | 0·100 | 1571 | 0·133 | 888 | 0·166 | 571 |
| ECAS | -6·2 (12·7) | 0·098 | 1635 | 0·123 | 1039 | 0·148 | 718 | 0·197 | 405 | 0·246 | 260 |
| FTD-FRS | 1·0 (9·2) | 0·021 | 35597 | 0·026 | 23222 | 0·031 | 16336 | 0·042 | 8900 | 0·052 | 5806 |
|  |  |  |  |  |  |  |  |  |  |  |  |
| **MSA-P** |  |  |  |  |  |  |  |  |  |  |  |
| SEADL | -9·0 (14·3) | 0·125 | 1006 | 0·157 | 638 | 0·188 | 445 | 0·251 | 250 | 0·313 | 161 |
| Milestones | 0·9 (1·2) | 0·142 | 779 | 0·178 | 496 | 0·213 | 347 | 0·284 | 196 | 0·355 | 126 |
| MSA-QoL | -1·9 (13·2) | 0·028 | 20024 | 0·035 | 12815 | 0·042 | 8900 | 0·057 | 4833 | 0·071 | 3115 |
|  |  |  |  |  |  |  |  |  |  |  |  |
| UPDRS II | 5·0 (5·6) | 0·179 | 491 | 0·224 | 314 | 0·269 | 218 | 0·358 | 123 | 0·448 | 79 |
| UMSARS | 11·2 (8·6) | 0·261 | 231 | 0·326 | 149 | 0·392 | 103 | 0·522 | 59 | 0·653 | 38 |
|  |  |  |  |  |  |  |  |  |  |  |  |
| UPDRS I | 0·6 (4·2) | 0·030 | 17443 | 0·037 | 11468 | 0·044 | 8109 | 0·059 | 4511 | 0·074 | 2868 |
| CBI-R | 15·7 (19·3) | 0·163 | 592 | 0·203 | 382 | 0·244 | 265 | 0·325 | 150 | 0·407 | 96 |
| MoCA | -0·1 (2·4) | 0·008 | 245278 | 0·010 | 156978 | 0·012 | 109013 | 0·015 | 69769 | 0·019 | 43485 |
| ACE III | -0·7 (6·7) | 0·022 | 32434 | 0·028 | 20024 | 0·033 | 14416 | 0·044 | 8109 | 0·055 | 5190 |
| ECAS | -2·5 (9·6) | 0·053 | 5589 | 0·066 | 3605 | 0·079 | 2516 | 0·106 | 1398 | 0·132 | 902 |
| FTD-FRS | 2·0 (4·4) | 0·090 | 1939 | 0·112 | 1252 | 0·135 | 862 | 0·179 | 491 | 0·224 | 314 |
|  | | | | | | | | | | | |
| Variables with fewer than 5 responses per group have been removed. Abbreviations: ACE III = Addenbrookes Cognitive Examination III, CBI-R = Cambridge Behavioural Inventory Revised, ECAS = Edinburgh Cognitive and Behavioural Screen, FTD-FRS = Frontotemporal Dementia Rating Scale, MoCA = Montreal Cognitive Assessment, MSA = multiple system atrophy, MSA-C = MSA cerebellar variant, MSA-P = MSA parkinsonism variant, MSA QoL = MSA Quality of life scale, SD = standard deviation, SEADL = Schwab and England Activities of Daily Living Scale, UPDRS = Unified Parkinson’s Disease Rating Scale, UMSARS = Unified MSA Rating Scale | | | | | | | | | | | |

**Supplementary Table 14: Sample sizes required for a 2-arm, 1-year follow-up therapeutic trial to detect 20%, 25%, 30%, 40% and 50% change by Final diagnosis according to disease group**

| **Group** |  | **20% change** | | **25% change** | | **30% change** | | **40% change** | | **50% change** | |
| --- | --- | --- | --- | --- | --- | --- | --- | --- | --- | --- | --- |
|  | **Percent difference mean (SD)** | **Effect size** | **Sample size** | **Effect size** | **Sample size** | **Effect size** | **Sample size** | **Effect size** | **Sample size** | **Effect size** | **Sample size** |
| **PSP** |  |  |  |  |  |  |  |  |  |  |  |
| Temporal | -2·8 (2·3) | 0·208 | 364 | 0·260 | 233 | 0·312 | 162 | 0·416 | 92 | 0·520 | 59 |
| Cingulate | -2·0 (2·1) | 0·205 | 374 | 0·256 | 240 | 0·308 | 166 | 0·410 | 94 | 0·513 | 61 |
| Insula | -1·2 (2·5) | 0·117 | 1148 | 0·146 | 737 | 0·175 | 514 | 0·234 | 288 | 0·292 | 185 |
| Occipital | -2·5 (2·6) | 0·163 | 592 | 0·204 | 378 | 0·245 | 262 | 0·326 | 149 | 0·408 | 95 |
| Frontal | -3·8 (3·1) | 0·267 | 221 | 0·334 | 142 | 0·401 | 99 | 0·534 | 56 | 0·668 | 36 |
| Parietal | -3·0 (2·9) | 0·227 | 306 | 0·284 | 196 | 0·341 | 136 | 0·454 | 77 | 0·568 | 50 |
| Central | -1·9 (3·5) | 0·098 | 1635 | 0·123 | 1039 | 0·147 | 727 | 0·196 | 410 | 0·245 | 262 |
| Ventricles | 11·0 (6·7) | 0·341 | 136 | 0·426 | 87 | 0·511 | 61 | 0·682 | 35 | 0·852 | 23 |
| Cerebellum | -3·4 (4·1) | 0·184 | 465 | 0·231 | 295 | 0·277 | 206 | 0·369 | 116 | 0·461 | 75 |
| Medulla | 0·9 (7·1) | 0·020 | 39245 | 0·025 | 25117 | 0·030 | 17443 | 0·040 | 9812 | 0·050 | 6280 |
| Pons | -2·2 (3·1) | 0·186 | 455 | 0·232 | 293 | 0·278 | 204 | 0·371 | 115 | 0·464 | 74 |
| Midbrain | -0·5 (4·9) | 0·023 | 29675 | 0·029 | 18667 | 0·035 | 12815 | 0·047 | 7107 | 0·059 | 4511 |
| Midbrain:Pons | 0·9 (7·1) | 0·088 | 2028 | 0·110 | 1298 | 0·132 | 902 | 0·176 | 508 | 0·220 | 325 |
| **CBS** |  |  |  |  |  |  |  |  |  |  |  |
| Temporal | -1·9 (4·4) | 0·134 | 875 | 0·168 | 557 | 0·202 | 386 | 0·269 | 218 | 0·336 | 140 |
| Cingulate | -1·9 (2·9) | 0·164 | 585 | 0·205 | 374 | 0·245 | 262 | 0·327 | 148 | 0·409 | 95 |
| Insula | -1·0 (3·2) | 0·086 | 2123 | 0·107 | 1372 | 0·128 | 959 | 0·171 | 538 | 0·214 | 344 |
| Occipital | -1·7 (4·7) | 0·090 | 1939 | 0·113 | 1230 | 0·135 | 862 | 0·180 | 485 | 0·225 | 311 |
| Frontal | -2·1 (4·4) | 0·129 | 944 | 0·161 | 607 | 0·193 | 422 | 0·258 | 237 | 0·322 | 152 |
| Parietal | -2·3 (6·3) | 0·114 | 1209 | 0·143 | 769 | 0·171 | 538 | 0·229 | 300 | 0·286 | 193 |
| Central | -2·8 (2·2) | 0·273 | 212 | 0·342 | 135 | 0·410 | 94 | 0·547 | 53 | 0·684 | 35 |
| Ventricles | 13·4 (10·1) | 0·244 | 265 | 0·304 | 171 | 0·365 | 119 | 0·487 | 67 | 0·609 | 43 |
| Cerebellum | -1·9 (3·7) | 0·127 | 974 | 0·159 | 622 | 0·191 | 431 | 0·255 | 242 | 0·319 | 155 |
| Medulla | -0·3 (2·5) | 0·003 | 1744192 | 0·004 | 981109 | 0·004 | 981109 | 0·006 | 436049 | 0·007 | 320363 |
| Pons | -0·2 (1·1) | 0·023 | 29675 | 0·029 | 18667 | 0·035 | 12815 | 0·047 | 7107 | 0·059 | 4511 |
| Midbrain | -0·2 (2·1) | 0·006 | 436049 | 0·007 | 320363 | 0·008 | 245278 | 0·011 | 129734 | 0·014 | 80091 |
| Midbrain:Pons | -0·3 (2·5) | 0·012 | 109013 | 0·015 | 69769 | 0·018 | 48451 | 0·024 | 27254 | 0·030 | 17443 |
| **MSA** |  |  |  |  |  |  |  |  |  |  |  |
| Temporal | -3·2 (2·6) | 0·202 | 387 | 0·252 | 248 | 0·303 | 172 | 0·403 | 97 | 0·504 | 63 |
| Cingulate | -3·0 (2·7) | 0·277 | 206 | 0·346 | 132 | 0·416 | 92 | 0·554 | 52 | 0·693 | 34 |
| Insula | -2·3 (3·2) | 0·181 | 480 | 0·226 | 308 | 0·271 | 215 | 0·361 | 121 | 0·451 | 78 |
| Occipital | -2·6 (3·3) | 0·123 | 1039 | 0·153 | 672 | 0·184 | 465 | 0·245 | 262 | 0·307 | 168 |
| Frontal | -4·4 (3·9) | 0·298 | 178 | 0·372 | 114 | 0·446 | 80 | 0·595 | 45 | 0·744 | 29 |
| Parietal | -3·9 (3·4) | 0·268 | 220 | 0·336 | 140 | 0·403 | 98 | 0·537 | 55 | 0·671 | 36 |
| Central | -3·6 (3·0) | 0·377 | 111 | 0·471 | 72 | 0·565 | 50 | 0·753 | 29 | 0·942 | 19 |
| Ventricles | 15·9 (12·3) | 0·219 | 328 | 0·274 | 210 | 0·329 | 146 | 0·439 | 82 | 0·548 | 53 |
| Cerebellum | -5·6 (3·9) | 0·332 | 143 | 0·415 | 92 | 0·497 | 65 | 0·663 | 37 | 0·829 | 24 |
| Medulla | -0·6 (3·0) | 0·027 | 21534 | 0·034 | 13580 | 0·040 | 9812 | 0·054 | 5384 | 0·067 | 3498 |
| Pons | -7·1 (4·5) | 0·310 | 164 | 0·387 | 106 | 0·464 | 74 | 0·619 | 42 | 0·774 | 27 |
| Midbrain | -2·8 (3·6) | 0·237 | 280 | 0·296 | 180 | 0·355 | 126 | 0·473 | 71 | 0·591 | 46 |
| Midbrain:Pons | -0·6 (3·0) | 0·234 | 288 | 0·293 | 184 | 0·352 | 128 | 0·469 | 72 | 0·586 | 47 |
| **IDT** |  |  |  |  |  |  |  |  |  |  |  |
| Temporal | -3·1 (1·6) | 0·602 | 44 | 0·752 | 29 | 0·902 | 20 | 1·203 | 12 | 1·504 | 8 |
| Cingulate | -2·6 (2·0) | 0·447 | 80 | 0·558 | 51 | 0·670 | 36 | 0·893 | 21 | 1·116 | 14 |
| Insula | -1·9 (1·9) | 0·236 | 283 | 0·295 | 181 | 0·354 | 126 | 0·473 | 71 | 0·591 | 46 |
| Occipital | -3·0 (3·8) | 0·169 | 551 | 0·211 | 354 | 0·254 | 244 | 0·338 | 138 | 0·423 | 89 |
| Frontal | -3·7 (1·7) | 0·684 | 35 | 0·855 | 22 | 1·026 | 16 | 1·368 | 9 | 1·710 | 6 |
| Parietal | -2·6 (2·0) | 0·222 | 319 | 0·278 | 204 | 0·333 | 143 | 0·444 | 81 | 0·556 | 52 |
| Central | -1·0 (1·2) | 0·114 | 1209 | 0·142 | 779 | 0·170 | 544 | 0·227 | 306 | 0·284 | 196 |
| Ventricles | 9·2 (6·7) | 0·217 | 334 | 0·271 | 215 | 0·325 | 150 | 0·434 | 84 | 0·542 | 54 |
| Cerebellum | -4·5 (4·0) | 0·247 | 258 | 0·309 | 165 | 0·370 | 116 | 0·494 | 65 | 0·617 | 42 |
| Medulla | 0·8 (1·8) | 0·121 | 1073 | 0·152 | 680 | 0·182 | 475 | 0·243 | 267 | 0·303 | 172 |
| Pons | -0·3 (1·4) | 0·033 | 14416 | 0·041 | 9339 | 0·049 | 6539 | 0·065 | 3716 | 0·082 | 2336 |
| Midbrain | 0·5 (4·5) | 0·038 | 10872 | 0·048 | 6814 | 0·058 | 4667 | 0·077 | 2649 | 0·096 | 1704 |
| Midbrain:Pons | 0·8 (1·8) | 0·067 | 3498 | 0·083 | 2280 | 0·100 | 1571 | 0·133 | 888 | 0·166 | 571 |
| Abbreviations: CBS = corticobasal syndrome, IDT = Indeterminate, MSA = multiple system atrophy, PSP = progressive supranuclear palsy, SD = standard deviation. | | | | | | | | | | | |

| **PSP** |  | **20% change** | | **25% change** | | **30% change** | | **40% change** | | **50% change** | |  |
| --- | --- | --- | --- | --- | --- | --- | --- | --- | --- | --- | --- | --- |
|  | **Difference mean (SD)** | **Effect size** | **Sample size** | **Effect size** | **Sample size** | **Effect size** | **Sample size** | **Effect size** | **Sample size** | **Effect size** | **Sample size** |  |
| **RS** |  |  |  |  |  |  |  |  |  |  |  |  |
| CBD-FS | 11·8 (12·1) | 0·196 | 410 | 0·246 | 260 | 0·295 | 181 | 0·393 | 103 | 0·491 | 66 |  |
| SEADL | -15·9 (19·4) | 0·164 | 585 | 0·206 | 371 | 0·247 | 258 | 0·329 | 146 | 0·411 | 94 |  |
| MS | 1·0 (1·5) | 0·132 | 902 | 0·165 | 578 | 0·198 | 401 | 0·265 | 224 | 0·331 | 144 |  |
| PSP-QoL | 18·4 (22·3) | 0·165 | 578 | 0·206 | 371 | 0·248 | 256 | 0·33 | 145 | 0·413 | 93 |  |
|  |  |  |  |  |  |  |  |  |  |  |  |  |
| UPDRS II | 7·1 (6·1) | 0·231 | 295 | 0·288 | 190 | 0·346 | 132 | 0·461 | 75 | 0·577 | 48 |  |
| UPDRS III | 9·6 (13·7) | 0·139 | 813 | 0·174 | 519 | 0·209 | 360 | 0·279 | 203 | 0·349 | 130 |  |
| PSPRS | 11·6 (9·1) | 0·255 | 242 | 0·319 | 155 | 0·383 | 108 | 0·51 | 61 | 0·638 | 40 |  |
| mPSPRS | 4·0 (3·3) | 0·240 | 273 | 0·300 | 175 | 0·360 | 122 | 0·480 | 69 | 0·600 | 45 |  |
|  |  |  |  |  |  |  |  |  |  |  |  |  |
| UPDRS I | 0·4 (4·0) | 0·019 | 43485 | 0·024 | 27254 | 0·028 | 20024 | 0·038 | 10872 | 0·047 | 7107 |  |
| CBI-R | 15·2 (21·8) | 0·139 | 813 | 0·174 | 519 | 0·209 | 360 | 0·278 | 204 | 0·348 | 131 |  |
| MoCA | -0·6 (3·0) | -0·037 | 11468 | -0·047 | 7107 | -0·056 | 5007 | -0·075 | 2792 | -0·093 | 1816 |  |
| ACE III | -1·4 (5·3) | -0·054 | 5384 | -0·068 | 3396 | -0·081 | 2394 | -0·109 | 1322 | -0·136 | 850 |  |
| ECAS | -4·5 (10·7) | -0·084 | 2226 | -0·105 | 1425 | -0·126 | 990 | -0·167 | 564 | -0·209 | 360 |  |
| FTD-FRS | 4·3 (8·5) | 0·1 | 1571 | 0·125 | 1006 | 0·15 | 699 | 0·2 | 393 | 0·251 | 250 |  |
|  |  |  |  |  |  |  |  |  |  |  |  |  |
| **Cortical** |  |  |  |  |  |  |  |  |  |  |  |  |
| CBD-FS | 15·8 (8·5) | 0·374 | 113 | 0·467 | 73 | 0·56 | 51 | 0·747 | 29 | 0·934 | 19 |  |
| SEADL | -14·5 (7·5) | 0·386 | 106 | 0·482 | 69 | 0·578 | 48 | 0·771 | 27 | 0·964 | 18 |  |
| MS | 0·9 (0·9) | 0·205 | 374 | 0·257 | 239 | 0·308 | 166 | 0·411 | 94 | 0·513 | 61 |  |
| PSP-QoL | 16·7 (19·8) | 0·168 | 557 | 0·211 | 354 | 0·253 | 246 | 0·337 | 139 | 0·421 | 90 |  |
|  |  |  |  |  |  |  |  |  |  |  |  |  |
| UPDRS II | 11·6 (6·6) | 0·355 | 126 | 0·443 | 81 | 0·532 | 56 | 0·709 | 32 | 0·887 | 21 |  |
| UPDRS III | 13·3 (10·3) | 0·259 | 235 | 0·323 | 151 | 0·388 | 105 | 0·517 | 60 | 0·646 | 39 |  |
| PSPRS | 13·9 (8·0) | 0·348 | 131 | 0·435 | 84 | 0·522 | 59 | 0·696 | 33 | 0·871 | 22 |  |
| mPSPRS | 5·8 (2·9) | 0·403 | 98 | 0·504 | 63 | 0·605 | 44 | 0·806 | 25 | 1·008 | 16 |  |
|  |  |  |  |  |  |  |  |  |  |  |  |  |
| UPDRS I | 3·6 (3·3) | 0·221 | 322 | 0·276 | 207 | 0·331 | 144 | 0·441 | 82 | 0·552 | 52 |  |
| CBI-R | 7·3 (12·9) | 0·113 | 1230 | 0·141 | 791 | 0·169 | 551 | 0·225 | 311 | 0·282 | 198 |  |
| MoCA | -3·7 (2·3) | 0·327 | 148 | 0·409 | 95 | 0·491 | 66 | 0·655 | 38 | 0·819 | 24 |  |
| ACE III | -5·2 (6·9) | 0·151 | 689 | 0·189 | 440 | 0·226 | 308 | 0·302 | 173 | 0·377 | 111 |  |
| ECAS | -12·9 (13·6) | 0·19 | 436 | 0·237 | 280 | 0·285 | 194 | 0·379 | 110 | 0·474 | 71 |  |
| FTD-FRS | 4·6 (9·1) | 0·101 | 1540 | 0·126 | 990 | 0·151 | 689 | 0·202 | 386 | 0·252 | 248 |  |
|  |  |  |  |  |  |  |  |  |  |  |  |  |
| **Subcortical** |  |  |  |  |  |  |  |  |  |  |  |  |
| CBD-FS | 8·4 (6·2) | 0·272 | 213 | 0·34 | 137 | 0·408 | 95 | 0·544 | 54 | 0·681 | 35 |  |
| SEADL | -5·2 (25·7) | 0·041 | 9339 | 0·051 | 6036 | 0·061 | 4220 | 0·081 | 2394 | 0·101 | 1540 |  |
| MS | 0·9 (1·2) | 0·156 | 646 | 0·195 | 414 | 0·234 | 288 | 0·312 | 162 | 0·39 | 104 |  |
| PSP-QoL | 9·2 (17·1) | 0·108 | 1347 | 0·135 | 862 | 0·162 | 599 | 0·215 | 341 | 0·269 | 218 |  |
|  |  |  |  |  |  |  |  |  |  |  |  |  |
| UPDRS II | 1·9 (5·7) | 0·067 | 3498 | 0·083 | 2280 | 0·1 | 1571 | 0·133 | 888 | 0·167 | 564 |  |
| UPDRS III | 7·3 (13·2) | 0·111 | 1275 | 0·139 | 813 | 0·167 | 564 | 0·223 | 317 | 0·279 | 203 |  |
| PSPRS | 5·9 (8·0) | 0·149 | 708 | 0·187 | 450 | 0·224 | 314 | 0·299 | 177 | 0·373 | 114 |  |
| mPSPRS | 2·3 (2·6) | 0·176 | 508 | 0·219 | 328 | 0·263 | 228 | 0·351 | 128 | 0·439 | 82 |  |
|  |  |  |  |  |  |  |  |  |  |  |  |  |
| UPDRS I | 0·2 (2·9) | 0·011 | 129734 | 0·014 | 80091 | 0·016 | 61320 | 0·022 | 32434 | 0·027 | 21534 |  |
| CBI-R | -1·1 (15·3) | 0·015 | 69769 | 0·018 | 48451 | 0·022 | 32434 | 0·03 | 17443 | 0·037 | 11468 |  |
| MoCA | -0·6 (4·3) | 0·027 | 21534 | 0·034 | 13580 | 0·041 | 9339 | 0·054 | 5384 | 0·068 | 3396 |  |
| ACE III | -1·1 (4·7) | 0·046 | 7420 | 0·057 | 4833 | 0·069 | 3298 | 0·092 | 1856 | 0·115 | 1188 |  |
| ECAS | 1·4 (15·2) | 0·018 | 48451 | 0·023 | 29675 | 0·027 | 21534 | 0·036 | 12113 | 0·045 | 7753 |  |
| FTD-FRS | -1·7 (8·1) | 0·041 | 9339 | 0·052 | 5806 | 0·062 | 4085 | 0·083 | 2280 | 0·104 | 1452 |  |
|  | | | | | | | | | | | |  |
| Variables with fewer than 5 responses per group have been removed. Abbreviations: ACE III = Addenbrookes Cognitive Examination III, CBD-FS = Corticobasal Degeneration Functional Scale, CBI-R = Cambridge Behavioural Inventory Revised, CBS = corticobasal syndrome , ECAS = Edinburgh Cognitive and Behavioural Screen, FTD-FRS = Frontotemporal Dementia Rating Scale, IDT = Indeterminate, MoCA = Montreal Cognitive Assessment, mPSPRS = modified Progressive Supranuclear Palsy Rating Scale, PSP = progressive supranuclear palsy, PSP QoL = PSP Quality of life scale, PSPRS = PSP rating scale, SD = standard deviation, SEADL = Schwab and England Activities of Daily Living Scale, UPDRS = Unified Parkinson’s Disease Rating Scale | | | | | | | | | | | | |

**Supplementary Table 15: Sample sizes required for a 2-arm, 1-year follow-up therapeutic trial to detect 20%, 25%, 30%, 40% and 50% change by Intention to Treat in PSP phenotypes**

**Supplementary Table 16: Sample sizes required for a 2-arm, 1-year follow-up therapeutic trial to detect 20%, 25%, 30%, 40% and 50% change by Intention to Treat in CBS phenotypes**

| **CBS** |  | **20% change** | | **25% change** | | **30% change** | | **40% change** | | **50% change** | |
| --- | --- | --- | --- | --- | --- | --- | --- | --- | --- | --- | --- |
|  | **Difference mean (SD)** | **Effect size** | **Sample size** | **Effect size** | **Sample size** | **Effect size** | **Sample size** | **Effect size** | **Sample size** | **Effect size** | **Sample size** |
| **4RT** |  |  |  |  |  |  |  |  |  |  |  |
| SEADL | -14·7 (10·3) | 0·284 | 196 | 0·355 | 126 | 0·426 | 87 | 0·568 | 50 | 0·709 | 32 |
| Milestones | 0·9 (1·6) | 0·114 | 1209 | 0·143 | 769 | 0·172 | 532 | 0·229 | 300 | 0·286 | 193 |
| PSP-QoL | 27·9 (23·9) | 0·233 | 290 | 0·292 | 185 | 0·350 | 129 | 0·467 | 73 | 0·584 | 47 |
|  |  |  |  |  |  |  |  |  |  |  |  |
| UPDRS II | 10·3 (7·4) | 0·279 | 203 | 0·349 | 130 | 0·418 | 91 | 0·558 | 51 | 0·697 | 33 |
| UPDRS III | 10·0 (10·3) | 0·194 | 418 | 0·242 | 269 | 0·291 | 186 | 0·388 | 105 | 0·484 | 68 |
| PSPRS | 7·1 (9·7) | 0·146 | 737 | 0·183 | 470 | 0·219 | 328 | 0·292 | 185 | 0·366 | 118 |
| mPSPRS | 3·2 (2·5) | 0·253 | 246 | 0·316 | 158 | 0·379 | 110 | 0·506 | 62 | 0·632 | 40 |
|  |  |  |  |  |  |  |  |  |  |  |  |
| UPDRS I | 2·8 (3·4) | 0·165 | 578 | 0·206 | 371 | 0·248 | 256 | 0·330 | 145 | 0·413 | 93 |
| CBI-R | 16·2 (14·7) | 0·221 | 322 | 0·276 | 207 | 0·331 | 144 | 0·442 | 81 | 0·552 | 52 |
| MoCA | -1·6 (2·7) | 0·117 | 1148 | 0·146 | 737 | 0·176 | 508 | 0·234 | 288 | 0·293 | 184 |
| ACE III | -6·8 (8·7) | 0·156 | 646 | 0·195 | 414 | 0·234 | 288 | 0·312 | 162 | 0·390 | 104 |
| ECAS | 2·3 (10·4) | 0·045 | 7753 | 0·056 | 5007 | 0·068 | 3396 | 0·090 | 1939 | 0·113 | 1230 |
| FTD-FRS | -1·5 (15·8) | 0·020 | 39245 | 0·024 | 27254 | 0·029 | 18667 | 0·039 | 10322 | 0·049 | 6539 |
|  |  |  |  |  |  |  |  |  |  |  |  |
| **AD** |  |  |  |  |  |  |  |  |  |  |  |
| SEADL | -11·7 (12·8) | 0·182 | 475 | 0·227 | 306 | 0·272 | 213 | 0·363 | 120 | 0·454 | 77 |
| Milestones | 0·4 (0·5) | 0·149 | 708 | 0·186 | 455 | 0·223 | 317 | 0·298 | 178 | 0·372 | 114 |
| PSP-QoL | 8·0 (14·4) | 0·111 | 1275 | 0·139 | 813 | 0·167 | 564 | 0·222 | 319 | 0·278 | 204 |
|  |  |  |  |  |  |  |  |  |  |  |  |
| UPDRS II | 5·1 (4·8) | 0·209 | 360 | 0·261 | 231 | 0·314 | 160 | 0·418 | 91 | 0·523 | 58 |
| UPDRS III | 6·1 (7·6) | 0·160 | 614 | 0·200 | 393 | 0·240 | 273 | 0·321 | 153 | 0·401 | 99 |
| PSPRS | 7·6 (5·4) | 0·281 | 200 | 0·351 | 128 | 0·422 | 89 | 0·562 | 51 | 0·703 | 33 |
| mPSPRS | 2·1 (2·4) | 0·173 | 525 | 0·216 | 337 | 0·260 | 233 | 0·346 | 132 | 0·433 | 85 |
|  |  |  |  |  |  |  |  |  |  |  |  |
| UPDRS I | 1·5 (3·0) | 0·098 | 1635 | 0·122 | 1056 | 0·147 | 727 | 0·196 | 410 | 0·245 | 262 |
| CBI-R | 6·6 (12·0) | 0·110 | 1298 | 0·138 | 825 | 0·165 | 578 | 0·221 | 322 | 0·276 | 207 |
| MoCA | -2·3 (3·1) | 0·151 | 689 | 0·189 | 440 | 0·227 | 306 | 0·302 | 173 | 0·378 | 111 |
| ACE III | -11·8 (10·0) | 0·235 | 285 | 0·293 | 184 | 0·352 | 128 | 0·469 | 72 | 0·586 | 47 |
| ECAS | -4·5 (10·7) | 0·084 | 2226 | 0·105 | 1425 | 0·126 | 990 | 0·167 | 564 | 0·209 | 360 |
| FTD-FRS | 5·9 (6·4) | 0·185 | 460 | 0·231 | 295 | 0·277 | 206 | 0·370 | 116 | 0·462 | 75 |
|  |  |  |  |  |  |  |  |  |  |  |  |
| **IDT** |  |  |  |  |  |  |  |  |  |  |  |
| SEADL | -12·2 (13·9) | 0·176 | 508 | 0·220 | 325 | 0·264 | 226 | 0·353 | 127 | 0·441 | 82 |
| Milestones | 0·4 (0·9) | 0·086 | 2123 | 0·108 | 1347 | 0·130 | 930 | 0·173 | 525 | 0·216 | 337 |
| PSP-QoL | 11·5 (19·0) | 0·121 | 1073 | 0·151 | 689 | 0·181 | 480 | 0·242 | 269 | 0·302 | 173 |
|  |  |  |  |  |  |  |  |  |  |  |  |
| UPDRS II | 7·1 (6·5) | 0·218 | 331 | 0·273 | 212 | 0·328 | 147 | 0·437 | 83 | 0·546 | 54 |
| UPDRS III | 5·0 (8·7) | 0·114 | 1209 | 0·142 | 779 | 0·171 | 538 | 0·227 | 306 | 0·284 | 196 |
| PSPRS | 9·4 (8·5) | 0·221 | 322 | 0·277 | 206 | 0·332 | 143 | 0·443 | 81 | 0·554 | 52 |
| mPSPRS | 2·7 (3·1) | 0·174 | 519 | 0·218 | 331 | 0·261 | 231 | 0·348 | 131 | 0·436 | 84 |
|  |  |  |  |  |  |  |  |  |  |  |  |
| UPDRS I | 0·5 (2·6) | 0·038 | 10872 | 0·047 | 7107 | 0·057 | 4833 | 0·075 | 2792 | 0·094 | 1778 |
| CBI-R | 16·4 (26·3) | 0·125 | 1006 | 0·156 | 646 | 0·187 | 450 | 0·250 | 252 | 0·312 | 162 |
| MoCA | -0·8 (3·6) | 0·047 | 7107 | 0·059 | 4511 | 0·071 | 3115 | 0·095 | 1740 | 0·119 | 1109 |
| ACE III | -4·3 (6·5) | 0·132 | 902 | 0·165 | 578 | 0·198 | 401 | 0·265 | 224 | 0·331 | 144 |
| ECAS | -7·3 (10·8) | 0·135 | 862 | 0·169 | 551 | 0·203 | 382 | 0·271 | 215 | 0·338 | 138 |
| FTD-FRS | 3·0 (9·5) | 0·064 | 3833 | 0·080 | 2454 | 0·095 | 1740 | 0·127 | 974 | 0·159 | 622 |
|  | | | | | | | | | | | |
| Variables with fewer than 5 responses per group have been removed. Abbreviations: 4RT = 4-repeat tau, AD = Alzheimer disease, ACE III = Addenbrookes Cognitive Examination III, CBD-FS = Corticobasal Degeneration Functional Scale, CBI-R = Cambridge Behavioural Inventory Revised, CBS = corticobasal syndrome , ECAS = Edinburgh Cognitive and Behavioural Screen, FTD-FRS = Frontotemporal Dementia Rating Scale, IDT = Indeterminate, MoCA = Montreal Cognitive Assessment, mPSPRS = modified Progressive Supranuclear Palsy Rating Scale, MSA = multiple system atrophy, MSA QoL = MSA Quality of life scale, PSP = progressive supranuclear palsy, PSP QoL = PSP Quality of life scale, PSPRS = PSP rating scale, SD = standard deviation, SEADL = Schwab and England Activities of Daily Living Scale, UPDRS = Unified Parkinson’s Disease Rating Scale, UMSARS = Unified MSA Rating Scale | | | | | | | | | | | |

**Supplementary Table 17: Sample sizes required for a 2-arm, 1-year follow-up therapeutic trial to detect 20%, 25%, 30%, 40% and 50% change by Intention to Treat in MSA phenotypes**

| **MSA** |  | **20% change** | | **25% change** | | **30% change** | | **40% change** | | **50% change** | |
| --- | --- | --- | --- | --- | --- | --- | --- | --- | --- | --- | --- |
|  | **Difference mean (SD)** | **Effect size** | **Sample size** | **Effect size** | **Sample size** | **Effect size** | **Sample size** | **Effect size** | **Sample size** | **Effect size** | **Sample size** |
| **MSA-C** |  |  |  |  |  |  |  |  |  |  |  |
| SEADL | -10·4 (19·0) | 0·109 | 1322 | 0·136 | 850 | 0·164 | 585 | 0·218 | 331 | 0·273 | 212 |
| MS | 0·6 (0·9) | 0·137 | 837 | 0·171 | 538 | 0·205 | 374 | 0·274 | 210 | 0·342 | 135 |
| MSA-QoL | 9·8 (13·8) | 0·141 | 791 | 0·177 | 502 | 0·212 | 350 | 0·282 | 198 | 0·353 | 127 |
|  |  |  |  |  |  |  |  |  |  |  |  |
| UPDRS II | 5·6 (9·1) | 0·123 | 1039 | 0·153 | 672 | 0·184 | 465 | 0·245 | 262 | 0·307 | 168 |
| UMSARS | 7·0 (7·4) | 0·19 | 436 | 0·237 | 280 | 0·284 | 196 | 0·379 | 110 | 0·474 | 71 |
|  |  |  |  |  |  |  |  |  |  |  |  |
| UPDRS I | 1·2 (4·3) | 0·056 | 5007 | 0·069 | 3298 | 0·083 | 2280 | 0·111 | 1275 | 0·139 | 813 |
| CBI-R | 5·6 (21·0) | 0·053 | 5589 | 0·066 | 3605 | 0·08 | 2454 | 0·106 | 1398 | 0·133 | 888 |
| MoCA | 0·7 (3·7) | 0·039 | 10322 | 0·049 | 6539 | 0·059 | 4511 | 0·079 | 2516 | 0·098 | 1635 |
| ACE III | -2·2 (6·6) | 0·067 | 3498 | 0·083 | 2280 | 0·1 | 1571 | 0·133 | 888 | 0·166 | 571 |
| ECAS | -6·2 (12·7) | 0·098 | 1635 | 0·123 | 1039 | 0·148 | 718 | 0·197 | 405 | 0·246 | 260 |
| FTD-FRS | 1·0 (9·2) | 0·021 | 35597 | 0·026 | 23222 | 0·031 | 16336 | 0·042 | 8900 | 0·052 | 5806 |
|  |  |  |  |  |  |  |  |  |  |  |  |
| **MSA-P** |  |  |  |  |  |  |  |  |  |  |  |
| SEADL | -7·2 (10·8) | 0·133 | 888 | 0·166 | 571 | 0·199 | 397 | 0·265 | 224 | 0·332 | 143 |
| MS | 0·7 (1·1) | 0·131 | 916 | 0·163 | 592 | 0·196 | 410 | 0·262 | 230 | 0·327 | 148 |
| MSA-QoL | -1·9 (13·2) | 0·028 | 20024 | 0·035 | 12815 | 0·042 | 8900 | 0·057 | 4833 | 0·071 | 3115 |
|  |  |  |  |  |  |  |  |  |  |  |  |
| UPDRS II | 3·4 (4·5) | 0·151 | 689 | 0·189 | 440 | 0·227 | 306 | 0·302 | 173 | 0·378 | 111 |
| UMSARS | 11·2 (8·6) | 0·261 | 231 | 0·326 | 149 | 0·392 | 103 | 0·522 | 59 | 0·653 | 38 |
|  |  |  |  |  |  |  |  |  |  |  |  |
| UPDRS I | -0·8 (3·8) | 0·043 | 8491 | 0·054 | 5384 | 0·064 | 3833 | 0·086 | 2123 | 0·107 | 1372 |
| CBI-R | 17·8 (22·5) | 0·158 | 630 | 0·198 | 401 | 0·237 | 280 | 0·316 | 158 | 0·395 | 102 |
| MoCA | -0·2 (2·6) | 0·013 | 92887 | 0·016 | 61320 | 0·02 | 39245 | 0·026 | 23222 | 0·033 | 14416 |
| ACE III | -0·8 (7·1) | 0·022 | 32434 | 0·028 | 20024 | 0·033 | 14416 | 0·045 | 7753 | 0·056 | 5007 |
| ECAS | -3·6 (9·4) | 0·076 | 2719 | 0·095 | 1740 | 0·114 | 1209 | 0·152 | 680 | 0·19 | 436 |
| FTD-FRS | 1·8 (5·4) | 0·066 | 3605 | 0·082 | 2336 | 0·098 | 1635 | 0·131 | 916 | 0·164 | 585 |
|  | | | | | | | | | | | |
| Variables with fewer than 5 responses per group have been removed. Abbreviations: ACE III = Addenbrookes Cognitive Examination III, CBI-R = Cambridge Behavioural Inventory Revised, ECAS = Edinburgh Cognitive and Behavioural Screen, FTD-FRS = Frontotemporal Dementia Rating Scale, MoCA = Montreal Cognitive Assessment, MSA = multiple system atrophy, MSA-C = MSA cerebellar variant, MSA-P = MSA parkinsonism variant, MSA QoL = MSA Quality of life scale, SD = standard deviation, SEADL = Schwab and England Activities of Daily Living Scale, UPDRS = Unified Parkinson’s Disease Rating Scale, UMSARS = Unified MSA Rating Scale | | | | | | | | | | | |

**Supplementary Table 18: Sample sizes required for a 2-arm, 1-year follow-up therapeutic trial to detect 20%, 25%, 30%, 40% and 50% change by Intention to Treat according to disease group**

| **IMAGING** |  | **20% change** | | **25% change** | | **30% change** | | **40% change** | | **50% change** | |
| --- | --- | --- | --- | --- | --- | --- | --- | --- | --- | --- | --- |
|  | **Percent difference mean (SD)** | **Effect size** | **Sample size** | **Effect size** | **Sample size** | **Effect size** | **Sample size** | **Effect size** | **Sample size** | **Effect size** | **Sample size** |
| **PSP** |  |  |  |  |  |  |  |  |  |  |  |
| Temporal | -2·8 (2·3) | 0·215 | 341 | 0·269 | 218 | 0·323 | 151 | 0·43 | 86 | 0·538 | 55 |
| Cingulate | -2·0 (2·1) | 0·207 | 367 | 0·259 | 235 | 0·311 | 163 | 0·414 | 93 | 0·518 | 59 |
| Insula | -1·3 (2·5) | 0·124 | 1022 | 0·156 | 646 | 0·187 | 450 | 0·249 | 254 | 0·311 | 163 |
| Occipital | -2·7 (2·8) | 0·167 | 564 | 0·208 | 364 | 0·250 | 252 | 0·333 | 143 | 0·417 | 91 |
| Frontal | -3·8 (3·0) | 0·272 | 213 | 0·341 | 136 | 0·409 | 95 | 0·545 | 54 | 0·681 | 35 |
| Parietal | -3·0 (2·9) | 0·233 | 290 | 0·291 | 186 | 0·350 | 129 | 0·466 | 73 | 0·583 | 47 |
| Central | -1·9 (3·4) | 0·097 | 1669 | 0·121 | 1073 | 0·146 | 737 | 0·194 | 418 | 0·243 | 267 |
| Ventricles | 10·7 (6·7) | 0·337 | 139 | 0·422 | 89 | 0·506 | 62 | 0·675 | 35 | 0·843 | 23 |
| Cerebellum | -3·7 (4·3) | 0·189 | 440 | 0·236 | 283 | 0·284 | 196 | 0·378 | 111 | 0·473 | 71 |
| Medulla | 1·0 (7·0) | 0·022 | 32434 | 0·028 | 20024 | 0·033 | 14416 | 0·044 | 8109 | 0·055 | 5190 |
| Pons | -2·2 (3·0) | 0·187 | 450 | 0·234 | 288 | 0·281 | 200 | 0·374 | 113 | 0·468 | 73 |
| Midbrain | -0·5 (4·8) | 0·023 | 29675 | 0·029 | 18667 | 0·034 | 13580 | 0·046 | 7420 | 0·057 | 4833 |
| Midbrain:Pons | 1·0 (7·0) | 0·089 | 1983 | 0·111 | 1275 | 0·133 | 888 | 0·178 | 496 | 0·222 | 319 |
| **CBS** |  |  |  |  |  |  |  |  |  |  |  |
| Temporal | -1·8 (4·0) | 0·134 | 875 | 0·167 | 564 | 0·2 | 393 | 0·267 | 221 | 0·334 | 142 |
| Cingulate | -2·4 (2·9) | 0·212 | 350 | 0·265 | 224 | 0·318 | 156 | 0·424 | 88 | 0·53 | 57 |
| Insula | -1·5 (3·2) | 0·131 | 916 | 0·164 | 585 | 0·197 | 405 | 0·262 | 230 | 0·328 | 147 |
| Occipital | -1·1 (4·4) | 0·063 | 3956 | 0·079 | 2516 | 0·095 | 1740 | 0·127 | 974 | 0·159 | 622 |
| Frontal | -3·0 (4·4) | 0·191 | 431 | 0·238 | 278 | 0·286 | 193 | 0·382 | 109 | 0·477 | 70 |
| Parietal | -2·6 (5·9) | 0·137 | 837 | 0·171 | 538 | 0·206 | 371 | 0·274 | 210 | 0·343 | 134 |
| Central | -3·1 (2·2) | 0·314 | 160 | 0·393 | 103 | 0·471 | 72 | 0·628 | 41 | 0·785 | 26 |
| Ventricles | 13·3 (9·2) | 0·310 | 164 | 0·388 | 105 | 0·465 | 74 | 0·620 | 42 | 0·776 | 27 |
| Cerebellum | -2·1 (3·7) | 0·159 | 622 | 0·199 | 397 | 0·239 | 276 | 0·318 | 156 | 0·398 | 100 |
| Medulla | 0·3 (2·7) | 0·060 | 4361 | 0·075 | 2792 | 0·09 | 1939 | 0·120 | 1091 | 0·15 | 699 |
| Pons | -2·4 (4·7) | 0·150 | 699 | 0·187 | 450 | 0·225 | 311 | 0·300 | 175 | 0·374 | 113 |
| Midbrain | -1·2 (2·9) | 0·095 | 1740 | 0·119 | 1109 | 0·143 | 769 | 0·190 | 436 | 0·238 | 278 |
| Midbrain:Pons | 0·3 (2·7) | 0·128 | 959 | 0·160 | 614 | 0·192 | 427 | 0·256 | 240 | 0·32 | 154 |
| **MSA** |  |  |  |  |  |  |  |  |  |  |  |
| Temporal | -3·3 (2·8) | 0·205 | 374 | 0·256 | 240 | 0·307 | 168 | 0·409 | 95 | 0·512 | 61 |
| Cingulate | -2·8 (2·8) | 0·259 | 235 | 0·324 | 151 | 0·388 | 105 | 0·518 | 59 | 0·647 | 38 |
| Insula | -2·2 (3·4) | 0·171 | 538 | 0·214 | 344 | 0·256 | 240 | 0·342 | 135 | 0·427 | 87 |
| Occipital | -3·0 (3·3) | 0·141 | 791 | 0·177 | 502 | 0·212 | 350 | 0·283 | 197 | 0·354 | 126 |
| Frontal | -4·2 (4·1) | 0·276 | 207 | 0·345 | 133 | 0·414 | 93 | 0·552 | 52 | 0·690 | 34 |
| Parietal | -3·9 (3·6) | 0·267 | 221 | 0·334 | 142 | 0·4 | 99 | 0·534 | 56 | 0·667 | 36 |
| Central | -3·3 (3·0) | 0·340 | 137 | 0·425 | 88 | 0·51 | 61 | 0·679 | 35 | 0·849 | 23 |
| Ventricles | 15·5 (13·0) | 0·195 | 414 | 0·244 | 265 | 0·292 | 185 | 0·390 | 104 | 0·487 | 67 |
| Cerebellum | -5·9 (4·0) | 0·357 | 124 | 0·447 | 80 | 0·536 | 56 | 0·714 | 32 | 0·893 | 21 |
| Medulla | -1·0 (3·0) | 0·056 | 5007 | 0·070 | 3205 | 0·084 | 2226 | 0·112 | 1252 | 0·140 | 802 |
| Pons | -6·7 (4·7) | 0·271 | 215 | 0·339 | 138 | 0·407 | 96 | 0·542 | 54 | 0·678 | 35 |
| Midbrain | -2·6 (3·7) | 0·223 | 317 | 0·279 | 203 | 0·335 | 141 | 0·447 | 80 | 0·558 | 51 |
| Midbrain:Pons | -1·0 (3·0) | 0·205 | 374 | 0·257 | 239 | 0·308 | 166 | 0·411 | 94 | 0·514 | 60 |
| **IDT** |  |  |  |  |  |  |  |  |  |  |  |
| Temporal | -2·8 (1·3) | 0·769 | 28 | 0·961 | 18 | 1·153 | 13 | 1·537 | 8 | 1·922 | 5 |
| Cingulate | -2·9 (1·9) | 0·403 | 98 | 0·504 | 63 | 0·605 | 44 | 0·806 | 25 | 1·008 | 16 |
| Insula | -1·3 (1·2) | 0·222 | 319 | 0·278 | 204 | 0·333 | 143 | 0·445 | 80 | 0·556 | 52 |
| Occipital | -2·0 (2·1) | 0·171 | 538 | 0·213 | 347 | 0·256 | 240 | 0·341 | 136 | 0·427 | 87 |
| Frontal | -3·3 (1·9) | 0·400 | 99 | 0·500 | 64 | 0·6 | 45 | 0·800 | 26 | 1·001 | 17 |
| Parietal | -2·2 (1·7) | 0·207 | 367 | 0·258 | 237 | 0·31 | 164 | 0·413 | 93 | 0·516 | 60 |
| Central | -2·3 (2·8) | 0·149 | 708 | 0·186 | 455 | 0·223 | 317 | 0·298 | 178 | 0·372 | 114 |
| Ventricles | 13·2 (9·1) | 0·250 | 252 | 0·312 | 162 | 0·375 | 113 | 0·500 | 64 | 0·625 | 41 |
| Cerebellum | -3·0 (1·6) | 0·388 | 105 | 0·485 | 68 | 0·582 | 47 | 0·776 | 27 | 0·971 | 18 |
| Medulla | 0·4 (1·8) | 0·059 | 4511 | 0·074 | 2868 | 0·089 | 1983 | 0·118 | 1128 | 0·148 | 718 |
| Pons | -0·4 (1·5) | 0·005 | 627910 | 0·006 | 436049 | 0·007 | 320363 | 0·009 | 193800 | 0·012 | 109013 |
| Midbrain | 0·3 (4·5) | 0·031 | 16336 | 0·039 | 10322 | 0·047 | 7107 | 0·063 | 3956 | 0·078 | 2581 |
| Midbrain:Pons | 0·4 (1·8) | 0·067 | 3498 | 0·084 | 2226 | 0·101 | 1540 | 0·134 | 875 | 0·168 | 557 |
| Abbreviations: CBS = corticobasal syndrome, IDT = Indeterminate, MSA = multiple system atrophy, PSP = progressive supranuclear palsy, SD = standard deviation. | | | | | | | | | | | |

**Supplementary Table 19:** **Inclusion and Exclusion Criteria for PSP Trial Involvement, applied in the current study**

| **Inclusion criteria** | |
| --- | --- |
| **Type of participant and target disease characteristics:** | a) Probable or possible PSP as defined as:  i)   1. A progressive history of postural instability during the first 3 years that symptoms are present,   OR   1. A progressive history of falls during the first 3 years that symptoms are present;   ii)   1. Vertical supranuclear gaze palsy defined as clear limitation of the range of voluntary gaze in the vertical more than in the horizontal plane, more than expected with age, which is overcome by activation with the vestibulo-ocular reflex,   OR   1. Slow velocity of vertical saccades (i.e. decreased velocity (and gain) of vertical greater than horizontal saccadic eye movements);   iii) Age at symptom onset of 40 to 85 years by history and current age between 41 and 86 years, inclusive, at time of screening;  iv) An akinetic-rigid syndrome; and  v) Present of PSP symptoms for less than or equal to 5 years (determined by the best judgement of the investigator) at screening. |
|  | b) Body weight range of ≥43 kg/95 lbs to ≤ 120kg/265 lbs. |
|  | c) Able to ambulate independently or with assistance defined as the ability to take at least 10 steps with a walker or the ability to take at least 10 steps without a walker or cane with the assistance of another person who can only have contact with one upper extremity. |
|  | d) Able to tolerate MRI. |
|  | e) Able to perform all protocol-specific assessments and comply with the study visit schedule. |
|  | f) Able to comply with neuropsychological evaluation at screening and throughout the 52-week double-blind period of the study. |
|  | g) Have reliable caregiver to accompany participant to all study visits. |
|  | h) Score ≥20 on the Mini Mental State Examination (MMSE) at screening. |
|  | i) Participant must reside outside a skilled nursing facility or dementia care facility at the time of screening and admission to such a facility must not be planned. |
|  | j) If participant is receiving coenzyme Q10, levodopa/carbidopa, levodopa/benserazide, fava bean extract, a dopamine agonist, catechol-0-methyltransferase inhibitor, amantadine, or other Parkinson’s disease medications, the dose must have been stable for at least 60 days prior to screening and expected to remain stable for the double-blind period of the study. |
|  | k) Stable on other chronic medications for at least 30 days prior to screening. |
| **Age and reproductive status:** | a) Males and Females, ages 41 to 86 years inclusive. |
|  | b) Women of childbearing potential must have a negative serum or urine pregnancy test within 24 hours prior to the start of study treatment. |
|  | c) Women must not be breastfeeding. |
|  | d) Women of childbearing potential must agree to follow instructions for method(s) of contraception for the duration of treatment with study treatment(s). |
|  | e) Males who are sexually active must agree to follow instructions for method(s) of contraception for the duration of treatment with study treatment(s). |

| **Exclusion criteria** | |
| --- | --- |
| **Medical conditions** | a) History of clinically significant haematological, endocrine, cardiovascular, renal, hepatic, gastrointestinal, psychiatric, or neurological disease. |
|  | b) History of or screening brain MRI scan indicative of significant abnormality. |
|  | c) Known presence of disease-associated genetic mutation. |
|  | d) Any major surgery within 4 weeks of screening. |
|  | e) History of deep brain stimulator surgery other than sham surgery for deep brain stimulation (DBS) clinical trial. |
|  | f) Inability to be venepunctured and/or tolerate venous access. |
|  | g) Contraindication to the MRI examination for any reason. |
|  | h) History of cancer within 5 years of screening. |
|  | i) Blood transfusion within 4 weeks of screening. |
|  | j) Any vaccination within 30 days prior to screening. |
|  | k) Known history of human immunodeficiency virus infection. |
| **Prior/Concomitant therapy** | a) Concurrent treatment with memantine; acetylcholinesterase inhibitors, antipsychotic agents, or mood stabilizers (e.g., valproate, lithium); or benzodiazepines. |
|  | b) Receipt of systemic corticosteroids within 30 days prior to screening. |
|  | c) Receipt of an investigational immunomodulator or monoclonal antibody within 180 days (or 5 half-lives, whichever is longer) prior to screening. |
|  | d) Treatment with any other investigational drugs including placebo or devices within 90 days prior to screening. |
| **Physical and Laboratory Test Findings** | a) Evidence of organ dysfunction or any clinically significant deviation from normal in physical examination, vital signs, ECG, or clinical laboratory determinations. |
|  | b) Clinically significant abnormality on 12-lead ECG prior to study drug administration, confirmed by repeat. |
|  | c) Total bilirubin, alanine aminotransferase (ALT) or aspartate aminotransferase (AST) greater than 2 times the upper limit of normal (ULN), confirmed by repeat. |
|  | d) Serum creatinine >168 µmol/L (1.9 mg/dL), confirmed by repeat. |
|  | e) Haematocrit less than 35% for males and less than 32% for females, absolute neutrophil cell count of ≤1500/µL (with the exception of a documented history of a chronic benign neutropenia), or platelet cell count of <120,000/µL; INR >1.4 or other coagulopathy, confirmed by repeat. |
|  | f) A clinically significant abnormal thyroid stimulating hormone (TSH) test. |
|  | g) For those participating in the CSF substudy, an abnormally increased number of white blood cells (>7 cells/mm3) in the CSF obtained at the screening visit; if there is evidence that the spinal tap was traumatic, participants with >7 cells/mm3 must be discussed with the Medical Monitor to determine if they may be eligible. |
|  | h) Haemoglobin A1C >7.5%, confirmed by repeat. |
|  | i) Positive blood screen for hepatitis C antibody, hepatitis B surface antigen. |
| **Allergies and Adverse Drug Reaction** | a) History of allergy, hypersensitivity, or serious adverse reaction to monoclonal antibodies or related compounds. |
|  | b) Allergy to any of the components of the study drug. |
|  | c) History of any significant drug allergy (such as anaphylaxis or hepatotoxicity). |
| **Other exclusion criteria** | a) Prisoners or participants who are involuntarily incarcerated. |
|  | b) Participants who are compulsorily detained for treatment of either a psychiatric or physical (e.g., infectious disease) illness |

**Supplementary Table 20: Inclusion and Exclusion Criteria for MSA Trial Involvement, applied in the current study**

| **Inclusion criteria** |
| --- |
| Male and female subjects between the ages of ≥ 40 to ≤ 80 years at time of Screening. |
| Subjects must provide a written signed and dated informed consent form/forms (IRB/EC specific) in accordance with regulatory and institutional guidelines, prior to the initiation of any protocol required procedures. Only patients with the capacity to understand the nature, significance and scope of the clinical trial interventions and to express their wishes accordingly may provide consent to participate in the study. |
| Caregivers must be willing to sign and date an IRB/EC-approved written informed consent form that outlines the caregiver expectations and responsibilities in this study, in accordance with regulatory and institutional guidelines, as appropriate.   - Diagnosis of probable or possible MSA according to consensus clinical criteria (Gilman et al 2008), including subjects with MSA of either subtype (MSA-P or MSA-C); - Able to ambulate without the assistance of another person, defined as the ability to take at least 10 steps. Use of assistive devices (e.g., walker or cane) is allowed; - Anticipated survival of at least 3 years at the time of Screening, as judged by the Investigator. d. A brain MRI scan (conducted within the 14 days prior to Baseline/Day 1, approximately) that does not rule out a diagnosis of MSA; - Able to tolerate MRI; - Body mass index (BMI) < 40 kg/m^2 at Screening; - Able to swallow tablets whole and anticipated to be able to do so throughout the duration of the study; - Willing and able to adhere to the study drug regimen; - Willing and able to perform all protocol-specified assessments and comply with the study visit schedule; - Able to read, understand, and speak local language fluently to ensure comprehension of informed consent and protocol-specified assessments; - Must have reliable caregiver to accompany subject to study visits, with the same caregiver completing caregiver assessments at Baseline, Week 24 and Week 48/Early  Discontinuation, when possible. Caregiver must be able to read, understand, and speak local language fluently to ensure comprehension of informed consent and protocol specified assessments. Caregiver must also have frequent contact with subject (at least 3 hours per week at one time or different times) and be willing to monitor the subject's health and concomitant medications throughout the study; - If subject is receiving treatment for MSA, the doses must have been stable for at least 30 days prior to Baseline/Randomization and medications present at Baseline expected to remain relatively stable during the study period. This may include medications commonly used for Parkinson's disease (e.g., coenzyme Q10, levodopa/ carbidopa, levodopa/ benserazide, fava bean extract, a dopamine agonist, catechol-0-methyltransferase inhibitor, amantadine) or those for autonomic dysfunction (e.g., ephedrine, midodrine, fludrocortisone, octreotide, desmopressin, oxybutynine, droxidopa); - Stable on other chronic medications and supplements for at least 30 days prior to Baseline/Randomization and expected to remain relatively stable during the study period; - Women of child bearing potential (WOCBP) and fertile men (including those vasectomized for less than 6 months) with female partners who are WOCBP (not surgically sterile and not postmenopausal) must agree to use highly effective birth control, including two methods of contraception, for the duration of the study (i.e., beginning 30 days prior to Baseline/Randomization and extending to 30 days for women and 90 days for men after the last dose of study drug); - The two methods of contraception should include: - one barrier method (e.g., diaphragm with spermicide, condom with spermicidal gel, cervical cap); - and one other method that could include hormonal contraceptives (e.g., oral contraceptives, injectable contraceptives, contraceptive implant, patch) used for at least 4 weeks prior to sexual intercourse; - WOCBP must have a negative serum pregnancy test at Screening and a negative urine pregnancy test within approximately 24 hours prior to dosing at Baseline/ Randomization. |

| **Exclusion criteria** |
| --- |
| Subjects having advanced disease, defined by the presence of one or more of the following on the UMSARS Part I:   - Speech impairment, as assessed by a score greater than or equal to 3, Question 1; - Swallowing impairment, as assessed by a score of greater than or equal to 3 on Question 2; - Falling more frequently than once per week, as assessed by a score of greater than or equal to 3 on Question 8; or Subjects having significant cognitive impairment, defined by a score of less than or equal to 20 on the MoCA (score includes any limitation due to motor impairments). |
| Any condition that would interfere with the subject’s ability to comply with study instructions, place the subject at unacceptable risk, and/or confound the interpretation of safety or efficacy data from the study, as judged by the Investigator. |
| Diagnosis of neurological disorders, other than MSA, including (but not limited to) the following:   - Idiopathic Parkinson's disease or another form of parkinsonism (e.g., dementia with Lewy bodies, drug-induced, post-encephalitic, vascular), which has not subsequently revised to a diagnosis of MSA; - Any other neurodegenerative disease (e.g., Alzheimer's disease, frontotemporal dementia, amyotrophic lateral sclerosis, prion disease); - Any other clinically significant neurological disorder (e.g., history of stroke, history of head injury with loss of consciousness for at least 15 minutes within the past 20 years, history of seizure disorder, brain tumor, or other space-occupying lesion). |
| History of or Screening brain MRI scan indicative of significant abnormality, including (but not limited to) the following: prior hemorrhage or infarct greater than 1 cm3; 3 or more lacunar infarcts; cerebral contusion; encephalomalacia; aneurysm; vascular malformation; subdural hematoma; hydrocephalus; space-occupying lesion (e.g., abscess or brain tumor). |
| Contraindication to MRI examination for any reason |
| For those participating in the optional CSF sub-study, contraindication to undergoing an LP including, but not limited to: inability to tolerate an appropriately flexed position for the time necessary to perform an LP; infection at the desired LP site; taking antiplatelet/anticoagulant medications that cannot be discontinued for a short period of time prior to performing the LP; degenerative arthritis of the lumbar spine; suspected non-communicating hydrocephalus or intracranial mass; prior history of spinal mass or trauma. |
| Presence of clinically significant thyroid disease with abnormal free T4 levels and TSH >10 mIU/L (despite treatment) at Screening, confirmed by repeat. |
| Within 1 year prior to Screening or between Screening and Baseline (Day -1), any of the following: myocardial infarction; hospitalization for congestive heart failure; hospitalization for, or symptoms of, unstable angina; or syncope not related to MSA. |
| Diagnosis of clinically significant psychiatric disorder including (but not limited to) the following: any psychotic disorder, severe bipolar or unipolar depression, prior history of suicidal thoughts or behavior that are believed to represent a current safety risk. |
| History of substance use disorder (drug or alcohol) in the last 12 months, with the exception of nicotine, as defined by DSM-V criteria. |
| History or presence of gastrointestinal or other disease known to interfere with absorption, distribution, metabolism, or excretion of drugs, or a history of surgery known to interfere with absorption or excretion of drugs (i.e., gastric bypass). |
| History of any other clinically significant disease (e.g., autoimmune, cardiovascular, endocrine, gastrointestinal, hematological, hepatic, immunological, infectious, neurological, pulmonary, psychiatric, or renal) that, based on the judgment of the Investigator, is clinically unstable, is likely to deteriorate during the course of the study, could put the patient at risk because of participation in the study, could affect the subject’s ability to complete the study, or could influence the study results. |
| History of human immunodeficiency virus infection. |
| Hematologic or solid malignancy diagnosis within 5 years prior to Screening.   - Note:  Subjects with a history of localized skin cancer, basal cell or squamous cell carcinoma, may be enrolled in the study as long as they are cancer free prior to randomization.  Subjects with other localized cancers (without metastatic spread) who have previously completed their course of treatment more than 5 years prior to Screening, are not currently receiving treatment and have been in remission may be enrolled only if, in the opinion of the Investigator, there is no expectation for recurrence or further cancer treatment during the study period. Antihormonal therapy (e.g., tamoxifen) is allowed if the subject's cancer is in remission and the subject is on stable maintenance therapy to reduce their risk of recurrence.] |
| Any major surgery within 4 weeks of Screening. |
| Blood transfusion within 4 weeks of Screening. |
| History of brain surgery for Parkinsonism (i.e., deep-brain stimulation). |
| History of stem-cell treatment. |
| Women who are pregnant or breastfeeding. |
| Subjects or prisoners who are involuntarily detained or incarcerated for treatment of either a psychiatric or physical illness must not be enrolled into the study. |
| Any medical condition, based on the judgement of the Investigator, that would confound the ability to adequately assess safety and efficacy outcome measures |
| Evidence of organ dysfunction or any clinically significant deviation from normal in physical examination, vital signs, ECG, or clinical laboratory determinations beyond what is consistent with the target population. |
| Clinically significant abnormality on 12-lead ECG prior to study drug administration beyond what is consistent with the target population, confirmed by repeat. |
| QTcF (Fridericia) interval ≥ 470 msec during the Screening/Baseline period or uncontrolled arrhythmia or frequent premature ventricular contraction (PVCs) (> 5/minute) or Mobitz Type II second or third degree atrioventricular (AV) block or left bundle branch block, or right bundle branch block with a QRS duration ≥ 150 msec or intraventricular conduction defect with a QRS duration ≥ 150 msec or evidence of acute or sub-acute myocardial infarction or ischemia or other ECG findings that, in the Investigator’s opinion, would preclude participation in the study. |
| Abnormal free T4 levels and TSH > 10 mIU/L (despite treatment) at Screening, confirmed by repeat. |
| Total bilirubin, alanine aminotransferase (ALT) or aspartate aminotransferase (AST) greater than 2 times the upper limit of normal (ULN), confirmed by repeat. If the patient is diagnosed as having Gilbert’s syndrome, the patient can be discussed with the Medical Monitor. |
| Pathologic renal findings at Screening as defined by the presence of either of the following criteria:   - Estimated glomerular filtration rate (eGFR) according to the re- expressed abbreviated (four-variable) Modification of Diet in Renal Disease (MDRD) Study equation < 30 ml/min/ 1.73m^2; The MDRD estimation is calculated as follows: - eGFR (mL/min/1.73m^2) = 175 x (standardized Scr)-1.154 x (Age)-0.203 x (0.742 if female) x (1.212 if Black). [Scr: Standardized serum creatinine]; - Creatinine ≥ 2mg/dL. |
| Hematologic abnormalities at Screening:  Hemoglobin < 10 g/dL; orWBC < 3.0 x 103/mm^3; orPlatelet count < 100,000/mm^3; orNeutrophils, Absolute ≤ 1000/mm^3;  Hemoglobin A1C > 7.5%, confirmed by repeat. |
| Urine drug screen positive for a drug of abuse, for which the patient does not have a valid prescription and is suspected of abusing, in the judgement of the Investigator.   - Note: urine drug screen positive for cannabis is exclusionary unless the Investigator and Medical Monitor agree that the subject can abstain from use for the duration of the study, or if the subject has a valid medical prescription. |
| Human Immunodeficiency Virus (HIV) positive at Screening (indicated by positive confirmatory Western Blot). |
| HBsAg or HCV positive at Screening. |
| For WOCBP, positive serum β-hCG which is indicative of pregnancy and not false positive at Screening or a positive urine pregnancy test at Baseline. |

**Supplementary Table 21: Sample sizes required for a 2-arm, 6-month follow-up therapeutic trial to detect 20%, 25%, 30%, 40% and 50% change in Progressive Supranuclear Palsy and trial-eligible Progressive Supranuclear Palsy**

| **6 Month** |  | **20% change** | | **25% change** | | **30% change** | | **40% change** | | **50% change** | |  |
| --- | --- | --- | --- | --- | --- | --- | --- | --- | --- | --- | --- | --- |
|  | **Difference mean (SD)** | **Effect size** | **Sample size** | **Effect size** | **Sample size** | **Effect size** | **Sample size** | **Effect size** | **Sample size** | **Effect size** | **Sample size** |  |
| **PSP (Trial)** |  |  |  |  |  |  |  |  |  |  |  |  |
| CBD-FS | 11.6 (17.3) | 0.134 | 875 | 0.168 | 557 | 0.201 | 390 | 0.268 | 220 | 0.335 | 141 |  |
| SEADL | -13.0 (28.7) | -0.091 | 1897 | -0.113 | 1230 | -0.136 | 850 | -0.181 | 480 | -0.227 | 306 |  |
| MS | 0 (1.6) | 0.003 | 1744192 | 0.004 | 981109 | 0.004 | 981109 | 0.006 | 436049 | 0.007 | 320363 |  |
| PSP-QoL | 16.5 (32.6) | 0.102 | 1510 | 0.127 | 974 | 0.152 | 680 | 0.203 | 382 | 0.254 | 244 |  |
|  |  |  |  |  |  |  |  |  |  |  |  |  |
| UPDRS II | 8.2 (9.3) | 0.178 | 496 | 0.223 | 317 | 0.267 | 221 | 0.356 | 125 | 0.445 | 80 |  |
| UPDRS III | 8.3 (27.7) | 0.06 | 4361 | 0.075 | 2792 | 0.089 | 1983 | 0.119 | 1109 | 0.149 | 708 |  |
| PSPRS | 11.3 (14.2) | 0.159 | 622 | 0.199 | 397 | 0.239 | 276 | 0.319 | 155 | 0.399 | 100 |  |
| mPSPRS | 3.0 (5.6) | 0.107 | 1372 | 0.134 | 875 | 0.161 | 607 | 0.215 | 341 | 0.268 | 220 |  |
|  |  |  |  |  |  |  |  |  |  |  |  |  |
| UPDRS I | -1.2 (6.2) | -0.04 | 9812 | -0.05 | 6280 | -0.06 | 4361 | -0.08 | 2454 | -0.1 | 1571 |  |
| CBI-R | 12.1 (34.0) | 0.071 | 3115 | 0.089 | 1983 | 0.107 | 1372 | 0.143 | 769 | 0.179 | 491 |  |
| MoCA | -0.1 (4.8) | -0.004 | 981109 | -0.005 | 627910 | -0.006 | 436049 | -0.008 | 245278 | -0.009 | 193800 |  |
| ACE III | 2.6 (7.8) | 0.066 | 3605 | 0.082 | 2336 | 0.099 | 1603 | 0.132 | 902 | 0.165 | 578 |  |
| ECAS | 0.3 (17.0) | 0.004 | 981109 | 0.004 | 981109 | 0.005 | 627910 | 0.007 | 320363 | 0.009 | 193800 |  |
| FTD-FRS | 7.2 (14.0) | 0.103 | 1481 | 0.129 | 944 | 0.155 | 654 | 0.207 | 367 | 0.259 | 235 |  |
|  |  |  |  |  |  |  |  |  |  |  |  |  |
| **PSP (All)** |  |  |  |  |  |  |  |  |  |  |  |  |
| CBD-FS | 11.8 (21.0) | 0.113 | 1230 | 0.141 | 791 | 0.169 | 551 | 0.225 | 311 | 0.281 | 200 |  |
| SEADL | -5.8 (37.6) | -0.031 | 16336 | -0.039 | 10322 | -0.046 | 7420 | -0.062 | 4085 | -0.077 | 2649 |  |
| MS | 0.3 (2.4) | 0.024 | 27254 | 0.03 | 17443 | 0.036 | 12113 | 0.048 | 6814 | 0.06 | 4361 |  |
| PSP-QoL | 22.0 (34.4) | 0.128 | 959 | 0.16 | 614 | 0.192 | 427 | 0.256 | 240 | 0.32 | 154 |  |
|  |  |  |  |  |  |  |  |  |  |  |  |  |
| UPDRS II | 7.3 (10.8) | 0.135 | 862 | 0.168 | 557 | 0.202 | 386 | 0.269 | 218 | 0.337 | 139 |  |
| UPDRS III | 7.3 (25.4) | 0.058 | 4667 | 0.072 | 3029 | 0.087 | 2075 | 0.115 | 1188 | 0.144 | 758 |  |
| PSPRS | 6.9 (16.6) | 0.083 | 2280 | 0.103 | 1481 | 0.124 | 1022 | 0.165 | 578 | 0.207 | 367 |  |
| mPSPRS | 1.8 (5.6) | 0.064 | 3833 | 0.081 | 2394 | 0.097 | 1669 | 0.129 | 944 | 0.161 | 607 |  |
|  |  |  |  |  |  |  |  |  |  |  |  |  |
| UPDRS I | -0.8 (6.9) | -0.024 | 27254 | -0.03 | 17443 | -0.036 | 12113 | -0.048 | 6814 | -0.06 | 4361 |  |
| CBI-R | 13.7 (32.1) | 0.085 | 2174 | 0.107 | 1372 | 0.128 | 959 | 0.171 | 538 | 0.213 | 347 |  |
| MoCA | -0.7 (5.8) | -0.024 | 27254 | -0.03 | 17443 | -0.036 | 12113 | -0.048 | 6814 | -0.06 | 4361 |  |
| ACE III | -0.9 (9.8) | -0.018 | 48451 | -0.023 | 29675 | -0.027 | 21534 | -0.036 | 12113 | -0.045 | 7753 |  |
| ECAS | -7.8 (21.7) | -0.072 | 3029 | -0.09 | 1939 | -0.108 | 1347 | -0.144 | 758 | -0.18 | 485 |  |
| FTD-FRS | 7.1 (14.2) | 0.099 | 1603 | 0.124 | 1022 | 0.149 | 708 | 0.199 | 397 | 0.248 | 256 |  |
|  | | | | | | | | | | | |  |
| Variables with fewer than 5 responses per group have been removed. Abbreviations: ACE III = Addenbrookes Cognitive Examination III, CBD-FS = Corticobasal Degeneration Functional Scale, CBI-R = Cambridge Behavioural Inventory Revised, CBS = corticobasal syndrome , ECAS = Edinburgh Cognitive and Behavioural Screen, FTD-FRS = Frontotemporal Dementia Rating Scale, IDT = Indeterminate, MoCA = Montreal Cognitive Assessment, mPSPRS = modified Progressive Supranuclear Palsy Rating Scale, PSP = progressive supranuclear palsy, PSP QoL = PSP Quality of life scale, PSPRS = PSP rating scale, SD = standard deviation, SEADL = Schwab and England Activities of Daily Living Scale, UPDRS = Unified Parkinson’s Disease Rating Scale | | | | | | | | | | | | |

**Supplementary Table 22: Sample sizes required for a 2-arm, 6-month follow-up therapeutic trial to detect 20%, 25%, 30%, 40% and 50% change in Multiple System Atrophy and trial-eligible Multiple System Atrophy**

| **6 Month** |  | **20% change** | | **25% change** | | **30% change** | | **40% change** | | **50% change** | |
| --- | --- | --- | --- | --- | --- | --- | --- | --- | --- | --- | --- |
|  | **Difference mean (SD)** | **Effect size** | **Sample size** | **Effect size** | **Sample size** | **Effect size** | **Sample size** | **Effect size** | **Sample size** | **Effect size** | **Sample size** |
| **MSA (Trial)** |  |  |  |  |  |  |  |  |  |  |  |
| SEADL | -15.0 (18.4) | -0.163 | 592 | -0.204 | 378 | -0.244 | 265 | -0.326 | 149 | -0.407 | 96 |
| MS | 1.0 (1.8) | 0.112 | 1252 | 0.141 | 791 | 0.169 | 551 | 0.225 | 311 | 0.281 | 200 |
| MSA-QoL | 12.1 (21.7) | 0.111 | 1275 | 0.139 | 813 | 0.167 | 564 | 0.223 | 317 | 0.279 | 203 |
|  |  |  |  |  |  |  |  |  |  |  |  |
| UPDRS II | 1.0 (9.7) | 0.021 | 35597 | 0.026 | 23222 | 0.032 | 15331 | 0.042 | 8900 | 0.053 | 5589 |
| UMSARS | 10.8 (17.5) | 0.123 | 1039 | 0.154 | 663 | 0.185 | 460 | 0.246 | 260 | 0.308 | 166 |
|  |  |  |  |  |  |  |  |  |  |  |  |
| UPDRS I | -2.2 (6.0) | -0.075 | 2792 | -0.093 | 1816 | -0.112 | 1252 | -0.149 | 708 | -0.187 | 450 |
| CBI-R | -0.1 (22.3) | -0.001 | 15697720 | -0.001 | 15697720 | -0.001 | 15697720 | -0.001 | 15697720 | -0.002 | 3924431 |
| MoCA | -1.9 (4.4) | -0.087 | 2075 | -0.109 | 1322 | -0.131 | 916 | -0.174 | 519 | -0.218 | 331 |
| ACE III | -1.8 (11.4) | -0.032 | 15331 | -0.04 | 9812 | -0.048 | 6814 | -0.064 | 3833 | -0.08 | 2454 |
| ECAS | -6.3 (10.9) | -0.115 | 1188 | -0.144 | 758 | -0.173 | 525 | -0.231 | 295 | -0.288 | 190 |
| FTD-FRS | 0.8 (9.1) | 0.017 | 54318 | 0.021 | 35597 | 0.025 | 25117 | 0.033 | 14416 | 0.042 | 8900 |
|  |  |  |  |  |  |  |  |  |  |  |  |
| **MSA (All)** |  |  |  |  |  |  |  |  |  |  |  |
| SEADL | -13.2 (24.0) | -0.11 | 1298 | -0.138 | 825 | -0.165 | 578 | -0.22 | 325 | -0.275 | 209 |
| MS | 1.1 (1.8) | 0.122 | 1056 | 0.152 | 680 | 0.183 | 470 | 0.244 | 265 | 0.305 | 170 |
| MSA-QoL | 5.7 (24.4) | 0.046 | 7420 | 0.058 | 4667 | 0.07 | 3205 | 0.093 | 1816 | 0.116 | 1168 |
|  |  |  |  |  |  |  |  |  |  |  |  |
| UPDRS II | 2.2 (11.0) | 0.039 | 10322 | 0.049 | 6539 | 0.059 | 4511 | 0.079 | 2516 | 0.098 | 1635 |
| UMSARS | 10.3 (15.1) | 0.136 | 850 | 0.17 | 544 | 0.204 | 378 | 0.272 | 213 | 0.34 | 137 |
|  |  |  |  |  |  |  |  |  |  |  |  |
| UPDRS I | -1.8 (5.4) | -0.069 | 3298 | -0.086 | 2123 | -0.103 | 1481 | -0.137 | 837 | -0.172 | 532 |
| CBI-R | -0.3 (25.4) | -0.003 | 1744192 | -0.003 | 1744192 | -0.004 | 981109 | -0.005 | 627910 | -0.007 | 320363 |
| MoCA | -1.4 (5.6) | -0.051 | 6036 | -0.064 | 3833 | -0.077 | 2649 | -0.102 | 1510 | -0.128 | 959 |
| ACE III | 0.7 (11.5) | 0.013 | 92887 | 0.016 | 61320 | 0.019 | 43485 | 0.026 | 23222 | 0.032 | 15331 |
| ECAS | -6.9 (17.1) | -0.081 | 2394 | -0.101 | 1540 | -0.121 | 1073 | -0.162 | 599 | -0.202 | 386 |
| FTD-FRS | -3.6 (10.6) | -0.068 | 3396 | -0.085 | 2174 | -0.102 | 1510 | -0.136 | 850 | -0.17 | 544 |
|  | | | | | | | | | | | |
| Variables with fewer than 5 responses per group have been removed. Abbreviations: ACE III = Addenbrookes Cognitive Examination III, CBI-R = Cambridge Behavioural Inventory Revised, ECAS = Edinburgh Cognitive and Behavioural Screen, FTD-FRS = Frontotemporal Dementia Rating Scale, MoCA = Montreal Cognitive Assessment, MSA = multiple system atrophy, MSA-C = MSA cerebellar variant, MSA-P = MSA parkinsonism variant, MSA QoL = MSA Quality of life scale, SD = standard deviation, SEADL = Schwab and England Activities of Daily Living Scale, UPDRS = Unified Parkinson’s Disease Rating Scale, UMSARS = Unified MSA Rating Scale | | | | | | | | | | | |

**Supplementary Table 23: Sample sizes required for a 2-arm, 12-month follow-up therapeutic trial to detect 20%, 25%, 30%, 40% and 50% change in trial-eligible Progressive Supranuclear Palsy and Multiple System Atrophy**

| **12 Month** |  | **20% change** | | **25% change** | | **30% change** | | **40% change** | | **50% change** | |  |
| --- | --- | --- | --- | --- | --- | --- | --- | --- | --- | --- | --- | --- |
|  | **Difference mean (SD)** | **Effect size** | **Sample size** | **Effect size** | **Sample size** | **Effect size** | **Sample size** | **Effect size** | **Sample size** | **Effect size** | **Sample size** |  |
| **PSP (Trial)** |  |  |  |  |  |  |  |  |  |  |  |  |
| CBD-FS | 7.8 (7.8) | 0.200 | 393 | 0.250 | 252 | 0.3 | 175 | 0.400 | 99 | 0.500 | 64 |  |
| SEADL | -16.9 (18.3) | -0.185 | 460 | -0.231 | 295 | -0.278 | 204 | -0.370 | 116 | -0.463 | 74 |  |
| MS | 0.9 (1.3) | 0.137 | 837 | 0.171 | 538 | 0.205 | 374 | 0.273 | 212 | 0.342 | 135 |  |
| PSP-QoL | 16.1 (19.8) | 0.162 | 599 | 0.203 | 382 | 0.243 | 267 | 0.325 | 150 | 0.406 | 96 |  |
|  |  |  |  |  |  |  |  |  |  |  |  |  |
| UPDRS II | 7.2 (5.8) | 0.248 | 256 | 0.310 | 164 | 0.372 | 114 | 0.496 | 65 | 0.620 | 42 |  |
| UPDRS III | 9.6 (13.6) | 0.142 | 779 | 0.177 | 502 | 0.213 | 347 | 0.283 | 197 | 0.354 | 126 |  |
| PSPRS | 12.2 (8.5) | 0.286 | 193 | 0.357 | 124 | 0.429 | 86 | 0.572 | 49 | 0.715 | 32 |  |
| mPSPRS | 4.1 (3.1) | 0.264 | 226 | 0.330 | 145 | 0.396 | 101 | 0.529 | 57 | 0.661 | 37 |  |
|  |  |  |  |  |  |  |  |  |  |  |  |  |
| UPDRS I | 0.3 (3.5) | 0.017 | 54318 | 0.021 | 35597 | 0.025 | 25117 | 0.033 | 14416 | 0.042 | 8900 |  |
| CBI-R | 14.0 (21.6) | 0.13 | 930 | 0.162 | 599 | 0.194 | 418 | 0.259 | 235 | 0.324 | 151 |  |
| MoCA | -0.5 (2.8) | -0.036 | 12113 | -0.045 | 7753 | -0.054 | 5384 | -0.072 | 3029 | -0.09 | 1939 |  |
| ACE III | -0.9 (4.8) | -0.038 | 10872 | -0.048 | 6814 | -0.058 | 4667 | -0.077 | 2649 | -0.096 | 1704 |  |
| ECAS | -1.2 (10.8) | -0.021 | 35597 | -0.027 | 21534 | -0.032 | 15331 | -0.043 | 8491 | -0.053 | 5589 |  |
| FTD-FRS | 4.3 (8.8) | 0.098 | 1635 | 0.122 | 1056 | 0.146 | 737 | 0.195 | 414 | 0.244 | 265 |  |
|  |  |  |  |  |  |  |  |  |  |  |  |  |
| **MSA (Trial)** |  |  |  |  |  |  |  |  |  |  |  |  |
| SEADL | -12.3 (13.8) | -0.178 | 496 | -0.223 | 317 | -0.267 | 221 | -0.356 | 125 | -0.446 | 80 |  |
| MS | 0.7 (1.0) | 0.157 | 638 | 0.197 | 405 | 0.236 | 283 | 0.315 | 159 | 0.393 | 103 |  |
| MSA-QoL | 3.4 (13.8) | 0.049 | 6539 | 0.061 | 4220 | 0.073 | 2947 | 0.097 | 1669 | 0.122 | 1056 |  |
|  |  |  |  |  |  |  |  |  |  |  |  |  |
| UPDRS II | 3.0 (6.0) | 0.101 | 1540 | 0.127 | 974 | 0.152 | 680 | 0.203 | 382 | 0.254 | 244 |  |
| UMSARS | 10.5 (10.0) | 0.209 | 360 | 0.261 | 231 | 0.313 | 161 | 0.418 | 91 | 0.522 | 59 |  |
|  |  |  |  |  |  |  |  |  |  |  |  |  |
| CBI-R | 11.1 (20.8) | 0.107 | 1372 | 0.133 | 888 | 0.16 | 614 | 0.213 | 347 | 0.267 | 221 |  |
| MoCA | -0.2 (2.7) | -0.013 | 92887 | -0.017 | 54318 | -0.02 | 39245 | -0.027 | 21534 | -0.033 | 14416 |  |
| ACE III | -0.5 (6.7) | -0.016 | 61320 | -0.02 | 39245 | -0.024 | 27254 | -0.032 | 15331 | -0.04 | 9812 |  |
| ECAS | -0.5 (8.1) | -0.011 | 129734 | -0.014 | 80091 | -0.017 | 54318 | -0.022 | 32434 | -0.028 | 20024 |  |
| FTD-FRS | -0.1 (5.4) | -0.004 | 981109 | -0.005 | 627910 | -0.006 | 436049 | -0.008 | 245278 | -0.01 | 156978 |  |
|  |  |  |  |  |  |  |  |  |  |  |  |  |
| Variables with fewer than 5 responses per group have been removed. Abbreviations: ACE III = Addenbrookes Cognitive Examination III, CBD-FS = Corticobasal Degeneration Functional Scale, CBI-R = Cambridge Behavioural Inventory Revised, CBS = corticobasal syndrome , ECAS = Edinburgh Cognitive and Behavioural Screen, FTD-FRS = Frontotemporal Dementia Rating Scale, IDT = Indeterminate, MoCA = Montreal Cognitive Assessment, mPSPRS = modified Progressive Supranuclear Palsy Rating Scale, PSP = progressive supranuclear palsy, PSP QoL = PSP Quality of life scale, PSPRS = PSP rating scale, SD = standard deviation, SEADL = Schwab and England Activities of Daily Living Scale, UPDRS = Unified Parkinson’s Disease Rating Scale | | | | | | | | | | | | |

**Supplementary Table 24: Sample sizes required for a 2-arm, 12-month follow-up therapeutic trial to detect 20%, 25%, 30%, 40% and 50% change in imaging markers of trial-eligible Progressive Supranuclear Palsy and Multiple System Atrophy**

| **12 Month** |  | **20% change** | | **25% change** | | **30% change** | | **40% change** | | **50% change** | |  |
| --- | --- | --- | --- | --- | --- | --- | --- | --- | --- | --- | --- | --- |
|  | **Percent difference mean (SD)** | **Effect size** | **Sample size** | **Effect size** | **Sample size** | **Effect size** | **Sample size** | **Effect size** | **Sample size** | **Effect size** | **Sample size** |  |
| **PSP (Trial)** |  |  |  |  |  |  |  |  |  |  |  |  |
| Temporal | -2.9 (2.1) | -0.226 | 308 | -0.283 | 197 | -0.339 | 138 | -0.452 | 78 | -0.565 | 50 |  |
| Cingulate | -2.9 (1.8) | -0.378 | 111 | -0.473 | 71 | -0.567 | 50 | -0.756 | 28 | -0.945 | 19 |  |
| Insula | -1.2 (2.1) | -0.129 | 944 | -0.162 | 599 | -0.194 | 418 | -0.259 | 235 | -0.323 | 151 |  |
| Occipital | -2.6 (3.1) | -0.14 | 802 | -0.174 | 519 | -0.209 | 360 | -0.279 | 203 | -0.349 | 130 |  |
| Frontal | -4.4 (3.3) | -0.354 | 126 | -0.443 | 81 | -0.532 | 56 | -0.709 | 32 | -0.886 | 21 |  |
| Parietal | -3.4 (2.6) | -0.325 | 150 | -0.407 | 96 | -0.488 | 67 | -0.651 | 38 | -0.813 | 25 |  |
| Central | -2.9 (1.9) | -0.436 | 84 | -0.545 | 54 | -0.654 | 38 | -0.872 | 22 | -1.09 | 14 |  |
| Ventricles | 11.7 (5.6) | 0.444 | 81 | 0.555 | 52 | 0.666 | 36 | 0.889 | 21 | 1.111 | 14 |  |
| Cerebellum | -3.6 (4.3) | -0.193 | 422 | -0.242 | 269 | -0.29 | 188 | -0.387 | 106 | -0.483 | 68 |  |
| Pons | -3.8 (3.2) | -0.425 | 88 | -0.531 | 57 | -0.637 | 40 | -0.849 | 23 | -1.062 | 15 |  |
| Midbrain | -3.0 (2.4) | -0.447 | 80 | -0.558 | 51 | -0.67 | 36 | -0.893 | 21 | -1.116 | 14 |  |
|  |  |  |  |  |  |  |  |  |  |  |  |  |
| **MSA (Trial)** |  |  |  |  |  |  |  |  |  |  |  |  |
| Temporal | -2.5 (2.7) | -0.175 | 514 | -0.219 | 328 | -0.263 | 228 | -0.35 | 129 | -0.438 | 83 |  |
| Cingulate | -1.6 (1.1) | -0.264 | 226 | -0.33 | 145 | -0.396 | 101 | -0.528 | 57 | -0.66 | 37 |  |
| Insula | -0.7 (1.6) | -0.081 | 2394 | -0.101 | 1540 | -0.121 | 1073 | -0.161 | 607 | -0.201 | 390 |  |
| Occipital | -2.2 (3.4) | -0.123 | 1039 | -0.154 | 663 | -0.185 | 460 | -0.247 | 258 | -0.308 | 166 |  |
| Frontal | -2.7 (1.6) | -0.317 | 157 | -0.397 | 101 | -0.476 | 70 | -0.634 | 40 | -0.793 | 26 |  |
| Parietal | -2.6 (2.6) | -0.239 | 276 | -0.298 | 178 | -0.358 | 123 | -0.477 | 70 | -0.597 | 45 |  |
| Central | -2.0 (1.2) | -0.335 | 141 | -0.418 | 91 | -0.502 | 63 | -0.669 | 36 | -0.837 | 23 |  |
| Ventricles | 7.9 (3.9) | 0.351 | 128 | 0.438 | 83 | 0.526 | 58 | 0.701 | 33 | 0.877 | 21 |  |
| Cerebellum | -4.6 (2.7) | -0.342 | 135 | -0.427 | 87 | -0.513 | 61 | -0.684 | 35 | -0.855 | 22 |  |
| Medulla | 0.001 (3.6) | -0.004 | 981109 | -0.005 | 627910 | -0.005 | 627910 | -0.007 | 320363 | -0.009 | 193800 |  |
| Pons | -4.8 (4.5) | -0.213 | 347 | -0.267 | 221 | -0.32 | 154 | -0.427 | 87 | -0.534 | 56 |  |
| Midbrain | -1.2 (2.0) | -0.128 | 959 | -0.16 | 614 | -0.192 | 427 | -0.256 | 240 | -0.32 | 154 |  |
| Abbreviations: CBS = corticobasal syndrome, IDT = Indeterminate, MSA = multiple system atrophy, PSP = progressive supranuclear palsy, SD = standard deviation. | | | | | | | | | | | | |

**Supplementary Methods**

**Fluid biomarkers**

Non-fasting serum and CSF samples were processed using standardised protocols and stored in 0.5ml aliquots at -80°C within 60 minutes of sample collection. Serum NfL was measured using an ultrasensitive single molecule (Simoa) assay.^1^Where available, CSF samples were tested for total tau and amyloid beta 1-42 (Aβ1-42) levels (INNOTEST ELISA – Fujirebio Europe N.V., Gent, Belgium.^2^

Cases with corticobasal syndrome were stratified according to CSF findings using the below rationale as used in previous studies^3,4^:

| **Phenotype** | **Finding** |
| --- | --- |
| CBS-4RT | CSF T-tau:Aβ1-42 ratio <1 |
| CBS-AD | CSF T-tau:Aβ1-42 ratio >1 |
| CBS-IDT | CSF not available |

**Neuroimaging**

Scan protocols were closely matched across centres and based on the international Genetic Frontotemporal Dementia Initiative protocols (MP-RAGE, TR 2s, TE 2.93ms, Flip angle 8deg, 1.1mm isotropic).^5^ T1-weighted images were processed using the recon-all pipeline of FreeSurfer 6.0.0 (Massachusetts General Hospital, Harvard Medical School; <http://surfer.nmr.mgh.harvard.edu/>) into subcortical segments and cortical surface parcellations with adjustments for large ventricles and additional brainstem structures parcellation. Regional analyses were performed using volume measures from 68 Desikan-Killiany atlas cortical regions and 38 subcortical volume measures from the segmentation.^6,7^ Volume measures from parcellation were combined to calculate cortical grey matter volumes of the frontal, temporal, parietal, occipital lobes. Hippocampi and amygdalae volumes are included in the temporal lobe data. The remaining segmentation regions were combined into central structures (basal ganglia, thalamus, accumbens), cerebellar grey matter, brainstem, and ventricles. The images were additionally segmented into grey, white and CSF modulated probability maps using CAT12 (neuro.uni-jena.de/cat) in order to obtain total intracranial volume measures. Volumetric output data was then imported to R Studio (version 4.0.3) for further analysis including construction of linear mixed models of annual change. We also calculated the ratio of the pons to midbrain volume.

In individuals without CSF results, cases with corticobasal syndrome were stratified according to amyloid PET findings when available. Amyloid PET imaging was performed using Pittsburgh compound B ([^11^C]PiB), and cortical standardised uptake value ratio (SUVR; 50-70 minutes post-injection; whole cerebellum reference tissue) was determined using the Centiloid Project methodology at a single centre (Cambridge).^8^ The below values determined amyloid negativity as used in previous studies and were obtained by converting the Centiloid cutoff of 19 to SUVR using the Centiloid-to-SUVR transformation^9,10^:

| **Phenotype** | **Finding** |
| --- | --- |
| CBS-4RT | [^11^C]PiB SUVR <1.21 |
| CBS-AD | [^11^C]PiB SUVR >1.21 |
| CBS-IDT | Amyloid PET not available |

**Genetics**

DNA was extracted from serum samples of PSP, CBS and IDT cases. DNA samples subsequently underwent genotyping at UCL Institute of Child Health using the Illumina NeuroChip for white cases.^11^ Standard steps taken for data quality control and single nucleotide polymorphism imputation have previously been described.^12^ White control allele/haplotype frequencies were derived from dbSNP (www.ncbi.nlm.nih.gov/snp). MAPT H1/H1 (determined by rs1800547 genotype), TRIM11 (determined by rs564309 minor allele frequency), LRRK2 (determined by rs2242367 minor allele frequency) and *APOE*-ε4 allele (determined by rs429358 and rs7412 genotypes) frequencies were calculated using imputed genetic datasets.

**References:**

1. Kuhle J, Barro C, Andreasson U, Derfuss T, Lindberg R, Sandelius Å, et al. Comparison of three analytical platforms for quantification of the neurofilament light chain in blood samples: ELISA, electrochemiluminescence immunoassay and Simoa. Clin Chem Lab Med [Internet]. 2016 Oct 1;54(10):1655–61. Available from: http://www.ncbi.nlm.nih.gov/pubmed/27071153

2. Faull M, Ching SY, Jarmolowicz AI, Beilby J, Panegyres PK. Comparison of two methods for the analysis of CSF Aβ and tau in the diagnosis of Alzheimer’s disease. Am J Neurodegener Dis [Internet]. 2014;3(3):143–51. Available from: http://www.ncbi.nlm.nih.gov/pubmed/25628965

3. Borroni B, Premi E, Agosti C, Alberici A, Cerini C, Archetti S, et al. CSF Alzheimer’s disease-like pattern in corticobasal syndrome: evidence for a distinct disorder. J Neurol Neurosurg Psychiatry [Internet]. 2011 Aug;82(8):834–8. Available from: http://www.ncbi.nlm.nih.gov/pubmed/21345847

4. Jabbari E, Holland N, Chelban V, Jones PS, Lamb R, Rawlinson C, et al. Diagnosis Across the Spectrum of Progressive Supranuclear Palsy and Corticobasal Syndrome. JAMA Neurol. 2020;77(3):377–87.

5. Rohrer JD, Nicholas JM, Cash DM, van Swieten J, Dopper E, Jiskoot L, et al. Presymptomatic cognitive and neuroanatomical changes in genetic frontotemporal dementia in the Genetic Frontotemporal dementia Initiative (GENFI) study: A cross-sectional analysis. Lancet Neurol. 2015;14(3):253–62.

6. Fischl B, Salat DH, Busa E, Albert M, Dieterich M, Haselgrove C, et al. Whole brain segmentation: Automated labeling of neuroanatomical structures in the human brain. Neuron. 2002;33(3):341–55.

7. Iglesias JE, Van Leemput K, Bhatt P, Casillas C, Dutt S, Schuff N, et al. Bayesian segmentation of brainstem structures in MRI. Neuroimage. 2015;113:184–95.

8. Klunk WE, Koeppe RA, Price JC, Benzinger TL, Devous MD, Jagust WJ, et al. The Centiloid Project: standardizing quantitative amyloid plaque estimation by PET. Alzheimers Dement [Internet]. 2015 Jan;11(1):1-15.e1-4. Available from: http://www.ncbi.nlm.nih.gov/pubmed/25443857

9. Jack CR, Wiste HJ, Weigand SD, Therneau TM, Lowe VJ, Knopman DS, et al. Defining imaging biomarker cut points for brain aging and Alzheimer’s disease. Alzheimers Dement [Internet]. 2017 Mar;13(3):205–16. Available from: http://www.ncbi.nlm.nih.gov/pubmed/27697430

10. Holland N, Malpetti M, Rittman T, Mak EE, Passamonti L, Kaalund SS, et al. Molecular pathology and synaptic loss in primary tauopathies: an 18F-AV-1451 and 11C-UCB-J PET study. Brain [Internet]. 2022 Mar 29;145(1):340–8. Available from: http://www.ncbi.nlm.nih.gov/pubmed/34398211

11. Blauwendraat C, Faghri F, Pihlstrom L, Geiger JT, Elbaz A, Lesage S, et al. NeuroChip, an updated version of the NeuroX genotyping platform to rapidly screen for variants associated with neurological diseases. Neurobiol Aging [Internet]. 2017;57:247.e9-247.e13. Available from: http://www.ncbi.nlm.nih.gov/pubmed/28602509

12. Reed E, Nunez S, Kulp D, Qian J, Reilly MP, Foulkes AS. A guide to genome-wide association analysis and post-analytic interrogation. Stat Med [Internet]. 2015 Dec 10;34(28):3769–92. Available from: http://www.ncbi.nlm.nih.gov/pubmed/26343929
