# Supplementary material for: Progression of atypical parkinsonian syndromes: PROSPECT-M-UK study implications for clinical trials
Source: Brain. 2023 Mar 28;146(8):3232–42. doi: 10.1093/brain/awad105 (PMC10393398; doi:10.1093/brain/awad105)
Supplement: Supplementary file 1 [file awad105_supplementary_data.pdf]

Supplementary Material Contents

**Supplementary Figure 1**	**Page 3**
**Supplementary Figure 2**	**Page 4**
**Supplementary Figure 3**	**Page 5**
**Supplementary Table 1**	**Page 6**
**Supplementary Table 2**	**Page 7**
**Supplementary Table 3**	**Page 8**
**Supplementary Table 4**	**Page 9**
**Supplementary Table 5**	**Page 10**
**Supplementary Table 6**	**Page 11**
**Supplementary Table 7**	**Page 12**
**Supplementary Table 8**	**Page 13**
**Supplementary Table 9**	**Page 14**
**Supplementary Table 10**	**Page 15**
**Supplementary Table 11**	**Page 16**
**Supplementary Table 12**	**Page 17**
**Supplementary Table 13**	**Page 18**
**Supplementary Table 14**	**Page 19**
**Supplementary Table 15**	**Page 20**
**Supplementary Table 16**	**Page 21**
**Supplementary Table 17**	**Page 22**
**Supplementary Table 18**	**Page 23**
**Supplementary Table 19**	**Page 24-25**
**Supplementary Table 20**	**Page 26-28**

**Supplementary Table 21**	**Page 29**
**Supplementary Table 22**	**Page 30**
**Supplementary Table 23**	**Page 31**
**Supplementary Table 24**	**Page 32**
**Supplementary Methods**	**Page 33**
**Supplementary References**	**Page 34**

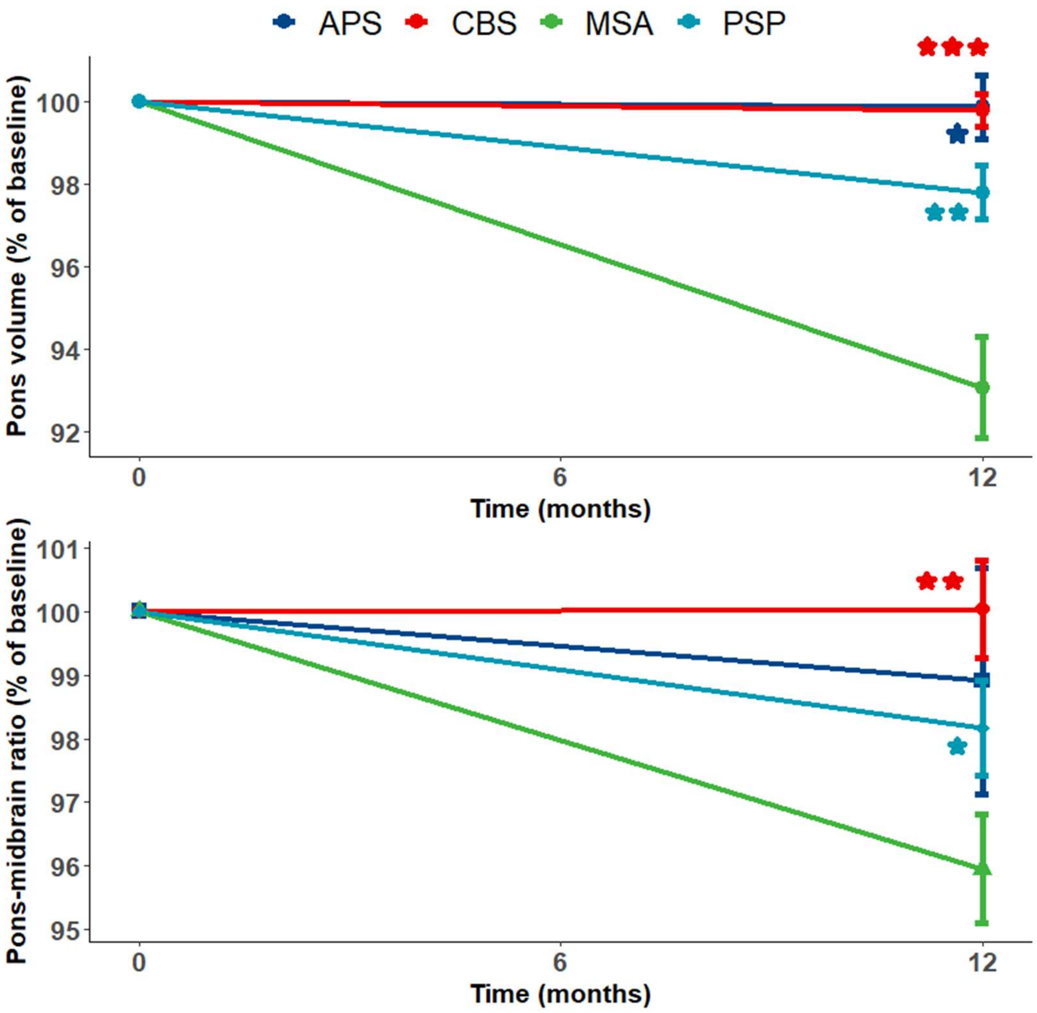

<u>Supplementary Figure 1</u>: Longitudinal changes in brain volume. Mean values at baseline and 12 months for indeterminate (APS), corticobasal syndrome (CBS), multiple system atrophy (MSA) and progressive supranuclear palsy (PSP) groups. Significance change difference is presented relative to MSA over 0-12 months by *p<0.05, **p<0.01 and ***p<0.001.

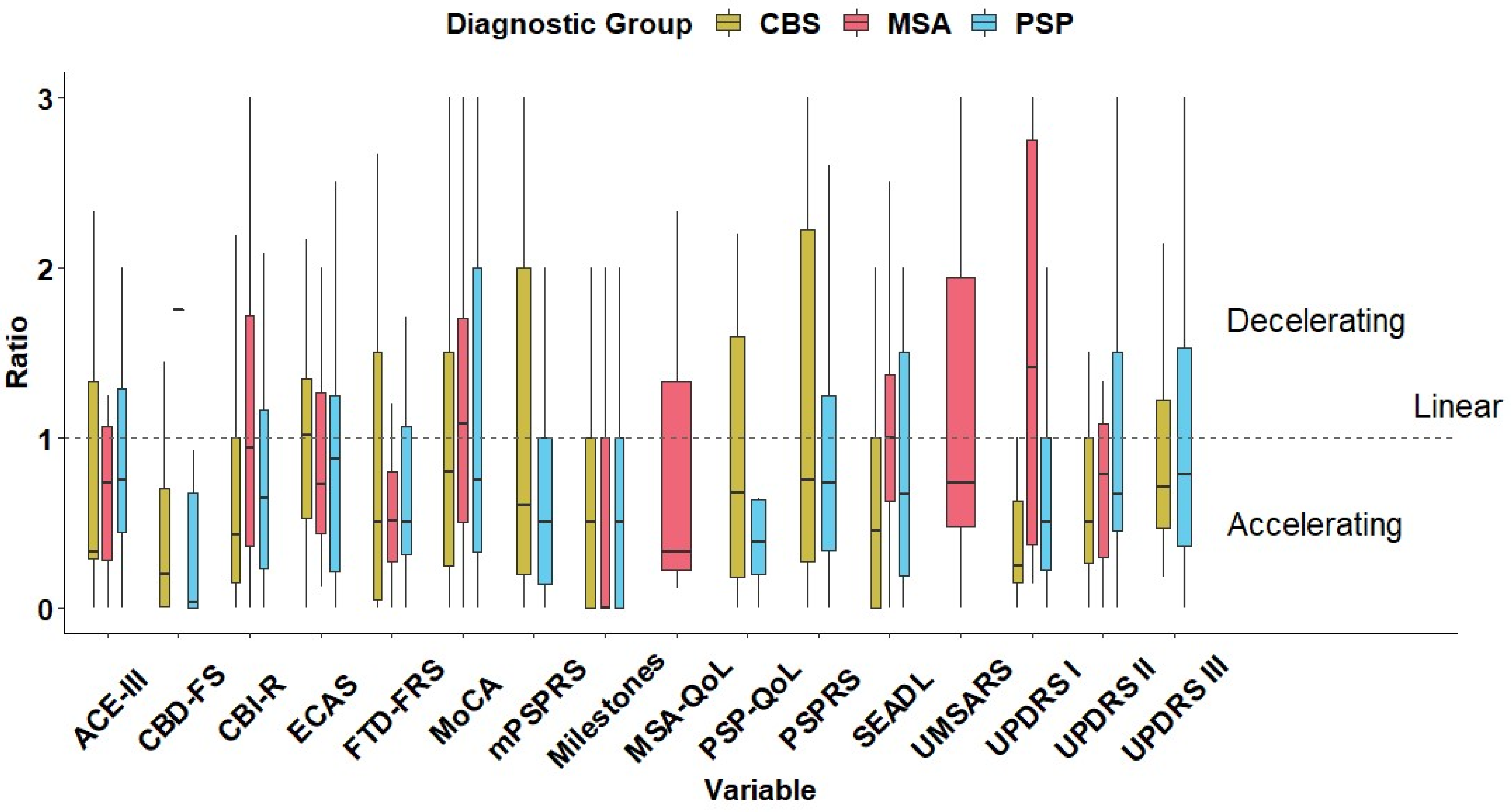

<u>Supplementary Figure 2:</u> Ratios of rate of biomarker change between 0-6 months and 6-12 months split according to diagnostic group. Abbreviations: ACE III = Addenbrookes Cognitive Examination III, CBD-FS = Corticobasal Degeneration Functional Scale, CBI-R = Cambridge Behavioural Inventory Revised, CBS = corticobasal syndrome , ECAS = Edinburgh Cognitive and Behavioural Screen, FTD-FRS = Frontotemporal Dementia Rating Scale, MoCA = Montreal Cognitive Assessment, mPSPRS = modified Progressive Supranuclear Palsy Rating Scale, MSA = multiple system atrophy, MSA-QoL = MSA Quality of life scale, PSP = progressive supranuclear palsy, PSP-QoL = PSP Quality of life scale, PSPRS = PSP rating scale, SEADL = Schwab and England Activities of Daily Living Scale, UPDRS = Unified Parkinson's Disease Rating Scale, UMSARS = Unified MSA Rating Scale.

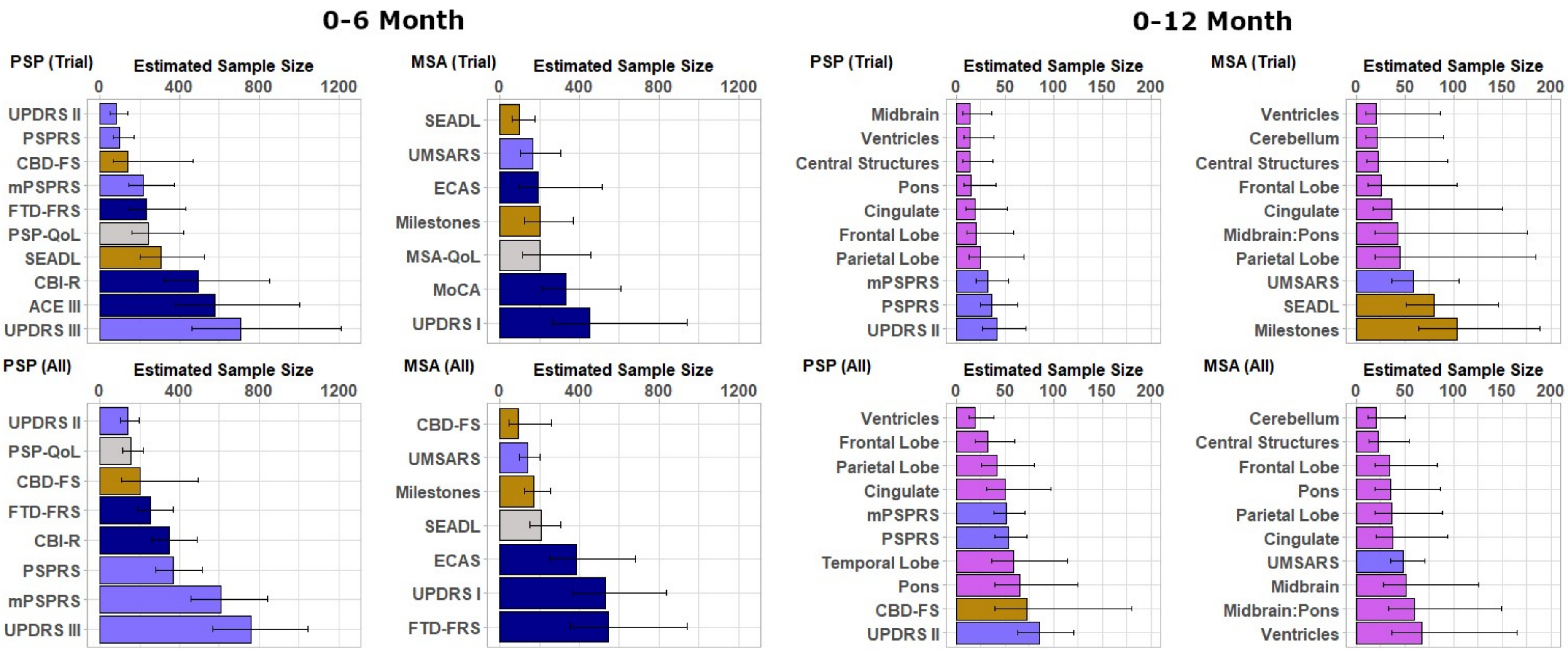

<u>Supplementary Figure 3:</u> Estimated sample size and bootstrapped confidence interval plots by six- (left); and twelve-month (right) progression in trial eligible groups (N.B. Excluded results with estimated sample sizes >1000). Abbreviations: ACE III = Addenbrookes Cognitive Examination III, CBD-FS = Corticobasal Degeneration Functional Scale, CBI-R = Cambridge Behavioural Inventory Revised, ECAS = Edinburgh Cognitive and Behavioural Screen, FTD-FRS = Frontotemporal Dementia Rating Scale, MoCA = Montreal Cognitive Assessment, mPSPRS = modified Progressive Supranuclear Palsy Rating Scale, MSA = multiple system atrophy, MSA-QoL = MSA Quality of life scale, PSP = progressive supranuclear palsy, PSP-QoL = PSP Quality of life scale, PSPRS = PSP rating scale, SEADL = Schwab and England Activities of Daily Living Scale, UPDRS = Unified Parkinson's Disease Rating Scale, UMSARS = Unified MSA Rating Scale.

Supplementary Table 1: Baseline values of Activity of Daily Living, Quality of Life, Motor Examination, Neuropsychiatric and fluid markers by Final diagnosis

	PSP				CBS				MSA			IDT
	All (n=117)	RS (n=57)	Cortical (n=28)	Subcortical (n=32)	All (n=42)	4RT (n=10)	AD (n=10)	Unknown (n=22)	All (n=68)	Parkinsonism (n=41)	Cerebellar (n=26)	(n=16)
**CBD-FS**	41·3 (19·1)	33·2 (18·7)	49·8 (19·4)	36·0 (10·6)	36·7 (22·2)	45·2 (19·1)	40·0 (27·8)	21·8 (9·2)	··	··	··	32·3 (11·6)
**SEADL**	54·3 (21·9)	57·0 (22·6)	41·8 (20·6)[r]	60·6 (17·7)	53·3 (21·7)[b]	48·0 (21·5)	55·0 (21·2)	55·0 (22·6)	57·2 (24·2)[b]	56·6 (25·7)	58·1 (22·1)	73·3 (18·0)[d]
**Milestones**	3·5 (1·6)[i]	3·7 (1·5)	4·0 (1·6)	2·8 (1·6)	1·9 (2·0)	2·5 (2·8)	1·2 (1·6)	1·9 (1·7)	2·0 (1·4)	1·8 (1·5)	2·2 (1·3)	1·8 (1·3)[c,k]
**MSA QoL**	··	··	··	··	··	··	··	··	43·5 (16·6)	44·5 (18·1)	42·3 (15·0)	··
**PSP QoL**	63·0 (32·8)	58·7 (31·7)[o]	84·5 (37·9)[q]	53·6 (22·3)	52·7 (28·6)	65·3 (32·1)	42·8 (15·8)	48·5 (29·3)	··	··	··	48·8 (25·6)
**UPDRS II**	23·5 (9·5)	20·2 (8·4)[n]	29·2 (11·1)	24·1 (7·4)	20·2 (10·2)	25·8 (11·5)	16·2 (6·4)	18·3 (9·6)	23·6 (9·6)[b]	23·6 (9·4)	23·6 (10·2)	15·9 (7·3)[d]
**UPDRS III**	44·0 (17·3)	38·9 (15·1)[n]	56·0 (20·8)	42·4 (12·2)	49·3 (22·7)[a,h]	53·4 (20·4)	48·8 (21·7)	47·6 (24·7)	··	··	··	30·1 (16·0)[d]
**PSPRS**	33·0 (13·0)	31·3 (11·6)[o]	42·4 (13·7)[p]	27·6 (10·5)	30·2 (14·6)[b]	37·9 (15·4)	29·3 (16·7)	27·0 (12·4)	··	··	··	19·5 (12·1)[c,m]
**mPSPRS**	13·3 (4·2)	12·9 (4·1)	15·3 (4·3)	12·3 (3·8)	10·3 (5·4)	12·6 (4·7)	10·1 (5·6)	9·3 (5·5)	··	··	··	7·9 (4·6)
**UMSARS**	··	··	··	··	··	··	··	··	45·0 (15·2)	43·4 (15·8)	47·2 (14·2)	··
**UPDRS I**	9·6 (4·5)	9·2 (4·0)	11·5 (5·6)	8·6 (3·8)	8·3 (5·2)[f]	11·6 (5·5)[s]	3·3 (1·2)	8·2 (4·6)	13·1 (5·0)	13·9 (4·6)	12·0 (5·5)	11·5 (3·9)[l]
**CBI-R**	46·3 (30·9)	46·5 (30·1)	60·4 (37·4)[q]	34·1 (20·3)	40·5 (27·4)	53·0 (29·7)	54·7 (32·2)	27·4 (17·5)	34·7 (20·7)	32·9 (18·6)	37·3 (23·6)	24·8 (11·3)
**MoCA**	22·2 (4·8)[j]	22·3 (4·0)	20·5 (5·7)	23·5 (5·1)	19·7 (7·9)[e]	21·5 (8·4)	14·2 (8·5)[t]	21·3 (6·3)	25·3 (3·2)	25·4 (3·4)	25·0 (3·1)	22·5 (5·1)[m]
**ACE III**	78·8 (13·3)[j]	79·2 (10·7)	71·0 (18·1)[r]	84·2 (10·9)	71·4 (22·8)[e]	80·9 (17·5)	60·4 (29·1)	72·8 (19·9)	87·2 (7·7)	87·8 (7·5)	86·3 (8·0)	81·7 (11·8)[m]
**ECAS**	87·2 (20·9)	87·2 (16·5)	77·6 (26·4)	94·7 (20·8)	80·0 (31·7)[g]	88·0 (28·7)	57·0 (36·2)[t]	89·2 (24·6)	99·4 (12·7)	99·5 (13·8)	99·4 (11·2)	91·1 (20·3)
**FTD-FRS**	19·0 (10·4)	18·6 (10·8)	21·2 (10·2)	18·1 (9·9)	17·3 (12·1)	19·6 (12·7)	21·4 (11·5)	14·3 (12·0)	15·9 (10·0)	15·6 (9·6)	16·1 (10·6)	12·9 (10·0)
**Serum NF-L level**	47·1 (13·1)	45.3 (27.4)	60.1 (41.4)	40.1 (21.0)	51·1 (29·6)	52.5 (26.5)	36.5 (20.5)	55.9 (33.1)	32·6 (12·4)	32.6 (12.4)	··	29·5 (19·7)
**CSF Aβ1-42 level**	824 (316)	1124 (235)	768 (320)	631 (17.8)	572 (263)	748 (223)	351 (69.4)	··	··	··	··	··
**CSF T-tau level**	284 (79·1)	300 (111)	274 (79.3)	297 (44.9)	499 (272)	277 (76.0)	777 (119)	··	··	··	··	··
**CSF T-tau: Aβ1-42 ratio**	0·4 (0·1)	0·3 (0·1)	0·4 (0·2)	0·5 (0·1)	1·2 (1·0)	0·4 (0·1)	2·3 (0·5)	··	··	··	··	··

a. Tukey post hoc test (TPH) adjusted $p < 0·001$ vs APS-all; b. TPH adjusted $p < 0·05$ vs APS-all; c. TPH adjusted $p < 0·01$ vs PSP-all; d. TPH adjusted $p < 0·05$ vs PSP-all; e. TPH adjusted $p < 0·0001$ vs MSA-all; f. TPH adjusted $p < 0·001$ vs MSA-all; g. TPH adjusted $p < 0·01$ vs MSA-all; h. TPH adjusted $p < 0·05$ vs MSA-all; i. TPH adjusted $p < 0·0001$ vs CBS-all; j. TPH adjusted $p < 0·05$ vs CBS-all; k. TPH adjusted $p < 0·0001$ vs PSP-all; l. TPH adjusted $p < 0·001$ vs PSP-all; m. TPH adjusted $p < 0·05$ vs PSP-all; n. TPH adjusted $p < 0·01$ vs PSP-cortical; o. TPH adjusted $p < 0·05$ vs PSP-cortical; p. TPH adjusted $p < 0·001$ vs PSP-subcortical; q. TPH adjusted $p < 0·01$ vs PSP-subcortical; r. TPH adjusted $p < 0·05$ vs PSP-subcortical; s. TPH adjusted $p < 0·05$ vs CBS-AD; t. TPH adjusted $p < 0·05$ vs CBS-IDT. Abbreviations: 4RT = 4-repeat tau, Aβ1-42 = β-amyloid 1-42, AD = Alzheimer disease, ACE III = Addenbrookes Cognitive Examination III, CBD-FS = Corticobasal Degeneration Functional Scale, CBI-R = Cambridge Behavioural Inventory Revised, CBS = corticobasal syndrome , ECAS = Edinburgh Cognitive and Behavioural Screen, FTD-FRS = Frontotemporal Dementia Rating Scale, IDT = Indeterminate, MoCA = Montreal Cognitive Assessment, mPSPRS = modified Progressive Supranuclear Palsy Rating Scale, MSA = multiple system atrophy, MSA QoL = MSA Quality of life scale, NF-L = neurofilament light chain, PSP = progressive supranuclear palsy, PSP QoL = PSP Quality of life scale, PSPRS = PSP rating scale, SEADL = Schwab and England Activities of Daily Living Scale, UPDRS = Unified Parkinson's Disease Rating Scale, UMSARS = Unified MSA Rating Scale

<u>Supplementary Table 2</u>: Completion rate of each variable at each assessment

	Baseline (raw)	Baseline (% of whole)	Six months (raw)	Six months (% of whole)	Twelve months (raw)	Twelve months (% of whole)	Twelve months as % of baseline	Less than twelve months follow-up mean (SD) baseline score	Twelve months follow-up mean (SD) baseline score	p-value
**CBD-FS**	76	31	57	23	76	31	100	42·8 (19·6)	35·8 (18·2)	0·14
**SEADL**	239	98	141	58	130	53	54	49·4 (23·4)	60·0 (21·4)	0·00060
**Milestones**	235	97	137	56	131	54	56	2·8 (1·8)	2·7 (1·8)	0·77
**MSA-QoL**	38	56	22	32	13	19	34	42·6 (21·3)	44·2 (12·7)	0·79
**PSP-QoL**	154	63	86	35	79	33	51	72·0 (32·1)	55·8 (30·9)	0·0067
**UPDRS – Part 2**	198	81	116	48	103	42	52	25·2 (10·2)	21·4 (9·2)	0·028
**UPDRS – Part 3**	179	74	111	46	97	40	54	50·2 (19·6)	40·4 (18·3)	0·0012
**PSPRS**	179	74	111	46	98	40	55	35·3 (14·3)	28·4 (13·1)	0·0028
**UMSARS**	60	88	33	49	30	44	50	44·5 (15·9)	45·4 (14·6)	0·81
**UPDRS – Part 1**	198	81	116	48	103	42	52	11·7 (5·0)	9·8 (4·9)	0·024
**CBI-R**	199	82	119	49	100	41	50	45·6 (28·8)	39·1 (27·2)	0·11
**MoCA**	224	92	131	54	128	53	57	21·1 (6·2)	23·4 (4·8)	0·0036
**ACE III**	207	85	123	51	119	49	57	75·0 (17·6)	82·6 (12·7)	0·00043
**ECAS**	174	72	115	47	89	37	51	82·9 (22·6)	91·4 (21·9)	0·0096
**FTD-FRS**	175	72	103	42	88	36	50	18·9 (10·9)	17·0 (10·5)	0·22
**Blood**	145	60	··	··	··	··	··	··	··	··
**CSF**	36	15	··	··	··	··	··	··	··	··
**Genetics**	148	61	··	··	··	··	··	··	··	··
**MRI**	104	43	··	··	59	24	48	··	··	··

Abbreviations: 4RT = 4-repeat tau, AD = Alzheimer disease, ACE III = Addenbrookes Cognitive Examination III, CBD-FS = Corticobasal Degeneration Functional Scale, CBI-R = Cambridge Behavioural Inventory Revised, CBS = corticobasal syndrome , ECAS = Edinburgh Cognitive and Behavioural Screen, FTD-FRS = Frontotemporal Dementia Rating Scale, IDT = Indeterminate, MoCA = Montreal Cognitive Assessment, mPSPRS = modified Progressive Supranuclear Palsy Rating Scale, MSA = multiple system atrophy, MSA QoL = MSA Quality of life scale, PSP = progressive supranuclear palsy, PSP QoL = PSP Quality of life scale, PSPRS = PSP rating scale, SD = standard deviation, SEADL = Schwab and England Activities of Daily Living Scale, UPDRS = Unified Parkinson's Disease Rating Scale, UMSARS = Unified MSA Rating Scale

Supplementary Table 3: Annualised rates of progression in Activity of Daily Living, Quality of Life, Motor Examination, and Neuropsychiatric markers by Final diagnosis

	PSP				CBS				MSA			IDT
	**All (n=117)**	**RS (n=57)**	**Cortical (n=28)**	**Subcortical (n=32)**	**All (n=42)**	**4RT (n=10)**	**AD (n=10)**	**Unknown (n=22)**	**All (n=68)**	**Parkinsonism (n=41)**	**Cerebellar (n=26)**	**(n=16)**
**CBD-FS**	15·8 (10·0)	14·2 (8·3)	19·9 (13·8)	14·1 (6·7)	16·4 (7·6)	16·3 (1·9)	15·2 (14·8)	17·2 (3·1)	··	··	··	12·9 (8·8)
**SEADL**	-11·0 (15·5)	-11·1 (19·0)	-10·5 (8·7)	-11·2 (13·7)	-14·3 (10·7)	-16·5 (10·7)	-14·9 (13·9)	-13·0 (9·4)	-12·1 (11·3)	-10·5 (7·8)	-14·7 (15·2)	-9·1 (13·1)
**Milestones**	1·1 (1·5)	0·9 (1·6)	1·2 (1·9)	1·3 (1·1)	0·7 (1·0)	0·6 (1·5)	0·7 (0·6)	0·7 (0·9)	0·9 (1·0)	0·9 (1·1)	0·9 (0·9)	0·6 (1·1)
**MSA QoL**	··	··	··	··	··	··	··	··	1·5 (11·3)	-0·02 (11·6)	3·3 (10·9)	··
**PSP QoL**	23·0 (20·7)	23·3 (18·8)	28·5 (24·3)	18·0 (19·9)	23·9 (22·6)	37·0 (27·3)	27·8 (18·6)	14·5 (17·3)	··	··	··	15·6 (16·8)
**UPDRS II**	6·9 (6·2)	7·4 (6·5)	8·6 (5·5)	4·6 (5·8)	7·9 (6·4)	9·6 (6·9)	9·6 (7·7)	6·2 (5·4)	5·3 (6·1)	5·6 (4·5)	4·9 (7·9)	3·7 (4·4)
**UPDRS III**	9·3 (12·0)	9·0 (12·9)	10·9 (10·1)	8·4 (12·0)	9·0 (7·1)	10·8 (7·8)	10·4 (8·4)	7·5 (6·1)	··	··	··	4·9 (11·6)
**PSPRS**	9·0 (10·1)	9·7 (11·3)	10·9 (9·3)	6·3 (8·0)	9·9 (7·3)	10·3 (10·8)	10·6 (5·5)	9·4 (6·4)	··	··	··	4·2 (7·1)
**mPSPRS**	3·1 (2·8)	3·2 (3·0)	3·2 (2·9)	2·6 (2·2)	3·3 (2·4)	3·6 (2·3)	3·4 (2·7)	3·2 (2·4)	··	··	··	1·7 (2·2)
**UMSARS**	··	··	··	··	··	··	··	··	10·6 (7·5)	12·0 (7·0)	8·6 (8·0)	··
**UPDRS I**	1·2 (3·8)	0·8 (4·2)	2·4 (4·5)	0·9 (2·3)	1·6 (3·0)	2·3 (3·6)	3·3 (3·7)	0·5 (1·9)	0·5 (3·3)	0·4 (3·2)	0·6 (3·6)	0·4 (2·8)
**CBI-R**	12·7 (19·0)	15·2 (19·2)	18·1 (19·4)[a]	3·6 (15·5)	15·2 (17·0)	18·6 (14·4)	19·7 (14·8)	11·6 (19·0)	11·3 (13·8)	13·0 (12·8)	8·9 (15·0)	8·9 (11·2)
**MoCA**	-0·9 (3·7)	-0·8 (3·0)	-1·6 (3·9)	-0·6 (4·6)	-1·5 (2·8)	-1·0 (1·6)	-1·8 (2·6)	-1·6 (3·3)	-0·4 (2·6)	-0·7 (2·3)	0·2 (2·9)	0·2 (2·5)
**ACE III**	-1·7 (7·1)	-1·6 (4·0)	-3·9 (9·1)	-0·3 (9·3)	-4·0 (6·3)	-4·1 (5·9)	-5·8 (8·5)	-2·9 (5·1)	-1·2 (5·6)	-0·8 (6·0)	-1·8 (4·7)	-1·9 (3·1)
**ECAS**	-2·7 (12·3)	-4·7 (9·5)	-3·2 (6·5)	1·4 (18·7)	-3·4 (7·5)	-1·5 (6·0)	-1·7 (7·4)	-5·7 (8·1)	-4·3 (8·8)	-3·7 (7·3)	-5·2 (11·1)	2·9 (10·9)
**FTD-FRS**	4·0 (8·2)	5·0 (7·6)	4·5 (7·9)	1·7 (9·2)	4·9 (10·6)	6·0 (17·3)	3·4 (5·0)	5·1 (9·3)	2·5 (5·3)	3·5 (3·6)	1·6 (6·5)	3·5 (1·9)

a. Tukey post hoc test (TPH) adjusted $p < 0·05$ vs PSP-subcortical. Variables with fewer than 5 responses per group have been removed. Abbreviations: 4RT = 4-repeat tau, AD = Alzheimer disease, ACE III = Addenbrookes Cognitive Examination III, CBD-FS = Corticobasal Degeneration Functional Scale, CBI-R = Cambridge Behavioural Inventory Revised, CBS = corticobasal syndrome , ECAS = Edinburgh Cognitive and Behavioural Screen, FTD-FRS = Frontotemporal Dementia Rating Scale, IDT = Indeterminate, MoCA = Montreal Cognitive Assessment, mPSPRS = modified Progressive Supranuclear Palsy Rating Scale, MSA = multiple system atrophy, MSA QoL = MSA Quality of life scale, PSP = progressive supranuclear palsy, PSP QoL = PSP Quality of life scale, PSPRS = PSP rating scale, SEADL = Schwab and England Activities of Daily Living Scale, UPDRS = Unified Parkinson's Disease Rating Scale, UMSARS = Unified MSA Rating Scale.

<u>Supplementary Table 4:</u> Annualised rates of progression in neuroimaging markers (percentage change from baseline) by Final diagnosis

	PSP				CBS				MSA			IDT
	**All (n=28)**	**RS (n=19)**	**Cortical (n=0)**	**Subcortical (n=9)**	**All (n=8)**	**4RT (n=3)**	**AD (n=2)**	**Unknown (n=3)**	**All (n=17)**	**Parkinsonism (n=12)**	**Cerebellar (n=5)**	**(n=5)**
**Temporal lobe**	-2·8 (2·3)	-2·6 (2·7)	··	-3·0 (1·3)	-1·9 (4·4)	0·02 (0·4)	-4·6 (9·3)	-1·3 (1·8)	-3·2 (2·6)	-3·4 (2·4)	-2·4 (3·3)	-3·1 (1·6)
**Cingulate**	-2·0 (2·1)	-2·3 (2·4)	··	-1·4 (1·2)	-1·9 (2·9)	-1·5 (1·1)	-2·7 (6·6)	-1·5 (1·1)	-3·0 (2·7)	-2·6 (1·8)	-4·0 (4·2)	-2·6 (2·0)
**Insula**	-1·2 (2·5)	-0·9 (2·6)	··	-2·0 (2·1)	-1·0 (3·2)	2·3 (1·9)	-2·5 (5·0)	-2·2 (1·3)	-2·3 (3·2)	-1·9 (2·4)	-3·3 (4·8)	-1·9 (1·9)
**Occipital lobe**	-2·5 (2·6)	-2·3 (2·7)	··	-2·9 (2·5)	-1·7 (4·7)	1·6 (0·2)	-2·5 (9·8)	-3·4 (1·5)	-2·6 (3·3)	-3·0 (3·3)	-1·7 (3·5)	-3·0 (3·8)
**Frontal lobe**	-3·8 (3·1)	-4·0 (3·5)	··	-3·3 (2·1)	-2·1 (4·4)	-0·7 (0·06)	-2·2 (11·0)	-2·8 (0·8)	-4·4 (3·9)	-3·9 (3·4)	-5·5 (5·1)	-3·7 (1·7)
**Parietal lobe**	-3·0 (2·9)	-2·9 (3·0)	··	-3·1 (2·9)	-2·3 (6·3)	-0·4 (0·04)	-0·9 (14·0)	-4·5 (2·0)	-3·9 (3·4)	-4·0 (3·1)	-3·7 (4·4)	-2·6 (2·0)
**Central structures**	-1·9 (3·5)	-1·9 (4·2)	··	-2·1 (1·5)	-2·8 (2·2)	-0·7 (1·2)	-3·3 (0·5)	-3·9 (2·6)	-3·6 (3·0)	-3·1 (2·1)	-4·8 (4·6)	-1·0 (1·2)
**Ventricles**	11·0 (6·7)	11·8 (7·6)	··	9·1 (4·1)	13·4 (10·1)	1·1 (2·9)	22·0 (3·0)	15·9 (8·1)	15·9 (12·3)	13·6 (6·3)	21·3 (21·2)	9·2 (6·7)
**Cerebellum**	-3·4 (4·1)	-3·4 (4·7)	··	-3·5 (2·5)	-1·9 (3·7)	-0·6 (2·0)	-5·5 (5·1)	-0·4 (2·7)	-5·6 (3·9)	-6·1 (3·5)	-4·4 (5·1)	-4·5 (4·0)
**Medulla**	0·9 (7·1)	0·4 (5·9)	··	2·0 (9·7)	-0·3 (2·5)	-1·0 (3·3)	2·5 (1·2)	-1·7 (1·3)	-0·6 (3·0)	-0·2 (3·4)	-1·4 (2·1)	0·8 (1·8)
**Pons**	-2·2 (3·1)[b]	-2·7 (3·5)	··	-1·0 (1·6)	-0·2 (1·1)[a]	0·4 (0·8)	0·6 (0·1)	-1·2 (0·8)	-7·1 (4·5)	-6·9 (4·9)	-7·7 (4·0)	-0·3 (1·4)[c]
**Midbrain**	-0·5 (4·9)	-1·0 (5·3)	··	0·7 (3·9)	-0·2 (2·1)	-2·1 (1·8)	2·2 (1·9)	-0·6 (0·7)	-2·8 (3·6)	-1·7 (2·2)	-5·5 (4·9)	0·5 (4·5)
**Midbrain:Pons ratio**	0·9 (7·1)[c]	0·4 (5·9)	··	2·0 (9·7)	-0·3 (2·5)[b]	-1·0 (3·3)	2·5 (1·2)	-1·7 (1·3)	-0·6 (3·0)	-0·2 (3·4)	-1·4 (2·1)	0·8 (1·8)

a. Tukey post hoc test (TPH) adjusted $p < 0·001$ vs MSA-all; b. TPH adjusted $p < 0·01$ vs MSA-all; c. TPH adjusted $p < 0·05$ vs MSA-all. Abbreviations: 4RT = 4-repeat tau, AD = Alzheimer disease, CBS = corticobasal syndrome, IDT = Indeterminate, MSA = multiple system atrophy, PSP = progressive supranuclear palsy

Supplementary Table 5: Annualised rates of progression in Activity of Daily Living, Quality of Life, Motor Examination, and Neuropsychiatric markers by Intention to Treat

	PSP				CBS				MSA			IDT
	**All (n=99)**	**RS (n=52)**	**Cortical (n=12)**	**Subcortical (n=35)**	**All (n=60)**	**4RT (n=16)**	**AD (n=12)**	**Unknown (n=32)**	**All (n=64)**	**Parkinsonism (n=38)**	**Cerebellar (n=26)**	**(n=20)**
**CBD-FS**	12·5 (10·0)	11·8 (12·1)	15·8 (8·5)	8·4 (6·2)	9·6 (12·0)	11·1 (19·0)	4·2 (5·7)	23·3 (10·2)	··	··	··	13·4 (9·0)
**SEADL**	-12·0 (21·1)	-15·9 (19·4)	-14·5 (7·5)	-5·2 (25·7)	-13·3 (13·3)	-10·7 (16·4)	-8·4 (9·7)	-16·5 (14·0)	-8·8 (15·8)	-7·2 (10·8)	-10·4 (19·0)	-3·4 (21·4)
**Milestones**	0·9 (1·3)	1·0 (1·5)	0·9 (0·9)	0·9 (1·2)	0·8 (1·3)	1·5 (2·4)	0·8 (1·0)	0·7 (1·2)	0·7 (1·0)	0·7 (1·1)	0·6 (0·9)	0·4 (1·1)
**MSA QoL**	··	··	··	··	··	··	··	··	3·8 (14·0)	-1·9 (13·2)	9·8 (13·8)	··
**PSP QoL**	14·8 (20·1)	18·4 (22·3)	16·7 (19·8)	9·2 (17·1)	14·9 (20·2)	15·7 (25·9)	8·6 (12·3)	22·0 (23·8)	··	··	··	18·6 (15·8)
**UPDRS II**	5·7 (6·4)	7·1 (6·1)	11·6 (6·6)	1·9 (5·7)	5·8 (5·9)	4·9 (7·0)	5·4 (5·7)	7·1 (6·4)	4·4 (7·0)	3·4 (4·5)	5·6 (9·1)	3·8 (3·7)
**UPDRS III**	9·3 (13·2)	9·6 (13·7)	13·3 (10·3)	7·3 (13·2)	7·5 (12·2)	8·2 (21·2)	7·3 (6·2)	6·5 (12·5)	··	··	··	9·6 (14·2)
**PSPRS**	9·9 (8·9)	11·6 (9·1)	13·9 (8·0)	5·9 (8·0)	9·2 (9·1)	11·0 (16·9)	7·0 (4·5)	10·4 (8·7)	··	··	··	4·2 (6·9)
**mPSPRS**	3·6 (3·2)	4·0 (3·3)	5·8 (2·9)	2·3 (2·6)	2·6 (3·0)	2·3 (4·4)	1·9 (1·9)	3·4 (3·2)	··	··	··	1·6 (2·8)
**UMSARS**	··	··	··	··	··	··	··	··	9·5 (8·2)	11·2 (8·6)	7·0 (7·4)	··
**UPDRS I**	0·6 (3·7)	0·4 (4·0)	3·6 (3·3)	0·2 (2·9)	1·6 (3·5)	1·6 (4·5)	1·7 (2·8)	1·7 (3·8)	0·04 (4·1)	-0·8 (3·8)	1·2 (4·3)	1·5 (3·8)
**CBI-R**	8·8 (20·1)	15·2 (21·8)	7·3 (12·9)	-1·1 (15·3)	12·9 (18·4)	14·4 (24·3)	7·7 (10·1)	18·1 (21·7)	12·0 (22·4)	17·8 (22·5)	5·6 (21·0)	10·2 (13·8)
**MoCA**	-0·9 (3·5)	-0·6 (3·0)	-3·7 (2·3)	-0·6 (4·3)	-1·5 (3·5)	-2·7 (5·1)	-2·4 (3·3)	-0·7 (3·0)	0·2 (3·1)	-0·2 (2·6)	0·7 (3·7)	-0·9 (3·0)
**ACE III**	-1·7 (5·3)	-1·4 (5·3)	-5·2 (6·9)	-1·1 (4·7)	-7·1 (10·2)	-8·1 (16·6)	-13·5 (11·9)	-5·0 (6·6)	-1·3 (6·9)	-0·8 (7·1)	-2·2 (6·6)	-2·3 (4·0)
**ECAS**	-3·1 (12·9)	-4·5 (10·7)	-12·9 (13·6)	1·4 (15·2)	-3·3 (10·8)	2·7 (11·1)	-6·3 (11·9)	-4·9 (10·3)	-4·8 (11·2)	-3·6 (9·4)	-6·2 (12·7)	2·1 (12·0)
**FTD-FRS**	2·7 (8·7)	4·3 (8·5)	4·6 (9·1)	-1·7 (8·1)	2·1 (10·0)	5·4 (15·1)	2·2 (8·7)	1·2 (8·9)	1·1 (8·2)	1·8 (5·4)	1·0 (9·2)	3·0 (3·3)

Variables with fewer than 5 responses per group have been removed. Abbreviations: 4RT = 4-repeat tau, AD = Alzheimer disease, ACE III = Addenbrookes Cognitive Examination III, CBD-FS = Corticobasal Degeneration Functional Scale, CBI-R = Cambridge Behavioural Inventory Revised, CBS = corticobasal syndrome , ECAS = Edinburgh Cognitive and Behavioural Screen, FTD-FRS = Frontotemporal Dementia Rating Scale, IDT = Indeterminate, MoCA = Montreal Cognitive Assessment, mPSPRS = modified Progressive Supranuclear Palsy Rating Scale, MSA = multiple system atrophy, MSA QoL = MSA Quality of life scale, PSP = progressive supranuclear palsy, PSP QoL = PSP Quality of life scale, PSPRS = PSP rating scale, SEADL = Schwab and England Activities of Daily Living Scale, UPDRS = Unified Parkinson's Disease Rating Scale, UMSARS = Unified MSA Rating Scale

Supplementary Table 6: Annualised rates of progression in neuroimaging markers (percentage change from baseline) by Intention to Treat

	PSP				CBS				MSA			IDT
	**All (n=29)**	**RS (n=18)**	**Cortical (n=1)**	**Subcortical (n=11)**	**All (n=9)**	**4RT (n=2)**	**AD (n=1)**	**Unknown (n=6)**	**All (n=15)**	**Parkinsonism (n=10)**	**Cerebellar (n=5)**	**(n=5)**
**Temporal lobe**	-2·8 (2·3)	-2·9 (2·4)	··	-2·8 (2·2)	-1·8 (4·0)	··	··	-0·4 (1·6)	-3·3 (2·8)	-3·7 (2·5)	-2·4 (3·3)	-2·8 (1·3)
**Cingulate**	-2·0 (2·1)	-2·4 (2·3)	··	-1·3 (1·5)	-2·4 (2·9)	··	··	-1·8 (2·5)	-2·8 (2·8)	-2·2 (1·8)	-4·0 (4·2)	-2·9 (1·9)
**Insula**	-1·3 (2·5)	-1·0 (2·8)	··	-1·9 (2·0)	-1·5 (3·2)	··	··	-0·7 (3·0)	-2·2 (3·4)	-1·7 (2·6)	-3·3 (4·8)	-1·3 (1·2)
**Occipital lobe**	-2·7 (2·8)	-2·7 (3·0)	··	-2·7 (2·6)	-1·1 (4·4)	··	··	-1·0 (2·4)	-3·0 (3·3)	-3·6 (3·2)	-1·7 (3·5)	-2·0 (2·1)
**Frontal lobe**	-3·8 (3·0)	-4·4 (3·2)	··	-2·9 (2·5)	-3·0 (4·4)	··	··	-1·7 (4·1)	-4·2 (4·1)	-3·6 (3·6)	-5·5 (5·1)	-3·3 (1·9)
**Parietal lobe**	-3·0 (2·9)	-3·2 (3·0)	··	-2·8 (2·8)	-2·6 (5·9)	··	··	-0·5 (5·1)	-3·9 (3·6)	-4·0 (3·4)	-3·7 (4·4)	-2·2 (1·7)
**Central structures**	-1·9 (3·4)	-3·0 (1·9)	··	-0·03 (4·6)	-3·1 (2·2)	··	··	-3·2 (2·6)	-3·3 (3·0)	-2·5 (1·7)	-4·8 (4·6)	-2·3 (2·8)
**Ventricles**	10·7 (6·7)	12·4 (7·5)	··	8·0 (4·0)	13·3 (9·2)	··	··	12·0 (9·5)	15·5 (13·0)	12·6 (6·0)	21·3 (21·2)	13·2 (9·1)
**Cerebellum**	-3·7 (4·3)	-4·1 (3·9)	··	-3·0 (5·0)	-2·1 (3·7)	··	··	-1·6 (2·4)	-5·9 (4·0)	-6·6 (3·4)	-4·4 (5·1)	-3·0 (1·6)
**Medulla**	1·0 (7·0)	0·3 (5·9)	··	2·1 (8·7)	0·3 (2·7)	··	··	0·4 (2·5)	-1·0 (3·0)	-0·7 (3·4)	-1·4 (2·1)	0·4 (1·8)
**Pons**	-2·2 (3·0)[a]	-3·3 (2·9)	··	-0·3 (2·3)	-2·4 (4·7)[a]	··	··	-3·2 (5·3)	-6·7 (4·7)	-6·3 (5·2)	-7·7 (4·0)	-0·4 (1·5)[a]
**Midbrain**	-0·5 (4·8)	-2·4 (2·6)	··	2·8 (5·9)	-1·2 (2·9)	··	··	-2·2 (2·4)	-2·6 (3·7)	-1·2 (1·9)	-5·5 (4·9)	0·3 (4·5)
**Midbrain:Pons ratio**	1·0 (7·0)[a]	0·3 (5·9)	··	2·1 (8·7)	0·3 (2·7)[a]	··	··	0·4 (2·5)	-1·0 (3·0)	-0·7 (3·4)	-1·4 (2·1)	0·4 (1·8)

Abbreviations: 4RT = 4-repeat tau, AD = Alzheimer disease, CBS = corticobasal syndrome, IDT = Indeterminate, MSA = multiple system atrophy, PSP = progressive supranuclear palsy
a. Tukey post hoc test (TPH) adjusted p < 0·05 vs MSA-all

Supplementary Table 7: Sample sizes required for a 2-arm, 1-year follow-up therapeutic trial to detect 25% and 50% change by Final diagnosis

	PSP					CBS					MSA				
		25% change		50% change			25% change		50% change			25% change		50% change	
	**Difference mean (SD)**	**Effect size**	**Sample size[a]**	**Effect size**	**Sample size[a]**	**Difference mean (SD)**	**Effect size**	**Sample size[a]**	**Effect size**	**Sample size[a]**	**Difference mean (SD)**	**Effect size**	**Sample size[a]**	**Effect size**	**Sample size[a]**
**CBD-FS**	12·3 (12·5)	0·246	260 (347)	0·491	66 (88)	8·6 (10·9)	0·198	401 (535)	0·397	101 (135)	··	··	··	··	··
**SEADL**	-12·6 (19·2)	0·163	592 (789)	0·326	149 (199)	-12·7 (12·4)	0·256	240 (320)	0·512	61 (81)	-9·6 (16·8)	0·143	769 (1025)	0·286	193 (257)
**Milestones**	1·1 (1·4)	0·194	418 (557)	0·389	105 (140)	0·5 (1·0)	0·124	1022 (1363)	0·248	256 (341)	0·8 (1·1)	0·171	538 (717)	0·341	136 (181)
**MSA-QoL**	··	··	··	··	··	··	··	··	··	··	3·8 (14·2)	0·066	>1500	0·133	888 (1184)
**PSP-QoL**	15·0 (21·2)	0·177	502 (669)	0·354	126 (168)	13·4 (18·5)	0·181	480 (640)	0·362	121 (161)	··	··	··	··	··
**UPDRS II**	5·4 (6·4)	0·213	347 (463)	0·425	88 (117)	6·2 (6·1)	0·254	244 (325)	0·508	62 (83)	5·2 (7·1)	0·183	470 (627)	0·366	118 (157)
**UPDRS III**	9·5 (14·3)	0·167	564 (752)	0·334	142 (189)	6·3 (8·5)	0·186	455 (607)	0·373	114 (152)	··	··	··	··	··
**PSPRS**	10·2 (9·5)	0·267	221 (295)	0·533	56 (75)	8·1 (7·4)	0·275	209 (279)	0·550	53 (71)	··	··	··	··	··
**mPSPRS**	3·8 (3·5)	0·266	223 (297)	0·532	56 (75)	3·3 (3·4)	0·247	258 (344)	0·495	65 (87)	··	··	··	··	··
**UMSARS**	··	··	··	··	··	··	··	··	··	··	9·5 (8·4)	0·283	197 (263)	0·567	50 (67)
**UPDRS I**	0·7 (3·9)	0·047	>1500	0·094	>1500	1·6 (3·0)	0·136	850 (1133)	0·272	213 (284)	0·8 (4·2)	0·049	>1500	0·097	>1500
**CBI-R**	9·3 (21·0)	0·111	1275 (1700)	0·223	317 (423)	11·9 (17·4)	0·170	544 (725)	0·340	137 (183)	11·9 (20·4)	0·145	748 (997)	0·291	186 (248)
**MoCA**	-1·1 (3·6)	0·078	>1500	0·156	646 (861)	-1·5 (2·9)	0·133	888 (1184)	0·266	223 (297)	0·2 (2·9)	0·020	>1500	0·040	>1500
**ACE III**	-2·1 (7·4)	0·070	>1500	0·140	802 (1069)	-7·2 (8·0)	0·226	308 (411)	0·452	78 (104)	-1·3 (6·6)	0·048	>1500	0·096	>1500
**ECAS**	-3·0 (13·5)	0·056	>1500	0·112	1252 (1669)	-4·4 (10·4)	0·106	1398 (1864)	0·212	350 (467)	-4·3 (11·2)	0·095	>1500	0·189	440 (587)
**FTD-FRS**	2·4 (8·9)	0·067	>1500	0·134	875 (1167)	0·8 (10·1)	0·020	>1500	0·039	>1500	1·4 (7·5)	0·047	>1500	0·093	>1500

a. Sample sizes are calculated per group, based on a significance level of 5% and a power of 80%; an approximation adjusting for a drop-out rate of 25% using the formula (e.g. 233/0.75=311) is presented in parentheses.
Abbreviations: 4RT = 4-repeat tau, AD = Alzheimer disease, ACE III = Addenbrookes Cognitive Examination III, CBD-FS = Corticobasal Degeneration Functional Scale, CBI-R = Cambridge Behavioural Inventory Revised, CBS = corticobasal syndrome , ECAS = Edinburgh Cognitive and Behavioural Screen, FTD-FRS = Frontotemporal Dementia Rating Scale, IDT = Indeterminate, MoCA = Montreal Cognitive Assessment, mPSPRS = modified Progressive Supranuclear Palsy Rating Scale, MSA = multiple system atrophy, MSA QoL = MSA Quality of life scale, PSP = progressive supranuclear palsy, PSP QoL = PSP Quality of life scale, PSPRS = PSP rating scale, SD = standard deviation, SEADL = Schwab and England Activities of Daily Living Scale, UPDRS = Unified Parkinson's Disease Rating Scale, UMSARS = Unified MSA Rating Scale.
Variables with fewer than 5 responses per group have been removed.

Supplementary Table 8: Sample sizes required for a 2-arm, 1-year follow-up therapeutic trial to detect 25% and 50% change by Final diagnosis

	PSP					CBS					MSA				
		25% change		50% change			25% change		50% change			25% change		50% change	
	**Percent difference mean (SD)**	**Effect size**	**Sample size[a]**	**Effect size**	**Sample size[a]**	**Difference mean (SD)**	**Effect size**	**Sample size[a]**	**Effect size**	**Sample size[a]**	**Difference mean (SD)**	**Effect size**	**Sample size[a]**	**Effect size**	**Sample size[a]**
**Temporal lobe**	-2·8 (2·3)	0·260	233 (311)	0·520	59 (79)	-1·9 (4·4)	0·168	557 (743)	0·336	140 (187)	-3·2 (2·6)	0·252	248 (331)	0·504	63 (84)
**Cingulate**	-2·0 (2·1)	0·256	240 (320)	0·513	61 (81)	-1·9 (2·9)	0·205	374 (499)	0·409	95 (127)	-3·0 (2·7)	0·346	132 (176)	0·693	34 (45)
**Insula**	-1·2 (2·5)	0·146	737 (983)	0·292	185 (247)	-1·0 (3·2)	0·107	1372 (1829)	0·214	344 (459)	-2·3 (3·2)	0·226	308 (411)	0·451	78 (104)
**Occipital lobe**	-2·5 (2·6)	0·204	378 (504)	0·408	95 (127)	-1·7 (4·7)	0·113	1230 (1640)	0·225	311 (415)	-2·6 (3·3)	0·153	672 (896)	0·307	168 (224)
**Frontal lobe**	-3·8 (3·1)	0·334	142 (189)	0·668	36 (48)	-2·1 (4·4)	0·161	607 (809)	0·322	152 (203)	-4·4 (3·9)	0·372	114 (152)	0·744	29 (39)
**Parietal lobe**	-3·0 (2·9)	0·284	196 (261)	0·568	50 (67)	-2·3 (6·3)	0·143	769 (1025)	0·286	193 (257)	-3·9 (3·4)	0·336	140 (187)	0·671	36 (48)
**Central structures**	-1·9 (3·5)	0·123	1039 (1385)	0·245	262 (349)	-2·8 (2·2)	0·342	135 (180)	0·684	35 (47)	-3·6 (3·0)	0·471	72 (96)	0·942	19 (25)
**Ventricles**	11·0 (6·7)	0·426	87 (116)	0·852	23 (31)	13·4 (10·1)	0·304	171 (228)	0·609	43 (57)	15·9 (12·3)	0·274	210 (280)	0·548	53 (71)
**Cerebellum**	-3·4 (4·1)	0·231	295 (393)	0·461	75 (100)	-1·9 (3·7)	0·159	622 (829)	0·319	155 (207)	-5·6 (3·9)	0·415	92 (123)	0·829	24 (32)
**Medulla**	0·9 (7·1)	0·025	>1500	0·050	>1500	-0·3 (2·5)	0·004	>1500	0·007	>1500	-0·6 (3·0)	0·034	>1500	0·067	>1500
**Pons**	-2·2 (3·1)	0·232	293 (391)	0·464	74 (99)	-0·2 (1·1)	0·029	>1500	0·059	>1500	-7·1 (4·5)	0·387	106 (141)	0·774	27 (36)
**Midbrain**	-0·5 (4·9)	0·029	>1500	0·059	>1500	-0·2 (2·1)	0·007	>1500	0·014	>1500	-2·8 (3·6)	0·296	180 (240)	0·591	46 (61)
**Midbrain:Pons ratio**	0·9 (7·1)	0·110	1298 (1731)	0·220	325 (433)	-0·3 (2·5)	0·015	>1500	0·030	>1500	-0·6 (3·0)	0·293	184 (245)	0·586	47 (63)

a. Sample sizes are calculated per group, based on a significance level of 5% and a power of 80%; an approximation adjusting for a drop-out rate of 25% using the formula (e.g. 233/0.75=311) is presented in parentheses.
Abbreviations: 4RT = 4-repeat tau, AD = Alzheimer disease, CBS = corticobasal syndrome, IDT = Indeterminate, MSA = multiple system atrophy, PSP = progressive supranuclear palsy

Supplementary Table 9: Sample sizes required for a 2-arm, 1-year follow-up therapeutic trial to detect 25% and 50% change by Intention to Treat

	PSP					CBS					MSA				
		25% change		50% change			25% change		50% change			25% change		50% change	
	Difference mean (SD)	Effect size	Sample size[a]	Effect size	Sample size[a]	Difference mean (SD)	Effect size	Sample size[a]	Effect size	Sample size[a]	Difference mean (SD)	Effect size	Sample size[a]	Effect size	Sample size[a]
**CBD-FS**	12·5 (10·0)	0·313	161 (215)	0·627	41 (55)	9·6 (12·0)	0·201	390 (520)	0·402	98 (131)	··	··	··	··	··
**SEADL**	-12·0 (21·1)	0·141	791 (1055)	0·283	197 (263)	-13·3 (13·3)	0·251	250 (333)	0·502	63 (84)	-8·8 (15·8)	0·139	813 (1084)	0·278	204 (272)
**Milestones**	0·9 (1·3)	0·180	485 (647)	0·359	123 (164)	0·8 (1·3)	0·148	718 (957)	0·297	179 (239)	0·7 (1·0)	0·168	557 (743)	0·336	140 (187)
**MSA-QoL**	··	··	··	··	··	··	··	··	··	··	3·8 (14·0)	0·067	>1500	0·134	875 (1167)
**PSP-QoL**	14·8 (20·1)	0·185	460 (613)	0·369	116 (155)	14·9 (20·2)	0·184	465 (620)	0·369	116 (155)	··	··	··	··	··
**UPDRS II**	5·7 (6·4)	0·221	322 (429)	0·443	81 (108)	5·8 (5·9)	0·243	267 (356)	0·485	68 (91)	4·4 (7·0)	0·155	654 (872)	0·310	164 (219)
**UPDRS III**	9·3 (13·2)	0·176	508 (677)	0·352	128 (171)	7·5 (12·2)	0·154	663 (884)	0·308	166 (221)	··	··	··	··	··
**PSPRS**	9·9 (8·9)	0·278	204 (272)	0·556	52 (69)	9·2 (9·1)	0·253	246 (328)	0·505	63 (84)	··	··	··	··	··
**mPSPRS**	3·6 (3·2)	0·285	194 (259)	0·571	49 (65)	2·6 (3·0)	0·216	337 (449)	0·432	85 (113)	··	··	··	··	··
**UMSARS**	··	··	··	··	··	··	··	··	··	··	9·5 (8·2)	0·288	190 (253)	0·576	48 (64)
**UPDRS I**	0·6 (3·7)	0·044	>1500	0·088	>1500	1·6 (3·5)	0·116	1168 (1557)	0·233	290 (387)	0·04 (4·1)	0·002	>1500	0·004	>1500
**CBI-R**	8·8 (20·1)	0·109	1322 (1763)	0·218	331 (441)	12·9 (18·4)	0·175	514 (685)	0·351	128 (171)	12·0 (22·4)	0·134	875 (1167)	0·268	220 (293)
**MoCA**	-0·9 (3·5)	0·066	>1500	0·132	902 (1203)	-1·5 (3·5)	0·106	1398 (1864)	0·212	350 (467)	0·2 (3·1)	0·018	>1500	0·035	>1500
**ACE III**	-1·7 (5·3)	0·079	>1500	0·158	630 (840)	-7·1 (10·2)	0·175	514 (685)	0·349	130 (173)	-1·3 (6·9)	0·047	>1500	0·095	>1500
**ECAS**	-3·1 (12·9)	0·061	>1500	0·121	1073 (1431)	-3·3 (10·8)	0·077	>1500	0·154	663 (884)	-4·8 (11·2)	0·107	>1500	0·215	341 (455)
**FTD-FRS**	2·7 (8·7)	0·076	>1500	0·152	680 (907)	2·1 (10·0)	0·052	>1500	0·105	1425 (1900)	1·1 (8·2)	0·034	>1500	0·069	>1500

a. Sample sizes are calculated per group, based on a significance level of 5% and a power of 80%; an approximation adjusting for a drop-out rate of 25% using the formula (e.g., 233/0.75=311) is presented in parentheses.
Abbreviations: 4RT = 4-repeat tau, AD = Alzheimer disease, ACE III = Addenbrookes Cognitive Examination III, CBD-FS = Corticobasal Degeneration Functional Scale, CBI-R = Cambridge Behavioural Inventory Revised, CBS = corticobasal syndrome , ECAS = Edinburgh Cognitive and Behavioural Screen, FTD-FRS = Frontotemporal Dementia Rating Scale, IDT = Indeterminate, MoCA = Montreal Cognitive Assessment, mPSPRS = modified Progressive Supranuclear Palsy Rating Scale, MSA = multiple system atrophy, MSA QoL = MSA Quality of life scale, PSP = progressive supranuclear palsy, PSP QoL = PSP Quality of life scale, PSPRS = PSP rating scale, SD = standard deviation, SEADL = Schwab and England Activities of Daily Living Scale, UPDRS = Unified Parkinson's Disease Rating Scale, UMSARS = Unified MSA Rating Scale

Supplementary Table 10: Sample sizes required for a 2-arm, 1-year follow-up therapeutic trial to detect 25% and 50% change by Intention to Treat

	PSP					CBS					MSA				
		25% change		50% change			25% change		50% change			25% change		50% change	
	**Percent difference mean (SD)**	**Effect size**	**Sample size[a]**	**Effect size**	**Sample size[a]**	**Difference mean (SD)**	**Effect size**	**Sample size[a]**	**Effect size**	**Sample size[a]**	**Difference mean (SD)**	**Effect size**	**Sample size[a]**	**Effect size**	**Sample size[a]**
**Temporal lobe**	-2·8 (2·3)	0·269	218 (291)	0·538	55 (73)	-1·8 (4·0)	0·167	564 (752)	0·334	142 (189)	-3·3 (2·8)	0·256	240 (320)	0·512	61 (81)
**Cingulate**	-2·0 (2·1)	0·259	235 (313)	0·518	59 (79)	-2·4 (2·9)	0·265	224 (299)	0·530	57 (76)	-2·8 (2·8)	0·324	151 (201)	0·647	38 (51)
**Insula**	-1·3 (2·5)	0·156	646 (861)	0·311	163 (217)	-1·5 (3·2)	0·164	585 (780)	0·328	147 (196)	-2·2 (3·4)	0·214	344 (459)	0·427	87 (116)
**Occipital lobe**	-2·7 (2·8)	0·208	364 (485)	0·417	91 (121)	-1·1 (4·4)	0·079	>1500	0·159	622 (829)	-3·0 (3·3)	0·177	502 (669)	0·354	126 (168)
**Frontal lobe**	-3·8 (3·0)	0·341	136 (181)	0·681	35 (47)	-3·0 (4·4)	0·238	278 (371)	0·477	70 (93)	-4·2 (4·1)	0·345	133 (177)	0·690	34 (45)
**Parietal lobe**	-3·0 (2·9)	0·291	186 (248)	0·583	47 (63)	-2·6 (5·9)	0·171	538 (717)	0·343	134 (179)	-3·9 (3·6)	0·334	142 (189)	0·667	36 (48)
**Central structures**	-1·9 (3·4)	0·121	1073 (1431)	0·243	267 (356)	-3·1 (2·2)	0·393	103 (137)	0·785	26 (35)	-3·3 (3·0)	0·425	88 (117)	0·849	23 (31)
**Ventricles**	10·7 (6·7)	0·422	89 (119)	0·843	23 (31)	13·3 (9·2)	0·388	105 (140)	0·776	27 (36)	15·5 (13·0)	0·244	265 (353)	0·487	67 (89)
**Cerebellum**	-3·7 (4·3)	0·236	283 (377)	0·473	71 (95)	-2·1 (3·7)	0·199	397 (529)	0·398	100 (133)	-5·9 (4·0)	0·447	80 (107)	0·893	21 (28)
**Medulla**	1·0 (7·0)	0·028	>1500	0·055	>1500	0·3 (2·7)	0·075	>1500	0·150	699 (932)	-1·0 (3·0)	0·070	>1500	0·140	802 (1069)
**Pons**	-2·2 (3·0)	0·234	288 (384)	0·468	73 (97)	-2·4 (4·7)	0·187	450 (600)	0·374	113 (151)	-6·7 (4·7)	0·339	138 (184)	0·678	35 (47)
**Midbrain**	-0·5 (4·8)	0·029	>1500	0·057	>1500	-1·2 (2·9)	0·119	1109 (1479)	0·238	278 (371)	-2·6 (3·7)	0·279	203 (271)	0·558	51 (68)
**Midbrain:Pons ratio**	1·0 (7·0)	0·111	1275 (1700)	0·222	319 (425)	0·3 (2·7)	0·160	614 (819)	0·320	154 (205)	-1·0 (3·0)	0·257	239 (319)	0·514	60 (80)

a. Sample sizes are calculated per group, based on a significance level of 5% and a power of 80%; an approximation adjusting for a drop-out rate of 25% using the formula (e.g. 233/0.75=311) is presented in parentheses.
Abbreviations: 4RT = 4-repeat tau, AD = Alzheimer disease, CBS = corticobasal syndrome, IDT = Indeterminate, MSA = multiple system atrophy, PSP = progressive supranuclear palsy

<u>Supplementary Table 11:</u> Sample sizes required for a 2-arm, 1-year follow-up therapeutic trial to detect 20%, 25%, 30%, 40% and 50% change by Final diagnosis in PSP phenotypes

PSP		20% change		25% change		30% change		40% change		50% change	
	Difference mean (SD)	Effect size	Sample size	Effect size	Sample size	Effect size	Sample size	Effect size	Sample size	Effect size	Sample size
**RS**											
CBD-FS	12·6 (12·1)	0·209	360	0·262	230	0·314	160	0·419	90	0·523	58
SEADL	-12·7 (22·0)	0·116	1168	0·145	748	0·174	519	0·232	293	0·290	188
Milestones	0·9 (1·4)	0·131	916	0·163	592	0·196	410	0·261	231	0·326	149
PSP-QoL	19·0 (21·6)	0·176	508	0·220	325	0·265	224	0·353	127	0·441	82
UPDRS II	6·7 (6·5)	0·209	360	0·261	231	0·313	161	0·417	91	0·521	59
UPDRS III	8·6 (14·3)	0·120	1091	0·150	699	0·180	485	0·240	273	0·300	175
PSPRS	10·7 (9·3)	0·229	300	0·286	193	0·343	134	0·458	76	0·572	49
mPSPRS	3·8 (3·3)	0·228	303	0·286	193	0·343	134	0·457	76	0·571	49
UPDRS I	0·3 (4·1)	0·013	92887	0·016	61320	0·019	43485	0·026	23222	0·032	15331
CBI-R	14·9 (21·9)	0·136	850	0·170	544	0·204	378	0·272	213	0·340	137
MoCA	-0·8 (3·0)	0·055	5190	0·069	3298	0·082	2336	0·110	1298	0·137	837
ACE III	-1·8 (5·1)	0·071	3115	0·089	1983	0·106	1398	0·142	779	0·177	502
ECAS	-6·7 (11·3)	0·119	1109	0·149	708	0·179	491	0·239	276	0·299	177
FTD-FRS	4·8 (8·7)	0·111	1275	0·139	813	0·166	571	0·222	319	0·277	206
**Cortical**											
CBD-FS	14·1 (13·2)	0·213	347	0·266	223	0·319	155	0·426	87	0·532	56
SEADL	-12·2 (11·4)	0·214	344	0·268	220	0·321	153	0·428	87	0·535	56
Milestones	1·6 (1·8)	0·174	519	0·218	331	0·262	230	0·349	130	0·436	84
PSP-QoL	15·3 (23·9)	0·128	959	0·160	614	0·192	427	0·255	242	0·319	155
UPDRS II	6·9 (6·5)	0·212	350	0·265	224	0·318	156	0·424	88	0·531	57
UPDRS III	13·9 (15·0)	0·185	460	0·231	295	0·277	206	0·370	116	0·462	75
PSPRS	14·8 (11·2)	0·264	226	0·330	145	0·396	101	0·529	57	0·661	37
mPSPRS	4·3 (4·0)	0·215	341	0·269	218	0·322	152	0·430	86	0·537	55
UPDRS I	2·0 (4·3)	0·092	1856	0·115	1188	0·138	825	0·183	470	0·229	300
CBI-R	12·1 (20·4)	0·119	1109	0·148	718	0·178	496	0·237	280	0·296	180
MoCA	-3·5 (4·2)	0·169	551	0·211	354	0·253	246	0·338	138	0·422	89
ACE III	-7·1 (14·6)	0·098	1635	0·122	1056	0·146	737	0·195	414	0·244	265
ECAS	-3·9 (10·2)	0·077	2649	0·096	1704	0·115	1188	0·154	663	0·192	427
FTD-FRS	0·9 (8·7)	0·021	35597	0·027	21534	0·032	15331	0·043	8491	0·054	5384
**Subcortical**											
CBD-FS	7·3 (7·3)	0·202	386	0·252	248	0·302	173	0·403	98	0·504	63
SEADL	-12·5 (17·4)	0·144	758	0·180	485	0·216	337	0·288	190	0·360	122
Milestones	1·1 (1·2)	0·185	460	0·231	295	0·277	206	0·369	116	0·461	75
PSP-QoL	9·0 (17·7)	0·102	1510	0·127	974	0·153	672	0·204	378	0·255	242
UPDRS II	2·4 (5·4)	0·088	2028	0·110	1298	0·132	902	0·176	508	0·220	325
UPDRS III	9·0 (13·5)	0·133	888	0·166	571	0·199	397	0·265	224	0·331	144
PSPRS	6·9 (8·1)	0·171	538	0·214	344	0·257	239	0·342	135	0·428	87
mPSPRS	2·6 (2·6)	0·203	382	0·253	246	0·304	171	0·405	97	0·507	62
UPDRS I	0·7 (2·6)	0·051	6036	0·064	3833	0·077	2649	0·102	1510	0·128	959
CBI-R	-1·0 (15·5)	0·013	92887	0·016	61320	0·019	43485	0·026	23222	0·032	15331
MoCA	-0·6 (4·2)	0·029	18667	0·036	12113	0·044	8109	0·058	4667	0·073	2947
ACE III	-1·1 (4·6)	0·048	6814	0·061	4220	0·073	2947	0·097	1669	0·121	1073
ECAS	2·8 (14·3)	0·040	9812	0·049	6539	0·059	4511	0·079	2516	0·099	1603
FTD-FRS	-1·6 (7·8)	0·041	9339	0·052	5806	0·062	4085	0·083	2280	0·104	1452
Variables with fewer than 5 responses per group have been removed. Abbreviations: ACE III = Addenbrookes Cognitive Examination III, CBD-FS = Corticobasal Degeneration Functional Scale, CBI-R = Cambridge Behavioural Inventory Revised, CBS = corticobasal syndrome , ECAS = Edinburgh Cognitive and Behavioural Screen, FTD-FRS = Frontotemporal Dementia Rating Scale, IDT = Indeterminate, MoCA = Montreal Cognitive Assessment, mPSPRS = modified Progressive Supranuclear Palsy Rating Scale, PSP = progressive supranuclear palsy, PSP QoL = PSP Quality of life scale, PSPRS = PSP rating scale, SD = standard deviation, SEADL = Schwab and England Activities of Daily Living Scale, UPDRS = Unified Parkinson's Disease Rating Scale											

<u>Supplementary Table 12:</u> Sample sizes required for a 2-arm, 1-year follow-up therapeutic trial to detect 20%, 25%, 30%, 40% and 50% change by Final diagnosis in CBS phenotypes

CBS		20% change		25% change		30% change		40% change		50% change	
	Difference mean (SD)	Effect size	Sample size	Effect size	Sample size	Effect size	Sample size	Effect size	Sample size	Effect size	Sample size
**4RT**											
SEADL	-14·7 (10·3)	0·284	196	0·355	126	0·426	87	0·568	50	0·709	32
Milestones	0·9 (1·6)	0·114	1209	0·143	769	0·172	532	0·229	300	0·286	193
PSP-QoL	27·9 (23·9)	0·233	290	0·292	185	0·350	129	0·467	73	0·584	47
UPDRS II	10·3 (7·4)	0·279	203	0·349	130	0·418	91	0·558	51	0·697	33
UPDRS III	10·0 (10·3)	0·194	418	0·242	269	0·291	186	0·388	105	0·484	68
PSPRS	7·1 (9·7)	0·146	737	0·183	470	0·219	328	0·292	185	0·366	118
mPSPRS	3·2 (2·5)	0·253	246	0·316	158	0·379	110	0·506	62	0·632	40
UPDRS I	2·8 (3·4)	0·165	578	0·206	371	0·248	256	0·330	145	0·413	93
CBI-R	16·2 (14·7)	0·221	322	0·276	207	0·331	144	0·442	81	0·552	52
MoCA	-1·6 (2·7)	0·117	1148	0·146	737	0·176	508	0·234	288	0·293	184
ACE III	-6·8 (8·7)	0·156	646	0·195	414	0·234	288	0·312	162	0·390	104
ECAS	2·3 (10·4)	0·045	7753	0·056	5007	0·068	3396	0·090	1939	0·113	1230
FTD-FRS	-1·5 (15·8)	0·020	39245	0·024	27254	0·029	18667	0·039	10322	0·049	6539
**AD**											
SEADL	-11·7 (12·8)	0·182	475	0·227	306	0·272	213	0·363	120	0·454	77
Milestones	0·4 (0·5)	0·149	708	0·186	455	0·223	317	0·298	178	0·372	114
PSP-QoL	8·0 (14·4)	0·111	1275	0·139	813	0·167	564	0·222	319	0·278	204
UPDRS II	5·1 (4·8)	0·209	360	0·261	231	0·314	160	0·418	91	0·523	58
UPDRS III	6·1 (7·6)	0·160	614	0·200	393	0·240	273	0·321	153	0·401	99
PSPRS	7·6 (5·4)	0·281	200	0·351	128	0·422	89	0·562	51	0·703	33
mPSPRS	2·1 (2·4)	0·173	525	0·216	337	0·260	233	0·346	132	0·433	85
UPDRS I	1·5 (3·0)	0·098	1635	0·122	1056	0·147	727	0·196	410	0·245	262
CBI-R	6·6 (12·0)	0·110	1298	0·138	825	0·165	578	0·221	322	0·276	207
MoCA	-2·3 (3·1)	0·151	689	0·189	440	0·227	306	0·302	173	0·378	111
ACE III	-11·8 (10·0)	0·235	285	0·293	184	0·352	128	0·469	72	0·586	47
ECAS	-4·5 (10·7)	0·084	2226	0·105	1425	0·126	990	0·167	564	0·209	360
FTD-FRS	5·9 (6·4)	0·185	460	0·231	295	0·277	206	0·370	116	0·462	75
**IDT**											
SEADL	-12·2 (13·9)	0·176	508	0·220	325	0·264	226	0·353	127	0·441	82
Milestones	0·4 (0·9)	0·086	2123	0·108	1347	0·130	930	0·173	525	0·216	337
PSP-QoL	11·5 (19·0)	0·121	1073	0·151	689	0·181	480	0·242	269	0·302	173
UPDRS II	7·1 (6·5)	0·218	331	0·273	212	0·328	147	0·437	83	0·546	54
UPDRS III	5·0 (8·7)	0·114	1209	0·142	779	0·171	538	0·227	306	0·284	196
PSPRS	9·4 (8·5)	0·221	322	0·277	206	0·332	143	0·443	81	0·554	52
mPSPRS	2·7 (3·1)	0·174	519	0·218	331	0·261	231	0·348	131	0·436	84
UPDRS I	0·5 (2·6)	0·038	10872	0·047	7107	0·057	4833	0·075	2792	0·094	1778
CBI-R	16·4 (26·3)	0·125	1006	0·156	646	0·187	450	0·250	252	0·312	162
MoCA	-0·8 (3·6)	0·047	7107	0·059	4511	0·071	3115	0·095	1740	0·119	1109
ACE III	-4·3 (6·5)	0·132	902	0·165	578	0·198	401	0·265	224	0·331	144
ECAS	-7·3 (10·8)	0·135	862	0·169	551	0·203	382	0·271	215	0·338	138
FTD-FRS	3·0 (9·5)	0·064	3833	0·080	2454	0·095	1740	0·127	974	0·159	622

Variables with fewer than 5 responses per group have been removed. Abbreviations: 4RT = 4-repeat tau, AD = Alzheimer disease, ACE III = Addenbrookes Cognitive Examination III, CBD-FS = Corticobasal Degeneration Functional Scale, CBI-R = Cambridge Behavioural Inventory Revised, CBS = corticobasal syndrome , ECAS = Edinburgh Cognitive and Behavioural Screen, FTD-FRS = Frontotemporal Dementia Rating Scale, IDT = Indeterminate, MoCA = Montreal Cognitive Assessment, mPSPRS = modified Progressive Supranuclear Palsy Rating Scale, MSA = multiple system atrophy, MSA QoL = MSA Quality of life scale, PSP = progressive supranuclear palsy, PSP QoL = PSP Quality of life scale, PSPRS = PSP rating scale, SD = standard deviation, SEADL = Schwab and England Activities of Daily Living Scale, UPDRS = Unified Parkinson's Disease Rating Scale, UMSARS = Unified MSA Rating Scale

Supplementary Table 13: Sample sizes required for a 2-arm, 1-year follow-up therapeutic trial to detect 20%, 25%, 30%, 40% and 50% change by Final diagnosis in MSA phenotypes

MSA		20% change		25% change		30% change		40% change		50% change	
	Difference mean (SD)	Effect size	Sample size	Effect size	Sample size	Effect size	Sample size	Effect size	Sample size	Effect size	Sample size
**MSA-C**											
SEADL	-10·4 (19·0)	0·109	1322	0·136	850	0·164	585	0·218	331	0·273	212
Milestones	0·6 (0·9)	0·137	837	0·171	538	0·205	374	0·274	210	0·342	135
MSA-QoL	9·8 (13·8)	0·141	791	0·177	502	0·212	350	0·282	198	0·353	127
UPDRS II	5·6 (9·1)	0·123	1039	0·153	672	0·184	465	0·245	262	0·307	168
UMSARS	7·0 (7·4)	0·190	436	0·237	280	0·284	196	0·379	110	0·474	71
UPDRS I	1·2 (4·3)	0·056	5007	0·069	3298	0·083	2280	0·111	1275	0·139	813
CBI-R	5·6 (21·0)	0·053	5589	0·066	3605	0·080	2454	0·106	1398	0·133	888
MoCA	0·7 (3·7)	0·039	10322	0·049	6539	0·059	4511	0·079	2516	0·098	1635
ACE III	-2·2 (6·6)	0·067	3498	0·083	2280	0·100	1571	0·133	888	0·166	571
ECAS	-6·2 (12·7)	0·098	1635	0·123	1039	0·148	718	0·197	405	0·246	260
FTD-FRS	1·0 (9·2)	0·021	35597	0·026	23222	0·031	16336	0·042	8900	0·052	5806
**MSA-P**											
SEADL	-9·0 (14·3)	0·125	1006	0·157	638	0·188	445	0·251	250	0·313	161
Milestones	0·9 (1·2)	0·142	779	0·178	496	0·213	347	0·284	196	0·355	126
MSA-QoL	-1·9 (13·2)	0·028	20024	0·035	12815	0·042	8900	0·057	4833	0·071	3115
UPDRS II	5·0 (5·6)	0·179	491	0·224	314	0·269	218	0·358	123	0·448	79
UMSARS	11·2 (8·6)	0·261	231	0·326	149	0·392	103	0·522	59	0·653	38
UPDRS I	0·6 (4·2)	0·030	17443	0·037	11468	0·044	8109	0·059	4511	0·074	2868
CBI-R	15·7 (19·3)	0·163	592	0·203	382	0·244	265	0·325	150	0·407	96
MoCA	-0·1 (2·4)	0·008	245278	0·010	156978	0·012	109013	0·015	69769	0·019	43485
ACE III	-0·7 (6·7)	0·022	32434	0·028	20024	0·033	14416	0·044	8109	0·055	5190
ECAS	-2·5 (9·6)	0·053	5589	0·066	3605	0·079	2516	0·106	1398	0·132	902
FTD-FRS	2·0 (4·4)	0·090	1939	0·112	1252	0·135	862	0·179	491	0·224	314

Variables with fewer than 5 responses per group have been removed. Abbreviations: ACE III = Addenbrookes Cognitive Examination III, CBI-R = Cambridge Behavioural Inventory Revised, ECAS = Edinburgh Cognitive and Behavioural Screen, FTD-FRS = Frontotemporal Dementia Rating Scale, MoCA = Montreal Cognitive Assessment, MSA = multiple system atrophy, MSA-C = MSA cerebellar variant, MSA-P = MSA parkinsonism variant, MSA QoL = MSA Quality of life scale, SD = standard deviation, SEADL = Schwab and England Activities of Daily Living Scale, UPDRS = Unified Parkinson's Disease Rating Scale, UMSARS = Unified MSA Rating Scale

<u>Supplementary Table 14</u>: Sample sizes required for a 2-arm, 1-year follow-up therapeutic trial to detect 20%, 25%, 30%, 40% and 50% change by Final diagnosis according to disease group

Group		20% change		25% change		30% change		40% change		50% change	
	**Percent difference mean (SD)**	**Effect size**	**Sample size**	**Effect size**	**Sample size**	**Effect size**	**Sample size**	**Effect size**	**Sample size**	**Effect size**	**Sample size**
**PSP**											
Temporal	-2·8 (2·3)	0·208	364	0·260	233	0·312	162	0·416	92	0·520	59
Cingulate	-2·0 (2·1)	0·205	374	0·256	240	0·308	166	0·410	94	0·513	61
Insula	-1·2 (2·5)	0·117	1148	0·146	737	0·175	514	0·234	288	0·292	185
Occipital	-2·5 (2·6)	0·163	592	0·204	378	0·245	262	0·326	149	0·408	95
Frontal	-3·8 (3·1)	0·267	221	0·334	142	0·401	99	0·534	56	0·668	36
Parietal	-3·0 (2·9)	0·227	306	0·284	196	0·341	136	0·454	77	0·568	50
Central	-1·9 (3·5)	0·098	1635	0·123	1039	0·147	727	0·196	410	0·245	262
Ventricles	11·0 (6·7)	0·341	136	0·426	87	0·511	61	0·682	35	0·852	23
Cerebellum	-3·4 (4·1)	0·184	465	0·231	295	0·277	206	0·369	116	0·461	75
Medulla	0·9 (7·1)	0·020	39245	0·025	25117	0·030	17443	0·040	9812	0·050	6280
Pons	-2·2 (3·1)	0·186	455	0·232	293	0·278	204	0·371	115	0·464	74
Midbrain	-0·5 (4·9)	0·023	29675	0·029	18667	0·035	12815	0·047	7107	0·059	4511
Midbrain:Pons	0·9 (7·1)	0·088	2028	0·110	1298	0·132	902	0·176	508	0·220	325
**CBS**											
Temporal	-1·9 (4·4)	0·134	875	0·168	557	0·202	386	0·269	218	0·336	140
Cingulate	-1·9 (2·9)	0·164	585	0·205	374	0·245	262	0·327	148	0·409	95
Insula	-1·0 (3·2)	0·086	2123	0·107	1372	0·128	959	0·171	538	0·214	344
Occipital	-1·7 (4·7)	0·090	1939	0·113	1230	0·135	862	0·180	485	0·225	311
Frontal	-2·1 (4·4)	0·129	944	0·161	607	0·193	422	0·258	237	0·322	152
Parietal	-2·3 (6·3)	0·114	1209	0·143	769	0·171	538	0·229	300	0·286	193
Central	-2·8 (2·2)	0·273	212	0·342	135	0·410	94	0·547	53	0·684	35
Ventricles	13·4 (10·1)	0·244	265	0·304	171	0·365	119	0·487	67	0·609	43
Cerebellum	-1·9 (3·7)	0·127	974	0·159	622	0·191	431	0·255	242	0·319	155
Medulla	-0·3 (2·5)	0·003	1744192	0·004	981109	0·004	981109	0·006	436049	0·007	320363
Pons	-0·2 (1·1)	0·023	29675	0·029	18667	0·035	12815	0·047	7107	0·059	4511
Midbrain	-0·2 (2·1)	0·006	436049	0·007	320363	0·008	245278	0·011	129734	0·014	80091
Midbrain:Pons	-0·3 (2·5)	0·012	109013	0·015	69769	0·018	48451	0·024	27254	0·030	17443
**MSA**											
Temporal	-3·2 (2·6)	0·202	387	0·252	248	0·303	172	0·403	97	0·504	63
Cingulate	-3·0 (2·7)	0·277	206	0·346	132	0·416	92	0·554	52	0·693	34
Insula	-2·3 (3·2)	0·181	480	0·226	308	0·271	215	0·361	121	0·451	78
Occipital	-2·6 (3·3)	0·123	1039	0·153	672	0·184	465	0·245	262	0·307	168
Frontal	-4·4 (3·9)	0·298	178	0·372	114	0·446	80	0·595	45	0·744	29
Parietal	-3·9 (3·4)	0·268	220	0·336	140	0·403	98	0·537	55	0·671	36
Central	-3·6 (3·0)	0·377	111	0·471	72	0·565	50	0·753	29	0·942	19
Ventricles	15·9 (12·3)	0·219	328	0·274	210	0·329	146	0·439	82	0·548	53
Cerebellum	-5·6 (3·9)	0·332	143	0·415	92	0·497	65	0·663	37	0·829	24
Medulla	-0·6 (3·0)	0·027	21534	0·034	13580	0·040	9812	0·054	5384	0·067	3498
Pons	-7·1 (4·5)	0·310	164	0·387	106	0·464	74	0·619	42	0·774	27
Midbrain	-2·8 (3·6)	0·237	280	0·296	180	0·355	126	0·473	71	0·591	46
Midbrain:Pons	-0·6 (3·0)	0·234	288	0·293	184	0·352	128	0·469	72	0·586	47
**IDT**											
Temporal	-3·1 (1·6)	0·602	44	0·752	29	0·902	20	1·203	12	1·504	8
Cingulate	-2·6 (2·0)	0·447	80	0·558	51	0·670	36	0·893	21	1·116	14
Insula	-1·9 (1·9)	0·236	283	0·295	181	0·354	126	0·473	71	0·591	46
Occipital	-3·0 (3·8)	0·169	551	0·211	354	0·254	244	0·338	138	0·423	89
Frontal	-3·7 (1·7)	0·684	35	0·855	22	1·026	16	1·368	9	1·710	6
Parietal	-2·6 (2·0)	0·222	319	0·278	204	0·333	143	0·444	81	0·556	52
Central	-1·0 (1·2)	0·114	1209	0·142	779	0·170	544	0·227	306	0·284	196
Ventricles	9·2 (6·7)	0·217	334	0·271	215	0·325	150	0·434	84	0·542	54
Cerebellum	-4·5 (4·0)	0·247	258	0·309	165	0·370	116	0·494	65	0·617	42
Medulla	0·8 (1·8)	0·121	1073	0·152	680	0·182	475	0·243	267	0·303	172
Pons	-0·3 (1·4)	0·033	14416	0·041	9339	0·049	6539	0·065	3716	0·082	2336
Midbrain	0·5 (4·5)	0·038	10872	0·048	6814	0·058	4667	0·077	2649	0·096	1704
Midbrain:Pons	0·8 (1·8)	0·067	3498	0·083	2280	0·100	1571	0·133	888	0·166	571

Abbreviations: CBS = corticobasal syndrome, IDT = Indeterminate, MSA = multiple system atrophy, PSP = progressive supranuclear palsy, SD = standard deviation.

Supplementary Table 15: Sample sizes required for a 2-arm, 1-year follow-up therapeutic trial to detect 20%, 25%, 30%, 40% and 50% change by Intention to Treat in PSP phenotypes

PSP		20% change		25% change		30% change		40% change		50% change	
	Difference mean (SD)	Effect size	Sample size	Effect size	Sampl e size	Effect size	Sampl e size	Effect size	Sampl e size	Effect size	Sampl e size
**RS**											
CBD-FS	11·8 (12·1)	0·196	410	0·246	260	0·295	181	0·393	103	0·491	66
SEADL	-15·9 (19·4)	0·164	585	0·206	371	0·247	258	0·329	146	0·411	94
MS	1·0 (1·5)	0·132	902	0·165	578	0·198	401	0·265	224	0·331	144
PSP-QoL	18·4 (22·3)	0·165	578	0·206	371	0·248	256	0·33	145	0·413	93
UPDRS II	7·1 (6·1)	0·231	295	0·288	190	0·346	132	0·461	75	0·577	48
UPDRS III	9·6 (13·7)	0·139	813	0·174	519	0·209	360	0·279	203	0·349	130
PSPRS	11·6 (9·1)	0·255	242	0·319	155	0·383	108	0·51	61	0·638	40
mPSPRS	4·0 (3·3)	0·240	273	0·300	175	0·360	122	0·480	69	0·600	45
UPDRS I	0·4 (4·0)	0·019	43485	0·024	27254	0·028	20024	0·038	10872	0·047	7107
CBI-R	15·2 (21·8)	0·139	813	0·174	519	0·209	360	0·278	204	0·348	131
MoCA	-0·6 (3·0)	-0·037	11468	-0·047	7107	-0·056	5007	-0·075	2792	-0·093	1816
ACE III	-1·4 (5·3)	-0·054	5384	-0·068	3396	-0·081	2394	-0·109	1322	-0·136	850
ECAS	-4·5 (10·7)	-0·084	2226	-0·105	1425	-0·126	990	-0·167	564	-0·209	360
FTD-FRS	4·3 (8·5)	0·1	1571	0·125	1006	0·15	699	0·2	393	0·251	250
**Cortical**											
CBD-FS	15·8 (8·5)	0·374	113	0·467	73	0·56	51	0·747	29	0·934	19
SEADL	-14·5 (7·5)	0·386	106	0·482	69	0·578	48	0·771	27	0·964	18
MS	0·9 (0·9)	0·205	374	0·257	239	0·308	166	0·411	94	0·513	61
PSP-QoL	16·7 (19·8)	0·168	557	0·211	354	0·253	246	0·337	139	0·421	90
UPDRS II	11·6 (6·6)	0·355	126	0·443	81	0·532	56	0·709	32	0·887	21
UPDRS III	13·3 (10·3)	0·259	235	0·323	151	0·388	105	0·517	60	0·646	39
PSPRS	13·9 (8·0)	0·348	131	0·435	84	0·522	59	0·696	33	0·871	22
mPSPRS	5·8 (2·9)	0·403	98	0·504	63	0·605	44	0·806	25	1·008	16
UPDRS I	3·6 (3·3)	0·221	322	0·276	207	0·331	144	0·441	82	0·552	52
CBI-R	7·3 (12·9)	0·113	1230	0·141	791	0·169	551	0·225	311	0·282	198
MoCA	-3·7 (2·3)	0·327	148	0·409	95	0·491	66	0·655	38	0·819	24
ACE III	-5·2 (6·9)	0·151	689	0·189	440	0·226	308	0·302	173	0·377	111
ECAS	-12·9 (13·6)	0·19	436	0·237	280	0·285	194	0·379	110	0·474	71
FTD-FRS	4·6 (9·1)	0·101	1540	0·126	990	0·151	689	0·202	386	0·252	248
**Subcortical**											
CBD-FS	8·4 (6·2)	0·272	213	0·34	137	0·408	95	0·544	54	0·681	35
SEADL	-5·2 (25·7)	0·041	9339	0·051	6036	0·061	4220	0·081	2394	0·101	1540
MS	0·9 (1·2)	0·156	646	0·195	414	0·234	288	0·312	162	0·39	104
PSP-QoL	9·2 (17·1)	0·108	1347	0·135	862	0·162	599	0·215	341	0·269	218
UPDRS II	1·9 (5·7)	0·067	3498	0·083	2280	0·1	1571	0·133	888	0·167	564
UPDRS III	7·3 (13·2)	0·111	1275	0·139	813	0·167	564	0·223	317	0·279	203
PSPRS	5·9 (8·0)	0·149	708	0·187	450	0·224	314	0·299	177	0·373	114
mPSPRS	2·3 (2·6)	0·176	508	0·219	328	0·263	228	0·351	128	0·439	82
UPDRS I	0·2 (2·9)	0·011	129734	0·014	80091	0·016	61320	0·022	32434	0·027	21534
CBI-R	-1·1 (15·3)	0·015	69769	0·018	48451	0·022	32434	0·03	17443	0·037	11468
MoCA	-0·6 (4·3)	0·027	21534	0·034	13580	0·041	9339	0·054	5384	0·068	3396
ACE III	-1·1 (4·7)	0·046	7420	0·057	4833	0·069	3298	0·092	1856	0·115	1188
ECAS	1·4 (15·2)	0·018	48451	0·023	29675	0·027	21534	0·036	12113	0·045	7753
FTD-FRS	-1·7 (8·1)	0·041	9339	0·052	5806	0·062	4085	0·083	2280	0·104	1452

Variables with fewer than 5 responses per group have been removed. Abbreviations: ACE III = Addenbrookes Cognitive Examination III, CBD-FS = Corticobasal Degeneration Functional Scale, CBI-R = Cambridge Behavioural Inventory Revised, CBS = corticobasal syndrome , ECAS = Edinburgh Cognitive and Behavioural Screen, FTD-FRS = Frontotemporal Dementia Rating Scale, IDT = Indeterminate, MoCA = Montreal Cognitive Assessment, mPSPRS = modified Progressive Supranuclear Palsy Rating Scale, PSP = progressive supranuclear palsy, PSP QoL = PSP Quality of life scale, PSPRS = PSP rating scale, SD = standard deviation, SEADL = Schwab and England Activities of Daily Living Scale, UPDRS = Unified Parkinson's Disease Rating Scale

Supplementary Table 16: Sample sizes required for a 2-arm, 1-year follow-up therapeutic trial to detect 20%, 25%, 30%, 40% and 50% change by Intention to Treat in CBS phenotypes

CBS		20% change		25% change		30% change		40% change		50% change	
	Difference mean (SD)	Effect size	Sample size	Effect size	Sample size	Effect size	Sample size	Effect size	Sample size	Effect size	Sample size
**4RT**											
SEADL	-14·7 (10·3)	0·284	196	0·355	126	0·426	87	0·568	50	0·709	32
Milestones	0·9 (1·6)	0·114	1209	0·143	769	0·172	532	0·229	300	0·286	193
PSP-QoL	27·9 (23·9)	0·233	290	0·292	185	0·350	129	0·467	73	0·584	47
UPDRS II	10·3 (7·4)	0·279	203	0·349	130	0·418	91	0·558	51	0·697	33
UPDRS III	10·0 (10·3)	0·194	418	0·242	269	0·291	186	0·388	105	0·484	68
PSPRS	7·1 (9·7)	0·146	737	0·183	470	0·219	328	0·292	185	0·366	118
mPSPRS	3·2 (2·5)	0·253	246	0·316	158	0·379	110	0·506	62	0·632	40
UPDRS I	2·8 (3·4)	0·165	578	0·206	371	0·248	256	0·330	145	0·413	93
CBI-R	16·2 (14·7)	0·221	322	0·276	207	0·331	144	0·442	81	0·552	52
MoCA	-1·6 (2·7)	0·117	1148	0·146	737	0·176	508	0·234	288	0·293	184
ACE III	-6·8 (8·7)	0·156	646	0·195	414	0·234	288	0·312	162	0·390	104
ECAS	2·3 (10·4)	0·045	7753	0·056	5007	0·068	3396	0·090	1939	0·113	1230
FTD-FRS	-1·5 (15·8)	0·020	39245	0·024	27254	0·029	18667	0·039	10322	0·049	6539
**AD**											
SEADL	-11·7 (12·8)	0·182	475	0·227	306	0·272	213	0·363	120	0·454	77
Milestones	0·4 (0·5)	0·149	708	0·186	455	0·223	317	0·298	178	0·372	114
PSP-QoL	8·0 (14·4)	0·111	1275	0·139	813	0·167	564	0·222	319	0·278	204
UPDRS II	5·1 (4·8)	0·209	360	0·261	231	0·314	160	0·418	91	0·523	58
UPDRS III	6·1 (7·6)	0·160	614	0·200	393	0·240	273	0·321	153	0·401	99
PSPRS	7·6 (5·4)	0·281	200	0·351	128	0·422	89	0·562	51	0·703	33
mPSPRS	2·1 (2·4)	0·173	525	0·216	337	0·260	233	0·346	132	0·433	85
UPDRS I	1·5 (3·0)	0·098	1635	0·122	1056	0·147	727	0·196	410	0·245	262
CBI-R	6·6 (12·0)	0·110	1298	0·138	825	0·165	578	0·221	322	0·276	207
MoCA	-2·3 (3·1)	0·151	689	0·189	440	0·227	306	0·302	173	0·378	111
ACE III	-11·8 (10·0)	0·235	285	0·293	184	0·352	128	0·469	72	0·586	47
ECAS	-4·5 (10·7)	0·084	2226	0·105	1425	0·126	990	0·167	564	0·209	360
FTD-FRS	5·9 (6·4)	0·185	460	0·231	295	0·277	206	0·370	116	0·462	75
**IDT**											
SEADL	-12·2 (13·9)	0·176	508	0·220	325	0·264	226	0·353	127	0·441	82
Milestones	0·4 (0·9)	0·086	2123	0·108	1347	0·130	930	0·173	525	0·216	337
PSP-QoL	11·5 (19·0)	0·121	1073	0·151	689	0·181	480	0·242	269	0·302	173
UPDRS II	7·1 (6·5)	0·218	331	0·273	212	0·328	147	0·437	83	0·546	54
UPDRS III	5·0 (8·7)	0·114	1209	0·142	779	0·171	538	0·227	306	0·284	196
PSPRS	9·4 (8·5)	0·221	322	0·277	206	0·332	143	0·443	81	0·554	52
mPSPRS	2·7 (3·1)	0·174	519	0·218	331	0·261	231	0·348	131	0·436	84
UPDRS I	0·5 (2·6)	0·038	10872	0·047	7107	0·057	4833	0·075	2792	0·094	1778
CBI-R	16·4 (26·3)	0·125	1006	0·156	646	0·187	450	0·250	252	0·312	162
MoCA	-0·8 (3·6)	0·047	7107	0·059	4511	0·071	3115	0·095	1740	0·119	1109
ACE III	-4·3 (6·5)	0·132	902	0·165	578	0·198	401	0·265	224	0·331	144
ECAS	-7·3 (10·8)	0·135	862	0·169	551	0·203	382	0·271	215	0·338	138
FTD-FRS	3·0 (9·5)	0·064	3833	0·080	2454	0·095	1740	0·127	974	0·159	622

Variables with fewer than 5 responses per group have been removed. Abbreviations: 4RT = 4-repeat tau, AD = Alzheimer disease, ACE III = Addenbrookes Cognitive Examination III, CBD-FS = Corticobasal Degeneration Functional Scale, CBI-R = Cambridge Behavioural Inventory Revised, CBS = corticobasal syndrome , ECAS = Edinburgh Cognitive and Behavioural Screen, FTD-FRS = Frontotemporal Dementia Rating Scale, IDT = Indeterminate, MoCA = Montreal Cognitive Assessment, mPSPRS = modified Progressive Supranuclear Palsy Rating Scale, MSA = multiple system atrophy, MSA QoL = MSA Quality of life scale, PSP = progressive supranuclear palsy, PSP QoL = PSP Quality of life scale, PSPRS = PSP rating scale, SD = standard deviation, SEADL = Schwab and England Activities of Daily Living Scale, UPDRS = Unified Parkinson's Disease Rating Scale, UMSARS = Unified MSA Rating Scale

<u>Supplementary Table 17</u>: Sample sizes required for a 2-arm, 1-year follow-up therapeutic trial to detect 20%, 25%, 30%, 40% and 50% change by Intention to Treat in MSA phenotypes

MSA		20% change		25% change		30% change		40% change		50% change	
	**Difference mean (SD)**	**Effect size**	**Sample size**	**Effect size**	**Sample size**	**Effect size**	**Sample size**	**Effect size**	**Sample size**	**Effect size**	**Sample size**
**MSA-C**											
SEADL	-10·4 (19·0)	0·109	1322	0·136	850	0·164	585	0·218	331	0·273	212
MS	0·6 (0·9)	0·137	837	0·171	538	0·205	374	0·274	210	0·342	135
MSA-QoL	9·8 (13·8)	0·141	791	0·177	502	0·212	350	0·282	198	0·353	127
UPDRS II	5·6 (9·1)	0·123	1039	0·153	672	0·184	465	0·245	262	0·307	168
UMSARS	7·0 (7·4)	0·19	436	0·237	280	0·284	196	0·379	110	0·474	71
UPDRS I	1·2 (4·3)	0·056	5007	0·069	3298	0·083	2280	0·111	1275	0·139	813
CBI-R	5·6 (21·0)	0·053	5589	0·066	3605	0·08	2454	0·106	1398	0·133	888
MoCA	0·7 (3·7)	0·039	10322	0·049	6539	0·059	4511	0·079	2516	0·098	1635
ACE III	-2·2 (6·6)	0·067	3498	0·083	2280	0·1	1571	0·133	888	0·166	571
ECAS	-6·2 (12·7)	0·098	1635	0·123	1039	0·148	718	0·197	405	0·246	260
FTD-FRS	1·0 (9·2)	0·021	35597	0·026	23222	0·031	16336	0·042	8900	0·052	5806
**MSA-P**											
SEADL	-7·2 (10·8)	0·133	888	0·166	571	0·199	397	0·265	224	0·332	143
MS	0·7 (1·1)	0·131	916	0·163	592	0·196	410	0·262	230	0·327	148
MSA-QoL	-1·9 (13·2)	0·028	20024	0·035	12815	0·042	8900	0·057	4833	0·071	3115
UPDRS II	3·4 (4·5)	0·151	689	0·189	440	0·227	306	0·302	173	0·378	111
UMSARS	11·2 (8·6)	0·261	231	0·326	149	0·392	103	0·522	59	0·653	38
UPDRS I	-0·8 (3·8)	0·043	8491	0·054	5384	0·064	3833	0·086	2123	0·107	1372
CBI-R	17·8 (22·5)	0·158	630	0·198	401	0·237	280	0·316	158	0·395	102
MoCA	-0·2 (2·6)	0·013	92887	0·016	61320	0·02	39245	0·026	23222	0·033	14416
ACE III	-0·8 (7·1)	0·022	32434	0·028	20024	0·033	14416	0·045	7753	0·056	5007
ECAS	-3·6 (9·4)	0·076	2719	0·095	1740	0·114	1209	0·152	680	0·19	436
FTD-FRS	1·8 (5·4)	0·066	3605	0·082	2336	0·098	1635	0·131	916	0·164	585
Variables with fewer than 5 responses per group have been removed. Abbreviations: ACE III = Addenbrookes Cognitive Examination III, CBI-R = Cambridge Behavioural Inventory Revised, ECAS = Edinburgh Cognitive and Behavioural Screen, FTD-FRS = Frontotemporal Dementia Rating Scale, MoCA = Montreal Cognitive Assessment, MSA = multiple system atrophy, MSA-C = MSA cerebellar variant, MSA-P = MSA parkinsonism variant, MSA QoL = MSA Quality of life scale, SD = standard deviation, SEADL = Schwab and England Activities of Daily Living Scale, UPDRS = Unified Parkinson's Disease Rating Scale, UMSARS = Unified MSA Rating Scale											

Supplementary Table 18: Sample sizes required for a 2-arm, 1-year follow-up therapeutic trial to detect 20%, 25%, 30%, 40% and 50% change by Intention to Treat according to disease group

IMAGING		20% change		25% change		30% change		40% change		50% change	
	**Percent difference mean (SD)**	**Effect size**	**Sample size**	**Effect size**	**Sample size**	**Effect size**	**Sample size**	**Effect size**	**Sample size**	**Effect size**	**Sample size**
**PSP**											
Temporal	-2·8 (2·3)	0·215	341	0·269	218	0·323	151	0·43	86	0·538	55
Cingulate	-2·0 (2·1)	0·207	367	0·259	235	0·311	163	0·414	93	0·518	59
Insula	-1·3 (2·5)	0·124	1022	0·156	646	0·187	450	0·249	254	0·311	163
Occipital	-2·7 (2·8)	0·167	564	0·208	364	0·250	252	0·333	143	0·417	91
Frontal	-3·8 (3·0)	0·272	213	0·341	136	0·409	95	0·545	54	0·681	35
Parietal	-3·0 (2·9)	0·233	290	0·291	186	0·350	129	0·466	73	0·583	47
Central	-1·9 (3·4)	0·097	1669	0·121	1073	0·146	737	0·194	418	0·243	267
Ventricles	10·7 (6·7)	0·337	139	0·422	89	0·506	62	0·675	35	0·843	23
Cerebellum	-3·7 (4·3)	0·189	440	0·236	283	0·284	196	0·378	111	0·473	71
Medulla	1·0 (7·0)	0·022	32434	0·028	20024	0·033	14416	0·044	8109	0·055	5190
Pons	-2·2 (3·0)	0·187	450	0·234	288	0·281	200	0·374	113	0·468	73
Midbrain	-0·5 (4·8)	0·023	29675	0·029	18667	0·034	13580	0·046	7420	0·057	4833
Midbrain:Pons	1·0 (7·0)	0·089	1983	0·111	1275	0·133	888	0·178	496	0·222	319
**CBS**											
Temporal	-1·8 (4·0)	0·134	875	0·167	564	0·2	393	0·267	221	0·334	142
Cingulate	-2·4 (2·9)	0·212	350	0·265	224	0·318	156	0·424	88	0·53	57
Insula	-1·5 (3·2)	0·131	916	0·164	585	0·197	405	0·262	230	0·328	147
Occipital	-1·1 (4·4)	0·063	3956	0·079	2516	0·095	1740	0·127	974	0·159	622
Frontal	-3·0 (4·4)	0·191	431	0·238	278	0·286	193	0·382	109	0·477	70
Parietal	-2·6 (5·9)	0·137	837	0·171	538	0·206	371	0·274	210	0·343	134
Central	-3·1 (2·2)	0·314	160	0·393	103	0·471	72	0·628	41	0·785	26
Ventricles	13·3 (9·2)	0·310	164	0·388	105	0·465	74	0·620	42	0·776	27
Cerebellum	-2·1 (3·7)	0·159	622	0·199	397	0·239	276	0·318	156	0·398	100
Medulla	0·3 (2·7)	0·060	4361	0·075	2792	0·09	1939	0·120	1091	0·15	699
Pons	-2·4 (4·7)	0·150	699	0·187	450	0·225	311	0·300	175	0·374	113
Midbrain	-1·2 (2·9)	0·095	1740	0·119	1109	0·143	769	0·190	436	0·238	278
Midbrain:Pons	0·3 (2·7)	0·128	959	0·160	614	0·192	427	0·256	240	0·32	154
**MSA**											
Temporal	-3·3 (2·8)	0·205	374	0·256	240	0·307	168	0·409	95	0·512	61
Cingulate	-2·8 (2·8)	0·259	235	0·324	151	0·388	105	0·518	59	0·647	38
Insula	-2·2 (3·4)	0·171	538	0·214	344	0·256	240	0·342	135	0·427	87
Occipital	-3·0 (3·3)	0·141	791	0·177	502	0·212	350	0·283	197	0·354	126
Frontal	-4·2 (4·1)	0·276	207	0·345	133	0·414	93	0·552	52	0·690	34
Parietal	-3·9 (3·6)	0·267	221	0·334	142	0·4	99	0·534	56	0·667	36
Central	-3·3 (3·0)	0·340	137	0·425	88	0·51	61	0·679	35	0·849	23
Ventricles	15·5 (13·0)	0·195	414	0·244	265	0·292	185	0·390	104	0·487	67
Cerebellum	-5·9 (4·0)	0·357	124	0·447	80	0·536	56	0·714	32	0·893	21
Medulla	-1·0 (3·0)	0·056	5007	0·070	3205	0·084	2226	0·112	1252	0·140	802
Pons	-6·7 (4·7)	0·271	215	0·339	138	0·407	96	0·542	54	0·678	35
Midbrain	-2·6 (3·7)	0·223	317	0·279	203	0·335	141	0·447	80	0·558	51
Midbrain:Pons	-1·0 (3·0)	0·205	374	0·257	239	0·308	166	0·411	94	0·514	60
**IDT**											
Temporal	-2·8 (1·3)	0·769	28	0·961	18	1·153	13	1·537	8	1·922	5
Cingulate	-2·9 (1·9)	0·403	98	0·504	63	0·605	44	0·806	25	1·008	16
Insula	-1·3 (1·2)	0·222	319	0·278	204	0·333	143	0·445	80	0·556	52
Occipital	-2·0 (2·1)	0·171	538	0·213	347	0·256	240	0·341	136	0·427	87
Frontal	-3·3 (1·9)	0·400	99	0·500	64	0·6	45	0·800	26	1·001	17
Parietal	-2·2 (1·7)	0·207	367	0·258	237	0·31	164	0·413	93	0·516	60
Central	-2·3 (2·8)	0·149	708	0·186	455	0·223	317	0·298	178	0·372	114
Ventricles	13·2 (9·1)	0·250	252	0·312	162	0·375	113	0·500	64	0·625	41
Cerebellum	-3·0 (1·6)	0·388	105	0·485	68	0·582	47	0·776	27	0·971	18
Medulla	0·4 (1·8)	0·059	4511	0·074	2868	0·089	1983	0·118	1128	0·148	718
Pons	-0·4 (1·5)	0·005	627910	0·006	436049	0·007	320363	0·009	193800	0·012	109013
Midbrain	0·3 (4·5)	0·031	16336	0·039	10322	0·047	7107	0·063	3956	0·078	2581
Midbrain:Pons	0·4 (1·8)	0·067	3498	0·084	2226	0·101	1540	0·134	875	0·168	557
Abbreviations: CBS = corticobasal syndrome, IDT = Indeterminate, MSA = multiple system atrophy, PSP = progressive supranuclear palsy, SD = standard deviation.											

Supplementary Table 19: **Inclusion and Exclusion Criteria for PSP Trial Involvement, applied in the current study**

**Inclusion criteria**	
**Type of participant and target disease characteristics:**	a) Probable or possible PSP as defined as:<br>i)<br>(1) A progressive history of postural instability during the first 3 years that symptoms are present,<br>OR<br>(2) A progressive history of falls during the first 3 years that symptoms are present;<br><br>ii)<br>(1) Vertical supranuclear gaze palsy defined as clear limitation of the range of voluntary gaze in the vertical more than in the horizontal plane, more than expected with age, which is overcome by activation with the vestibulo-ocular reflex,<br>OR<br>(2) Slow velocity of vertical saccades (i.e. decreased velocity (and gain) of vertical greater than horizontal saccadic eye movements);<br>iii) Age at symptom onset of 40 to 85 years by history and current age between 41 and 86 years, inclusive, at time of screening;<br>iv) An akinetic-rigid syndrome; and<br>v) Present of PSP symptoms for less than or equal to 5 years (determined by the best judgement of the investigator) at screening.
	b) Body weight range of $\geq$43 kg/95 lbs to $\leq$ 120kg/265 lbs.
	c) Able to ambulate independently or with assistance defined as the ability to take at least 10 steps with a walker or the ability to take at least 10 steps without a walker or cane with the assistance of another person who can only have contact with one upper extremity.
	d) Able to tolerate MRI.
	e) Able to perform all protocol-specific assessments and comply with the study visit schedule.
	f) Able to comply with neuropsychological evaluation at screening and throughout the 52-week double-blind period of the study.
	g) Have reliable caregiver to accompany participant to all study visits.
	h) Score $\geq$20 on the Mini Mental State Examination (MMSE) at screening.
	i) Participant must reside outside a skilled nursing facility or dementia care facility at the time of screening and admission to such a facility must not be planned.
	j) If participant is receiving coenzyme Q10, levodopa/carbidopa, levodopa/benserazide, fava bean extract, a dopamine agonist, catechol-0-methyltransferase inhibitor, amantadine, or other Parkinson's disease medications, the dose must have been stable for at least 60 days prior to screening and expected to remain stable for the double-blind period of the study.
	k) Stable on other chronic medications for at least 30 days prior to screening.
**Age and reproductive status:**	a) Males and Females, ages 41 to 86 years inclusive.
	b) Women of childbearing potential must have a negative serum or urine pregnancy test within 24 hours prior to the start of study treatment.
	c) Women must not be breastfeeding.
	d) Women of childbearing potential must agree to follow instructions for method(s) of contraception for the duration of treatment with study treatment(s).
	e) Males who are sexually active must agree to follow instructions for method(s) of contraception for the duration of treatment with study treatment(s).

Exclusion criteria	
**Medical conditions**	a) History of clinically significant haematological, endocrine, cardiovascular, renal, hepatic, gastrointestinal, psychiatric, or neurological disease.
	b) History of or screening brain MRI scan indicative of significant abnormality.
	c) Known presence of disease-associated genetic mutation.
	d) Any major surgery within 4 weeks of screening.
	e) History of deep brain stimulator surgery other than sham surgery for deep brain stimulation (DBS) clinical trial.
	f) Inability to be venepunctured and/or tolerate venous access.
	g) Contraindication to the MRI examination for any reason.
	h) History of cancer within 5 years of screening.
	i) Blood transfusion within 4 weeks of screening.
	j) Any vaccination within 30 days prior to screening.
	k) Known history of human immunodeficiency virus infection.
**Prior/Concomitant therapy**	a) Concurrent treatment with memantine; acetylcholinesterase inhibitors, antipsychotic agents, or mood stabilizers (e.g., valproate, lithium); or benzodiazepines.
	b) Receipt of systemic corticosteroids within 30 days prior to screening.
	c) Receipt of an investigational immunomodulator or monoclonal antibody within 180 days (or 5 half-lives, whichever is longer) prior to screening.
	d) Treatment with any other investigational drugs including placebo or devices within 90 days prior to screening.
**Physical and Laboratory Test Findings**	a) Evidence of organ dysfunction or any clinically significant deviation from normal in physical examination, vital signs, ECG, or clinical laboratory determinations.
	b) Clinically significant abnormality on 12-lead ECG prior to study drug administration, confirmed by repeat.
	c) Total bilirubin, alanine aminotransferase (ALT) or aspartate aminotransferase (AST) greater than 2 times the upper limit of normal (ULN), confirmed by repeat.
	d) Serum creatinine >168 µmol/L (1.9 mg/dL), confirmed by repeat.
	e) Haematocrit less than 35% for males and less than 32% for females, absolute neutrophil cell count of ≤1500/µL (with the exception of a documented history of a chronic benign neutropenia), or platelet cell count of <120,000/µL; INR >1.4 or other coagulopathy, confirmed by repeat.
	f) A clinically significant abnormal thyroid stimulating hormone (TSH) test.
	g) For those participating in the CSF substudy, an abnormally increased number of white blood cells (>7 cells/mm3) in the CSF obtained at the screening visit; if there is evidence that the spinal tap was traumatic, participants with >7 cells/mm3 must be discussed with the Medical Monitor to determine if they may be eligible.
	h) Haemoglobin A1C >7.5%, confirmed by repeat.
	i) Positive blood screen for hepatitis C antibody, hepatitis B surface antigen.
**Allergies and Adverse Drug Reaction**	a) History of allergy, hypersensitivity, or serious adverse reaction to monoclonal antibodies or related compounds.
	b) Allergy to any of the components of the study drug.
	c) History of any significant drug allergy (such as anaphylaxis or hepatotoxicity).
**Other exclusion criteria**	a) Prisoners or participants who are involuntarily incarcerated.
	b) Participants who are compulsorily detained for treatment of either a psychiatric or physical (e.g., infectious disease) illness

Supplementary Table 20: Inclusion and Exclusion Criteria for MSA Trial Involvement, applied in the current study

**Inclusion criteria**
Male and female subjects between the ages of ≥ 40 to ≤ 80 years at time of Screening.
Subjects must provide a written signed and dated informed consent form/forms (IRB/EC specific) in accordance with regulatory and institutional guidelines, prior to the initiation of any protocol required procedures. Only patients with the capacity to understand the nature, significance and scope of the clinical trial interventions and to express their wishes accordingly may provide consent to participate in the study.
Caregivers must be willing to sign and date an IRB/EC-approved written informed consent form that outlines the caregiver expectations and responsibilities in this study, in accordance with regulatory and institutional guidelines, as appropriate.<br>• Diagnosis of probable or possible MSA according to consensus clinical criteria (Gilman et al 2008), including subjects with MSA of either subtype (MSA-P or MSA-C);<br>• Able to ambulate without the assistance of another person, defined as the ability to take at least 10 steps. Use of assistive devices (e.g., walker or cane) is allowed;<br>• Anticipated survival of at least 3 years at the time of Screening, as judged by the Investigator.<br>d. A brain MRI scan (conducted within the 14 days prior to Baseline/Day 1, approximately) that does not rule out a diagnosis of MSA;<br>• Able to tolerate MRI;<br>• Body mass index (BMI) < 40 kg/m^2 at Screening;<br>• Able to swallow tablets whole and anticipated to be able to do so throughout the duration of the study;<br>• Willing and able to adhere to the study drug regimen;<br>• Willing and able to perform all protocol-specified assessments and comply with the study visit schedule;<br>• Able to read, understand, and speak local language fluently to ensure comprehension of informed consent and protocol-specified assessments;<br>• Must have reliable caregiver to accompany subject to study visits, with the same caregiver completing caregiver assessments at Baseline, Week 24 and Week 48/Early Discontinuation, when possible. Caregiver must be able to read, understand, and speak local language fluently to ensure comprehension of informed consent and protocol specified assessments. Caregiver must also have frequent contact with subject (at least 3 hours per week at one time or different times) and be willing to monitor the subject's health and concomitant medications throughout the study;<br>• If subject is receiving treatment for MSA, the doses must have been stable for at least 30 days prior to Baseline/Randomization and medications present at Baseline expected to remain relatively stable during the study period. This may include medications commonly used for Parkinson's disease (e.g., coenzyme Q10, levodopa/ carbidopa, levodopa/ benserazide, fava bean extract, a dopamine agonist, catechol-0-methyltransferase inhibitor, amantadine) or those for autonomic dysfunction (e.g., ephedrine, midodrine, fludrocortisone, octreotide, desmopressin, oxybutynine, droxidopa);<br>• Stable on other chronic medications and supplements for at least 30 days prior to Baseline/Randomization and expected to remain relatively stable during the study period;<br>• Women of child bearing potential (WOCBP) and fertile men (including those vasectomized for less than 6 months) with female partners who are WOCBP (not surgically sterile and not postmenopausal) must agree to use highly effective birth control, including two methods of contraception, for the duration of the study (i.e., beginning 30 days prior to Baseline/Randomization and extending to 30 days for women and 90 days for men after the last dose of study drug);<br>• The two methods of contraception should include:<br>• one barrier method (e.g., diaphragm with spermicide, condom with spermicidal gel, cervical cap);<br>• and one other method that could include hormonal contraceptives (e.g., oral contraceptives, injectable contraceptives, contraceptive implant, patch) used for at least 4 weeks prior to sexual intercourse;<br>• WOCBP must have a negative serum pregnancy test at Screening and a negative urine pregnancy test within approximately 24 hours prior to dosing at Baseline/ Randomization.

**Exclusion criteria**
Subjects having advanced disease, defined by the presence of one or more of the following on the UMSARS Part I:<br>• Speech impairment, as assessed by a score greater than or equal to 3, Question 1;<br>• Swallowing impairment, as assessed by a score of greater than or equal to 3 on Question 2;<br>• Falling more frequently than once per week, as assessed by a score of greater than or equal to 3 on Question 8; or Subjects having significant cognitive impairment, defined by a score of less than or equal to 20 on the MoCA (score includes any limitation due to motor impairments).
Any condition that would interfere with the subject's ability to comply with study instructions, place the subject at unacceptable risk, and/or confound the interpretation of safety or efficacy data from the study, as judged by the Investigator.
Diagnosis of neurological disorders, other than MSA, including (but not limited to) the following:<br>• Idiopathic Parkinson's disease or another form of parkinsonism (e.g., dementia with Lewy bodies, drug-induced, post-encephalitic, vascular), which has not subsequently revised to a diagnosis of MSA;<br>• Any other neurodegenerative disease (e.g., Alzheimer's disease, frontotemporal dementia, amyotrophic lateral sclerosis, prion disease);<br>• Any other clinically significant neurological disorder (e.g., history of stroke, history of head injury with loss of consciousness for at least 15 minutes within the past 20 years, history of seizure disorder, brain tumor, or other space-occupying lesion).
History of or Screening brain MRI scan indicative of significant abnormality, including (but not limited to) the following: prior hemorrhage or infarct greater than 1 cm3; 3 or more lacunar infarcts; cerebral contusion; encephalomalacia; aneurysm; vascular malformation; subdural hematoma; hydrocephalus; space-occupying lesion (e.g., abscess or brain tumor).
Contraindication to MRI examination for any reason
For those participating in the optional CSF sub-study, contraindication to undergoing an LP including, but not limited to: inability to tolerate an appropriately flexed position for the time necessary to perform an LP; infection at the desired LP site; taking antiplatelet/anticoagulant medications that cannot be discontinued for a short period of time prior to performing the LP; degenerative arthritis of the lumbar spine; suspected non-communicating hydrocephalus or intracranial mass; prior history of spinal mass or trauma.
Presence of clinically significant thyroid disease with abnormal free T4 levels and TSH >10 mIU/L (despite treatment) at Screening, confirmed by repeat.
Within 1 year prior to Screening or between Screening and Baseline (Day -1), any of the following: myocardial infarction; hospitalization for congestive heart failure; hospitalization for, or symptoms of, unstable angina; or syncope not related to MSA.
Diagnosis of clinically significant psychiatric disorder including (but not limited to) the following: any psychotic disorder, severe bipolar or unipolar depression, prior history of suicidal thoughts or behavior that are believed to represent a current safety risk.
History of substance use disorder (drug or alcohol) in the last 12 months, with the exception of nicotine, as defined by DSM-V criteria.
History or presence of gastrointestinal or other disease known to interfere with absorption, distribution, metabolism, or excretion of drugs, or a history of surgery known to interfere with absorption or excretion of drugs (i.e., gastric bypass).
History of any other clinically significant disease (e.g., autoimmune, cardiovascular, endocrine, gastrointestinal, hematological, hepatic, immunological, infectious, neurological, pulmonary, psychiatric, or renal) that, based on the judgment of the Investigator, is clinically unstable, is likely to deteriorate during the course of the study, could put the patient at risk because of participation in the study, could affect the subject's ability to complete the study, or could influence the study results.
History of human immunodeficiency virus infection.

Hematologic or solid malignancy diagnosis within 5 years prior to Screening.<br>• Note: Subjects with a history of localized skin cancer, basal cell or squamous cell carcinoma, may be enrolled in the study as long as they are cancer free prior to randomization. Subjects with other localized cancers (without metastatic spread) who have previously completed their course of treatment more than 5 years prior to Screening, are not currently receiving treatment and have been in remission may be enrolled only if, in the opinion of the Investigator, there is no expectation for recurrence or further cancer treatment during the study period. Antihormonal therapy (e.g., tamoxifen) is allowed if the subject's cancer is in remission and the subject is on stable maintenance therapy to reduce their risk of recurrence.]
Any major surgery within 4 weeks of Screening.
Blood transfusion within 4 weeks of Screening.
History of brain surgery for Parkinsonism (i.e., deep-brain stimulation).
History of stem-cell treatment.
Women who are pregnant or breastfeeding.
Subjects or prisoners who are involuntarily detained or incarcerated for treatment of either a psychiatric or physical illness must not be enrolled into the study.
Any medical condition, based on the judgement of the Investigator, that would confound the ability to adequately assess safety and efficacy outcome measures
Evidence of organ dysfunction or any clinically significant deviation from normal in physical examination, vital signs, ECG, or clinical laboratory determinations beyond what is consistent with the target population.
Clinically significant abnormality on 12-lead ECG prior to study drug administration beyond what is consistent with the target population, confirmed by repeat.
QTcF (Fridericia) interval ≥ 470 msec during the Screening/Baseline period or uncontrolled arrhythmia or frequent premature ventricular contraction (PVCs) (> 5/minute) or Mobitz Type II second or third degree atrioventricular (AV) block or left bundle branch block, or right bundle branch block with a QRS duration ≥ 150 msec or intraventricular conduction defect with a QRS duration ≥ 150 msec or evidence of acute or sub-acute myocardial infarction or ischemia or other ECG findings that, in the Investigator's opinion, would preclude participation in the study.
Abnormal free T4 levels and TSH > 10 mIU/L (despite treatment) at Screening, confirmed by repeat.
Total bilirubin, alanine aminotransferase (ALT) or aspartate aminotransferase (AST) greater than 2 times the upper limit of normal (ULN), confirmed by repeat. If the patient is diagnosed as having Gilbert's syndrome, the patient can be discussed with the Medical Monitor.
Pathologic renal findings at Screening as defined by the presence of either of the following criteria:<br>• Estimated glomerular filtration rate (eGFR) according to the re- expressed abbreviated (four-variable) Modification of Diet in Renal Disease (MDRD) Study equation < 30 ml/min/ 1.73m^2; The MDRD estimation is calculated as follows:<br>• eGFR (mL/min/1.73m^2) = 175 x (standardized Scr)-1.154 x (Age)-0.203 x (0.742 if female) x (1.212 if Black). [Scr: Standardized serum creatinine];<br>• Creatinine ≥ 2mg/dL.
Hematologic abnormalities at Screening:<br>Hemoglobin < 10 g/dL; orWBC < 3.0 x 103/mm^3; orPlatelet count < 100,000/mm^3; orNeutrophils, Absolute ≤ 1000/mm^3;<br>Hemoglobin A1C > 7.5%, confirmed by repeat.
Urine drug screen positive for a drug of abuse, for which the patient does not have a valid prescription and is suspected of abusing, in the judgement of the Investigator.<br>• Note: urine drug screen positive for cannabis is exclusionary unless the Investigator and Medical Monitor agree that the subject can abstain from use for the duration of the study, or if the subject has a valid medical prescription.
Human Immunodeficiency Virus (HIV) positive at Screening (indicated by positive confirmatory Western Blot).
HBsAg or HCV positive at Screening.
For WOCBP, positive serum β-hCG which is indicative of pregnancy and not false positive at Screening or a positive urine pregnancy test at Baseline.

Supplementary Table 21: Sample sizes required for a 2-arm, 6-month follow-up therapeutic trial to detect 20%, 25%, 30%, 40% and 50% change in Progressive Supranuclear Palsy and trial-eligible Progressive Supranuclear Palsy

6 Month		20% change		25% change		30% change		40% change		50% change	
	**Difference mean (SD)**	**Effect size**	**Sample size**	**Effect size**	**Sampl e size**	**Effect size**	**Sampl e size**	**Effect size**	**Sampl e size**	**Effect size**	**Sampl e size**
**PSP (Trial)**											
CBD-FS	11.6 (17.3)	0.134	875	0.168	557	0.201	390	0.268	220	0.335	141
SEADL	-13.0 (28.7)	-0.091	1897	-0.113	1230	-0.136	850	-0.181	480	-0.227	306
MS	0 (1.6)	0.003	1744192	0.004	981109	0.004	981109	0.006	436049	0.007	320363
PSP-QoL	16.5 (32.6)	0.102	1510	0.127	974	0.152	680	0.203	382	0.254	244
UPDRS II	8.2 (9.3)	0.178	496	0.223	317	0.267	221	0.356	125	0.445	80
UPDRS III	8.3 (27.7)	0.06	4361	0.075	2792	0.089	1983	0.119	1109	0.149	708
PSPRS	11.3 (14.2)	0.159	622	0.199	397	0.239	276	0.319	155	0.399	100
mPSPRS	3.0 (5.6)	0.107	1372	0.134	875	0.161	607	0.215	341	0.268	220
UPDRS I	-1.2 (6.2)	-0.04	9812	-0.05	6280	-0.06	4361	-0.08	2454	-0.1	1571
CBI-R	12.1 (34.0)	0.071	3115	0.089	1983	0.107	1372	0.143	769	0.179	491
MoCA	-0.1 (4.8)	-0.004	981109	-0.005	627910	-0.006	436049	-0.008	245278	-0.009	193800
ACE III	2.6 (7.8)	0.066	3605	0.082	2336	0.099	1603	0.132	902	0.165	578
ECAS	0.3 (17.0)	0.004	981109	0.004	981109	0.005	627910	0.007	320363	0.009	193800
FTD-FRS	7.2 (14.0)	0.103	1481	0.129	944	0.155	654	0.207	367	0.259	235
**PSP (All)**											
CBD-FS	11.8 (21.0)	0.113	1230	0.141	791	0.169	551	0.225	311	0.281	200
SEADL	-5.8 (37.6)	-0.031	16336	-0.039	10322	-0.046	7420	-0.062	4085	-0.077	2649
MS	0.3 (2.4)	0.024	27254	0.03	17443	0.036	12113	0.048	6814	0.06	4361
PSP-QoL	22.0 (34.4)	0.128	959	0.16	614	0.192	427	0.256	240	0.32	154
UPDRS II	7.3 (10.8)	0.135	862	0.168	557	0.202	386	0.269	218	0.337	139
UPDRS III	7.3 (25.4)	0.058	4667	0.072	3029	0.087	2075	0.115	1188	0.144	758
PSPRS	6.9 (16.6)	0.083	2280	0.103	1481	0.124	1022	0.165	578	0.207	367
mPSPRS	1.8 (5.6)	0.064	3833	0.081	2394	0.097	1669	0.129	944	0.161	607
UPDRS I	-0.8 (6.9)	-0.024	27254	-0.03	17443	-0.036	12113	-0.048	6814	-0.06	4361
CBI-R	13.7 (32.1)	0.085	2174	0.107	1372	0.128	959	0.171	538	0.213	347
MoCA	-0.7 (5.8)	-0.024	27254	-0.03	17443	-0.036	12113	-0.048	6814	-0.06	4361
ACE III	-0.9 (9.8)	-0.018	48451	-0.023	29675	-0.027	21534	-0.036	12113	-0.045	7753
ECAS	-7.8 (21.7)	-0.072	3029	-0.09	1939	-0.108	1347	-0.144	758	-0.18	485
FTD-FRS	7.1 (14.2)	0.099	1603	0.124	1022	0.149	708	0.199	397	0.248	256

Variables with fewer than 5 responses per group have been removed. Abbreviations: ACE III = Addenbrookes Cognitive Examination III, CBD-FS = Corticobasal Degeneration Functional Scale, CBI-R = Cambridge Behavioural Inventory Revised, CBS = corticobasal syndrome , ECAS = Edinburgh Cognitive and Behavioural Screen, FTD-FRS = Frontotemporal Dementia Rating Scale, IDT = Indeterminate, MoCA = Montreal Cognitive Assessment, mPSPRS = modified Progressive Supranuclear Palsy Rating Scale, PSP = progressive supranuclear palsy, PSP QoL = PSP Quality of life scale, PSPRS = PSP rating scale, SD = standard deviation, SEADL = Schwab and England Activities of Daily Living Scale, UPDRS = Unified Parkinson's Disease Rating Scale

Supplementary Table 22: Sample sizes required for a 2-arm, 6-month follow-up therapeutic trial to detect 20%, 25%, 30%, 40% and 50% change in Multiple System Atrophy and trial-eligible Multiple System Atrophy

6 Month		20% change		25% change		30% change		40% change		50% change	
	**Difference mean (SD)**	**Effect size**	**Sample size**	**Effect size**	**Sample size**	**Effect size**	**Sample size**	**Effect size**	**Sample size**	**Effect size**	**Sample size**
**MSA (Trial)**											
SEADL	-15.0 (18.4)	-0.163	592	-0.204	378	-0.244	265	-0.326	149	-0.407	96
MS	1.0 (1.8)	0.112	1252	0.141	791	0.169	551	0.225	311	0.281	200
MSA-QoL	12.1 (21.7)	0.111	1275	0.139	813	0.167	564	0.223	317	0.279	203
UPDRS II	1.0 (9.7)	0.021	35597	0.026	23222	0.032	15331	0.042	8900	0.053	5589
UMSARS	10.8 (17.5)	0.123	1039	0.154	663	0.185	460	0.246	260	0.308	166
UPDRS I	-2.2 (6.0)	-0.075	2792	-0.093	1816	-0.112	1252	-0.149	708	-0.187	450
CBI-R	-0.1 (22.3)	-0.001	15697720	-0.001	15697720	-0.001	15697720	-0.001	15697720	-0.002	3924431
MoCA	-1.9 (4.4)	-0.087	2075	-0.109	1322	-0.131	916	-0.174	519	-0.218	331
ACE III	-1.8 (11.4)	-0.032	15331	-0.04	9812	-0.048	6814	-0.064	3833	-0.08	2454
ECAS	-6.3 (10.9)	-0.115	1188	-0.144	758	-0.173	525	-0.231	295	-0.288	190
FTD-FRS	0.8 (9.1)	0.017	54318	0.021	35597	0.025	25117	0.033	14416	0.042	8900
**MSA (All)**											
SEADL	-13.2 (24.0)	-0.11	1298	-0.138	825	-0.165	578	-0.22	325	-0.275	209
MS	1.1 (1.8)	0.122	1056	0.152	680	0.183	470	0.244	265	0.305	170
MSA-QoL	5.7 (24.4)	0.046	7420	0.058	4667	0.07	3205	0.093	1816	0.116	1168
UPDRS II	2.2 (11.0)	0.039	10322	0.049	6539	0.059	4511	0.079	2516	0.098	1635
UMSARS	10.3 (15.1)	0.136	850	0.17	544	0.204	378	0.272	213	0.34	137
UPDRS I	-1.8 (5.4)	-0.069	3298	-0.086	2123	-0.103	1481	-0.137	837	-0.172	532
CBI-R	-0.3 (25.4)	-0.003	1744192	-0.003	1744192	-0.004	981109	-0.005	627910	-0.007	320363
MoCA	-1.4 (5.6)	-0.051	6036	-0.064	3833	-0.077	2649	-0.102	1510	-0.128	959
ACE III	0.7 (11.5)	0.013	92887	0.016	61320	0.019	43485	0.026	23222	0.032	15331
ECAS	-6.9 (17.1)	-0.081	2394	-0.101	1540	-0.121	1073	-0.162	599	-0.202	386
FTD-FRS	-3.6 (10.6)	-0.068	3396	-0.085	2174	-0.102	1510	-0.136	850	-0.17	544
Variables with fewer than 5 responses per group have been removed. Abbreviations: ACE III = Addenbrookes Cognitive Examination III, CBI-R = Cambridge Behavioural Inventory Revised, ECAS = Edinburgh Cognitive and Behavioural Screen, FTD-FRS = Frontotemporal Dementia Rating Scale, MoCA = Montreal Cognitive Assessment, MSA = multiple system atrophy, MSA-C = MSA cerebellar variant, MSA-P = MSA parkinsonism variant, MSA QoL = MSA Quality of life scale, SD = standard deviation, SEADL = Schwab and England Activities of Daily Living Scale, UPDRS = Unified Parkinson's Disease Rating Scale, UMSARS = Unified MSA Rating Scale											

Supplementary Table 23: Sample sizes required for a 2-arm, 12-month follow-up therapeutic trial to detect 20%, 25%, 30%, 40% and 50% change in trial-eligible Progressive Supranuclear Palsy and Multiple System Atrophy

12 Month		20% change		25% change		30% change		40% change		50% change	
	Difference mean (SD)	Effect size	Sample size	Effect size	Sampl e size	Effect size	Sampl e size	Effect size	Sampl e size	Effect size	Sampl e size
**PSP (Trial)**											
CBD-FS	7.8 (7.8)	0.200	393	0.250	252	0.3	175	0.400	99	0.500	64
SEADL	-16.9 (18.3)	-0.185	460	-0.231	295	-0.278	204	-0.370	116	-0.463	74
MS	0.9 (1.3)	0.137	837	0.171	538	0.205	374	0.273	212	0.342	135
PSP-QoL	16.1 (19.8)	0.162	599	0.203	382	0.243	267	0.325	150	0.406	96
UPDRS II	7.2 (5.8)	0.248	256	0.310	164	0.372	114	0.496	65	0.620	42
UPDRS III	9.6 (13.6)	0.142	779	0.177	502	0.213	347	0.283	197	0.354	126
PSPRS	12.2 (8.5)	0.286	193	0.357	124	0.429	86	0.572	49	0.715	32
mPSPRS	4.1 (3.1)	0.264	226	0.330	145	0.396	101	0.529	57	0.661	37
UPDRS I	0.3 (3.5)	0.017	54318	0.021	35597	0.025	25117	0.033	14416	0.042	8900
CBI-R	14.0 (21.6)	0.13	930	0.162	599	0.194	418	0.259	235	0.324	151
MoCA	-0.5 (2.8)	-0.036	12113	-0.045	7753	-0.054	5384	-0.072	3029	-0.09	1939
ACE III	-0.9 (4.8)	-0.038	10872	-0.048	6814	-0.058	4667	-0.077	2649	-0.096	1704
ECAS	-1.2 (10.8)	-0.021	35597	-0.027	21534	-0.032	15331	-0.043	8491	-0.053	5589
FTD-FRS	4.3 (8.8)	0.098	1635	0.122	1056	0.146	737	0.195	414	0.244	265
**MSA (Trial)**											
SEADL	-12.3 (13.8)	-0.178	496	-0.223	317	-0.267	221	-0.356	125	-0.446	80
MS	0.7 (1.0)	0.157	638	0.197	405	0.236	283	0.315	159	0.393	103
MSA-QoL	3.4 (13.8)	0.049	6539	0.061	4220	0.073	2947	0.097	1669	0.122	1056
UPDRS II	3.0 (6.0)	0.101	1540	0.127	974	0.152	680	0.203	382	0.254	244
UMSARS	10.5 (10.0)	0.209	360	0.261	231	0.313	161	0.418	91	0.522	59
CBI-R	11.1 (20.8)	0.107	1372	0.133	888	0.16	614	0.213	347	0.267	221
MoCA	-0.2 (2.7)	-0.013	92887	-0.017	54318	-0.02	39245	-0.027	21534	-0.033	14416
ACE III	-0.5 (6.7)	-0.016	61320	-0.02	39245	-0.024	27254	-0.032	15331	-0.04	9812
ECAS	-0.5 (8.1)	-0.011	129734	-0.014	80091	-0.017	54318	-0.022	32434	-0.028	20024
FTD-FRS	-0.1 (5.4)	-0.004	981109	-0.005	627910	-0.006	436049	-0.008	245278	-0.01	156978

Variables with fewer than 5 responses per group have been removed. Abbreviations: ACE III = Addenbrookes Cognitive Examination III, CBD-FS = Corticobasal Degeneration Functional Scale, CBI-R = Cambridge Behavioural Inventory Revised, CBS = corticobasal syndrome , ECAS = Edinburgh Cognitive and Behavioural Screen, FTD-FRS = Frontotemporal Dementia Rating Scale, IDT = Indeterminate, MoCA = Montreal Cognitive Assessment, mPSPRS = modified Progressive Supranuclear Palsy Rating Scale, PSP = progressive supranuclear palsy, PSP QoL = PSP Quality of life scale, PSPRS = PSP rating scale, SD = standard deviation, SEADL = Schwab and England Activities of Daily Living Scale, UPDRS = Unified Parkinson's Disease Rating Scale

<u>Supplementary Table 24:</u> Sample sizes required for a 2-arm, 12-month follow-up therapeutic trial to detect 20%, 25%, 30%, 40% and 50% change in imaging markers of trial-eligible Progressive Supranuclear Palsy and Multiple System Atrophy

**12 Month**		**20% change**		**25% change**		**30% change**		**40% change**		**50% change**	
	**Percent difference mean (SD)**	**Effect size**	**Sample size**	**Effect size**	**Sampl e size**	**Effect size**	**Sampl e size**	**Effect size**	**Sampl e size**	**Effect size**	**Sampl e size**
**PSP (Trial)**											
Temporal	-2.9 (2.1)	-0.226	308	-0.283	197	-0.339	138	-0.452	78	-0.565	50
Cingulate	-2.9 (1.8)	-0.378	111	-0.473	71	-0.567	50	-0.756	28	-0.945	19
Insula	-1.2 (2.1)	-0.129	944	-0.162	599	-0.194	418	-0.259	235	-0.323	151
Occipital	-2.6 (3.1)	-0.14	802	-0.174	519	-0.209	360	-0.279	203	-0.349	130
Frontal	-4.4 (3.3)	-0.354	126	-0.443	81	-0.532	56	-0.709	32	-0.886	21
Parietal	-3.4 (2.6)	-0.325	150	-0.407	96	-0.488	67	-0.651	38	-0.813	25
Central	-2.9 (1.9)	-0.436	84	-0.545	54	-0.654	38	-0.872	22	-1.09	14
Ventricles	11.7 (5.6)	0.444	81	0.555	52	0.666	36	0.889	21	1.111	14
Cerebellum	-3.6 (4.3)	-0.193	422	-0.242	269	-0.29	188	-0.387	106	-0.483	68
Pons	-3.8 (3.2)	-0.425	88	-0.531	57	-0.637	40	-0.849	23	-1.062	15
Midbrain	-3.0 (2.4)	-0.447	80	-0.558	51	-0.67	36	-0.893	21	-1.116	14
**MSA (Trial)**											
Temporal	-2.5 (2.7)	-0.175	514	-0.219	328	-0.263	228	-0.35	129	-0.438	83
Cingulate	-1.6 (1.1)	-0.264	226	-0.33	145	-0.396	101	-0.528	57	-0.66	37
Insula	-0.7 (1.6)	-0.081	2394	-0.101	1540	-0.121	1073	-0.161	607	-0.201	390
Occipital	-2.2 (3.4)	-0.123	1039	-0.154	663	-0.185	460	-0.247	258	-0.308	166
Frontal	-2.7 (1.6)	-0.317	157	-0.397	101	-0.476	70	-0.634	40	-0.793	26
Parietal	-2.6 (2.6)	-0.239	276	-0.298	178	-0.358	123	-0.477	70	-0.597	45
Central	-2.0 (1.2)	-0.335	141	-0.418	91	-0.502	63	-0.669	36	-0.837	23
Ventricles	7.9 (3.9)	0.351	128	0.438	83	0.526	58	0.701	33	0.877	21
Cerebellum	-4.6 (2.7)	-0.342	135	-0.427	87	-0.513	61	-0.684	35	-0.855	22
Medulla	0.001 (3.6)	-0.004	981109	-0.005	627910	-0.005	627910	-0.007	320363	-0.009	193800
Pons	-4.8 (4.5)	-0.213	347	-0.267	221	-0.32	154	-0.427	87	-0.534	56
Midbrain	-1.2 (2.0)	-0.128	959	-0.16	614	-0.192	427	-0.256	240	-0.32	154
Abbreviations: CBS = corticobasal syndrome, IDT = Indeterminate, MSA = multiple system atrophy, PSP = progressive supranuclear palsy, SD = standard deviation.											

Supplementary Methods

Fluid biomarkers

Non-fasting serum and CSF samples were processed using standardised protocols and stored in 0.5ml aliquots at -80°C within 60 minutes of sample collection. Serum NfL was measured using an ultrasensitive single molecule (Simoa) assay.[1]Where available, CSF samples were tested for total tau and amyloid beta 1-42 (Aβ1-42) levels (INNOTEST ELISA – Fujirebio Europe N.V., Gent, Belgium.[2]

Cases with corticobasal syndrome were stratified according to CSF findings using the below rationale as used in previous studies[3,4]:

Phenotype	Finding
CBS-4RT	CSF T-tau:Aβ1-42 ratio <1
CBS-AD	CSF T-tau:Aβ1-42 ratio >1
CBS-IDT	CSF not available

Neuroimaging

Scan protocols were closely matched across centres and based on the international Genetic Frontotemporal Dementia Initiative protocols (MP-RAGE, TR 2s, TE 2.93ms, Flip angle 8deg, 1.1mm isotropic).[5] T1-weighted images were processed using the recon-all pipeline of FreeSurfer 6.0.0 (Massachusetts General Hospital, Harvard Medical School; http://surfer.nmr.mgh.harvard.edu/) into subcortical segments and cortical surface parcellations with adjustments for large ventricles and additional brainstem structures parcellation. Regional analyses were performed using volume measures from 68 Desikan-Killiany atlas cortical regions and 38 subcortical volume measures from the segmentation.[6,7] Volume measures from parcellation were combined to calculate cortical grey matter volumes of the frontal, temporal, parietal, occipital lobes. Hippocampi and amygdalae volumes are included in the temporal lobe data. The remaining segmentation regions were combined into central structures (basal ganglia, thalamus, accumbens), cerebellar grey matter, brainstem, and ventricles. The images were additionally segmented into grey, white and CSF modulated probability maps using CAT12 (neuro.uni-jena.de/cat) in order to obtain total intracranial volume measures. Volumetric output data was then imported to R Studio (version 4.0.3) for further analysis including construction of linear mixed models of annual change. We also calculated the ratio of the pons to midbrain volume.

In individuals without CSF results, cases with corticobasal syndrome were stratified according to amyloid PET findings when available. Amyloid PET imaging was performed using Pittsburgh compound B ([^{11}C]PiB), and cortical standardised uptake value ratio (SUVR; 50-70 minutes post-injection; whole cerebellum reference tissue) was determined using the Centiloid Project methodology at a single centre (Cambridge).[8] The below values determined amyloid negativity as used in previous studies and were obtained by converting the Centiloid cutoff of 19 to SUVR using the Centiloid-to-SUVR transformation[9,10]:

Phenotype	Finding
CBS-4RT	[^{11}C]PiB SUVR <1.21
CBS-AD	[^{11}C]PiB SUVR >1.21
CBS-IDT	Amyloid PET not available

Genetics

DNA was extracted from serum samples of PSP, CBS and IDT cases. DNA samples subsequently underwent genotyping at UCL Institute of Child Health using the Illumina NeuroChip for white cases.[11] Standard steps taken for data quality control and single nucleotide polymorphism imputation have previously been described.[12] White control allele/haplotype frequencies were derived from dbSNP (www.ncbi.nlm.nih.gov/snp). MAPT H1/H1 (determined by rs1800547 genotype), TRIM11 (determined by rs564309 minor allele frequency), LRRK2 (determined by rs2242367 minor allele frequency) and *APOE*-ε4 allele (determined by rs429358 and rs7412 genotypes) frequencies were calculated using imputed genetic datasets.

References:

1. Kuhle J, Barro C, Andreasson U, Derfuss T, Lindberg R, Sandelius Å, et al. Comparison of three analytical platforms for quantification of the neurofilament light chain in blood samples: ELISA, electrochemiluminescence immunoassay and Simoa. Clin Chem Lab Med [Internet]. 2016 Oct 1;54(10):1655–61. Available from: http://www.ncbi.nlm.nih.gov/pubmed/27071153

2. Faull M, Ching SY, Jarmolowicz AI, Beilby J, Panegyres PK. Comparison of two methods for the analysis of CSF Aβ and tau in the diagnosis of Alzheimer's disease. Am J Neurodegener Dis [Internet]. 2014;3(3):143–51. Available from: http://www.ncbi.nlm.nih.gov/pubmed/25628965

3. Borroni B, Premi E, Agosti C, Alberici A, Cerini C, Archetti S, et al. CSF Alzheimer's disease-like pattern in corticobasal syndrome: evidence for a distinct disorder. J Neurol Neurosurg Psychiatry [Internet]. 2011 Aug;82(8):834–8. Available from: http://www.ncbi.nlm.nih.gov/pubmed/21345847

4. Jabbari E, Holland N, Chelban V, Jones PS, Lamb R, Rawlinson C, et al. Diagnosis Across the Spectrum of Progressive Supranuclear Palsy and Corticobasal Syndrome. JAMA Neurol. 2020;77(3):377–87.

5. Rohrer JD, Nicholas JM, Cash DM, van Swieten J, Dopper E, Jiskoot L, et al. Presymptomatic cognitive and neuroanatomical changes in genetic frontotemporal dementia in the Genetic Frontotemporal dementia Initiative (GENFI) study: A cross-sectional analysis. Lancet Neurol. 2015;14(3):253–62.

6. Fischl B, Salat DH, Busa E, Albert M, Dieterich M, Haselgrove C, et al. Whole brain segmentation: Automated labeling of neuroanatomical structures in the human brain. Neuron. 2002;33(3):341–55.

7. Iglesias JE, Van Leemput K, Bhatt P, Casillas C, Dutt S, Schuff N, et al. Bayesian segmentation of brainstem structures in MRI. Neuroimage. 2015;113:184–95.

8. Klunk WE, Koeppe RA, Price JC, Benzinger TL, Devous MD, Jagust WJ, et al. The Centiloid Project: standardizing quantitative amyloid plaque estimation by PET. Alzheimers Dement [Internet]. 2015 Jan;11(1):1-15.e1-4. Available from: http://www.ncbi.nlm.nih.gov/pubmed/25443857

9. Jack CR, Wiste HJ, Weigand SD, Therneau TM, Lowe VJ, Knopman DS, et al. Defining imaging biomarker cut points for brain aging and Alzheimer's disease. Alzheimers Dement [Internet]. 2017 Mar;13(3):205–16. Available from: http://www.ncbi.nlm.nih.gov/pubmed/27697430

10. Holland N, Malpetti M, Rittman T, Mak EE, Passamonti L, Kaalund SS, et al. Molecular pathology and synaptic loss in primary tauopathies: an 18F-AV-1451 and 11C-UCB-J PET study. Brain [Internet]. 2022 Mar 29;145(1):340–8. Available from: http://www.ncbi.nlm.nih.gov/pubmed/34398211

11. Blauwendraat C, Faghri F, Pihlstrom L, Geiger JT, Elbaz A, Lesage S, et al. NeuroChip, an updated version of the NeuroX genotyping platform to rapidly screen for variants associated with neurological diseases. Neurobiol Aging [Internet]. 2017;57:247.e9-247.e13. Available from: http://www.ncbi.nlm.nih.gov/pubmed/28602509

12. Reed E, Nunez S, Kulp D, Qian J, Reilly MP, Foulkes AS. A guide to genome-wide association analysis and post-analytic interrogation. Stat Med [Internet]. 2015 Dec 10;34(28):3769–92. Available from: http://www.ncbi.nlm.nih.gov/pubmed/26343929
